# Estimating indirect parental genetic effects on offspring phenotypes using virtual parental genotypes derived from sibling and half sibling pairs

**DOI:** 10.1371/journal.pgen.1009154

**Published:** 2020-10-26

**Authors:** Liang-Dar Hwang, Justin D. Tubbs, Justin Luong, Mischa Lundberg, Gunn-Helen Moen, Geng Wang, Nicole M. Warrington, Pak C. Sham, Gabriel Cuellar-Partida, David M. Evans

**Affiliations:** 1 The University of Queensland Diamantina Institute, The University of Queensland, Brisbane, Australia; 2 Department of Psychiatry, The University of Hong Kong, Hong Kong SAR, China; 3 Transformational Bioinformatics, Commonwealth Scientific and Industrial Research Organisation, Sydney, New South Wales, Australia; 4 Institute of Clinical Medicine, Faculty of Medicine, University of Oslo, Oslo, Norway; 5 Population Health Science, Bristol Medical School, University of Bristol, Bristol, United Kingdom; 6 K.G. Jebsen Center for Genetic Epidemiology, Department of Public Health and Nursing, NTNU, Norwegian University of Science and Technology, Trondheim, Norway; 7 Centre for PanorOmic Sciences, The University of Hong Kong, Hong Kong SAR, China; 8 Centre of Brain and Cognitive Sciences, The University of Hong Kong, Hong Kong SAR, China; 9 23andMe Inc, Sunnyvale, California, United States of America; 10 Medical Research Council Integrative Epidemiology Unit at the University of Bristol, Bristol, United Kingdom; Newcastle University, UNITED KINGDOM

## Abstract

Indirect parental genetic effects may be defined as the influence of parental genotypes on offspring phenotypes over and above that which results from the transmission of genes from parents to their children. However, given the relative paucity of large-scale family-based cohorts around the world, it is difficult to demonstrate parental genetic effects on human traits, particularly at individual loci. In this manuscript, we illustrate how parental genetic effects on offspring phenotypes, including late onset conditions, can be estimated at individual loci in principle using large-scale genome-wide association study (GWAS) data, even in the absence of parental genotypes. Our strategy involves creating “virtual” mothers and fathers by estimating the genotypic dosages of parental genotypes using physically genotyped data from relative pairs. We then utilize the expected dosages of the parents, and the actual genotypes of the offspring relative pairs, to perform conditional genetic association analyses to obtain asymptotically unbiased estimates of maternal, paternal and offspring genetic effects. We apply our approach to 19066 sibling pairs from the UK Biobank and show that a polygenic score consisting of imputed parental educational attainment SNP dosages is strongly related to offspring educational attainment even after correcting for offspring genotype at the same loci. We develop a freely available web application that quantifies the power of our approach using closed form asymptotic solutions. We implement our methods in a user-friendly software package **IMPISH** (**IM**puting **P**arental genotypes **I**n **S**iblings and **H**alf Siblings) which allows users to quickly and efficiently impute parental genotypes across the genome in large genome-wide datasets, and then use these estimated dosages in downstream linear mixed model association analyses. We conclude that imputing parental genotypes from relative pairs may provide a useful adjunct to existing large-scale genetic studies of parents and their offspring.

## Author summary

Indirect parental genetic effects may be defined as the influence of parental genotypes on offspring phenotypes over and above that which results from the transmission of genes from parents to children. Estimating indirect parental genetic effects on offspring outcomes at the genotype level has been challenging because it requires large-scale, individual level genotypes from both parents and their offspring, and there is a paucity of cohorts around the world with this information. Here we present a new approach to estimate indirect parental genetic effects without the requirement of physically genotyped parents. Our method creates virtual parental genotypes based on the genotypes of offspring pairs, and then uses these virtual genotypes in downstream genetic association analyses. We developed a software package “IMPISH” that allows users to impute virtual parental genotypes in their own genome-wide datasets and then use these in downstream genome-wide association analyses, as well a series of power calculators to estimate the power to detect indirect parental genetic effects on offspring phenotypes. We apply our method to educational attainment data from the UK Biobank and show that indirect parental genetic effects are related to offspring educational attainment even after correcting for offspring genotype at the same loci.

## Introduction

**Fig 1:**
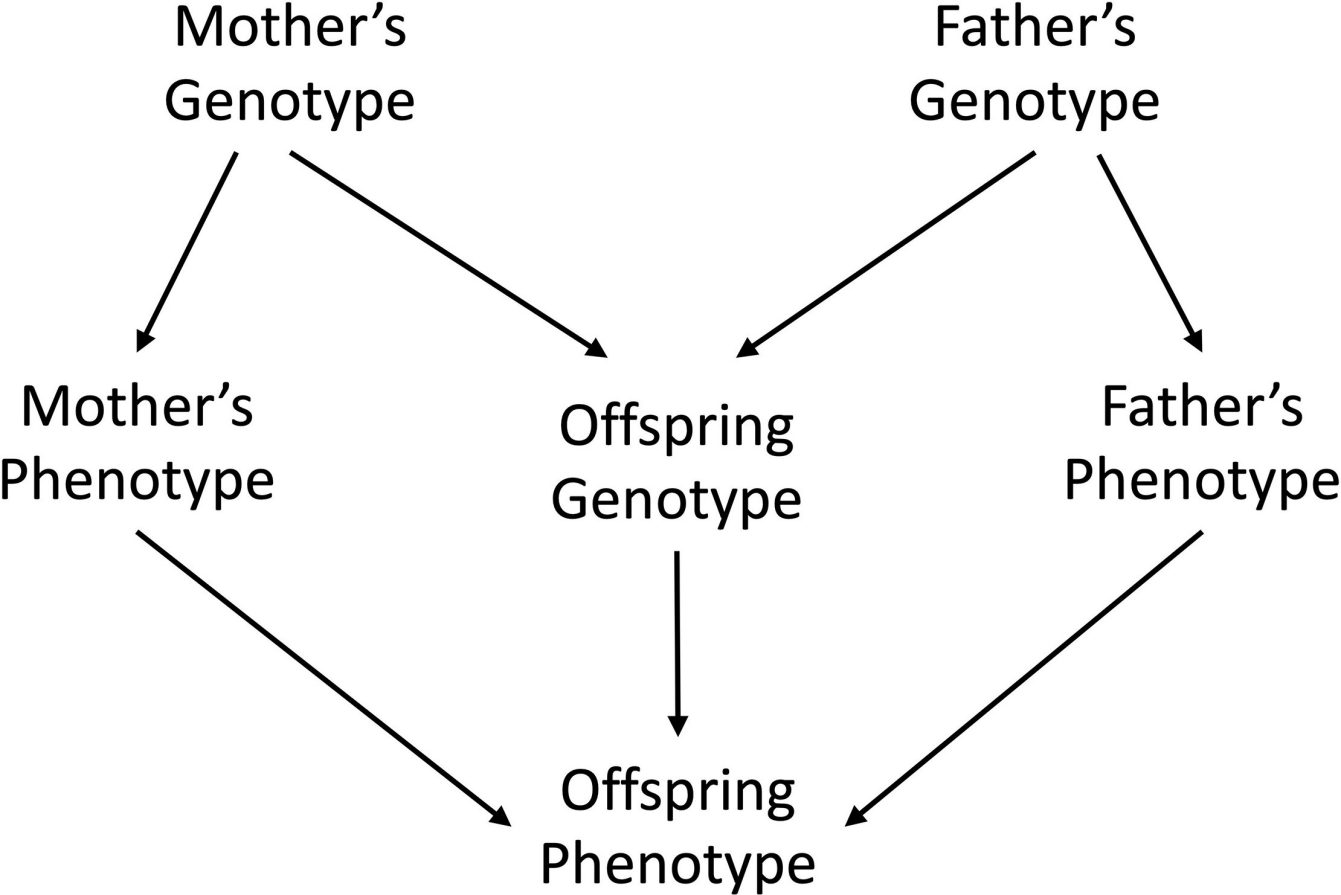
Diagram showing the relationship between mother’s, father’s and offspring’s genotypes and phenotypes. In this manuscript we refer to the path from the offspring genotype to offspring phenotype as the (direct) offspring genetic effect. We refer to the path from mother’s genotype to mother’s phenotype to offspring phenotype as an indirect maternal genetic effect (indirect because the effect of the mother’s genotype is mediated through a maternal phenotype). Likewise, we refer to the path from father’s genotype to father’s phenotype to the offspring phenotype as an example of an indirect paternal genetic effect (indirect because the effect of the father’s genotype is mediated through the paternal phenotype). Indirect maternal and paternal genetic effects are both instances of indirect parental genetic effects on offspring phenotypes. The parental phenotypes mediating these relationships may be known or unknown, may involve one or several phenotypes, and may be modelled or not in the analysis strategy. In this manuscript, we do not model the mother’s or father’s phenotype explicitly, merely the association between mother’s/father’s genotype and offspring phenotype.

Indirect parental genetic effects may be defined as the influence of parental genotypes on offspring phenotypes over and above that which results from the transmission of genes from parents to their children. This can include the effect of mother’s genotype on the offspring phenotype (“maternal genetic effects”) as well as effects of the father’s genotype on the offspring phenotype (“paternal genetic effects”). We use the term “indirect” in this context to highlight that the effect of the relevant parent’s genotype on the offspring phenotype is mediated by some known or unknown parental phenotype regardless of whether this parental trait is modelled explicitly in downstream analyses (Fig 1).

There is increasing interest in estimating the indirect effect of parental genotypes on the phenotypes of their offspring [1–7]. We and others have shown in human populations that the maternal and paternal genomes can indirectly affect a range of offspring traits including perinatal [2,8–15] and later life phenotypes [3,10]. For example, we recently showed that maternal genetic variants associated with type 2 diabetes in mothers were also associated with birthweight of their offspring, presumably through their effect on circulating maternal glucose and other factors in the intrauterine environment [11]. However, these sorts of analyses typically require large numbers of genotyped parent-offspring duos and trios in order to partition genetic effects into parental and offspring mediated components [2,16]. Unfortunately, there are only a few cohorts around the world with large numbers of genotyped parents and children [17–21] implying that for most studies, the statistical power to resolve parental genetic effects on offspring phenotypes is limited [16]. The problem of low statistical power is exacerbated further if the interest is on identifying parental genetic effects on late onset diseases, since many of the cohorts that contain genotypic information on parents and their children are birth cohorts that were established less than thirty years ago [17,18,20]. This means that offspring from these cohorts are not old enough to have developed many late onset diseases of interest. There is therefore a considerable need to develop statistical genetics methods and software that can maximize the amount of data available to detect indirect parental genetic effects on offspring traits [2].

In this manuscript, we describe a simple strategy for estimating indirect parental genetic effects on offspring phenotypes which is capable of leveraging the considerable information contained within large publicly available cohorts and the tens of thousands of individuals contained within twin registries and family studies from around the world [22]. Briefly, our strategy involves creating “virtual” mothers and fathers by estimating the genotypic dosages of parental genotypes using physically genotyped data from sibling and half sibling relative pairs. We then use the expected dosages of the parents, and the actual genotypes of the siblings/half sibling pairs to perform conditional genetic association analyses and estimate maternal, paternal and offspring genetic effects on the offspring phenotype.

We derive formulae to impute the expected dosage of maternal and paternal genotypes given sibling or half-sib genotypes at both autosomal and X-linked loci. We implement our calculations in a user-friendly software package, **IMPISH** (**IM**puting **P**arental genotypes **I**n **S**iblings and **H**alf siblings) that allows users to quickly and efficiently impute parental genotypes across the genome in large genome-wide datasets, and then use these estimated dosages in downstream genome-wide association analyses (http://evansgroup.di.uq.edu.au/software.html). We investigate the statistical power, type 1 error and bias associated with estimating parental and offspring genetic effects via simulation and using closed form asymptotic solutions. We develop a series of freely available web applications (http://evansgroup.di.uq.edu.au/power-calculators.html) that researchers can use to estimate power to detect parental and offspring genetic effects in studies of sibling or half sibling pairs, with or without parental genotypes. Finally, we apply our methods to educational attainment data from 19066 sibling pairs from the UK Biobank Study [21].

## Methods

### Trait-genotype models

In the case of sibling pairs at autosomal loci, we assumed that trait values are generated according to the following model:

$$Y_{1i}=bX_{1i}+dX_{m,i}+fX_{f,i}+\tau_{i}+\varepsilon_{1i}$$

$$Y_{2i}=bX_{2i}+dX_{m,i}+fX_{f,i}+\tau_{i}+\varepsilon_{2i}$$

$$X_{1i}=0.5(X_{m,i}+X_{p,i})+\eta_{1i}$$

$$X_{2i}=0.5(X_{m,i}+X_{p,i})+\eta_{2i}$$

where *Y*_*1*_ and *Y*_*2*_ are the phenotypes of siblings one and two, *X*_*1*_, *X*_*2*_, *X*_*m*_ and *X*_*f*_ are the genotype dosages of siblings one and two and their mother and father respectively, *b*, *d* and *f* are the effect of the offspring, mother’s and father’s genotypes respectively on the offspring phenotype, *τ* is a random effect shared by the siblings, *ε*_1_ and *ε*_2_ are uncorrelated error terms for the two phenotypes, and *η*_1_ and *η*_2_ are random effects due to the segregation of alleles (or stated another way, *η*_1_ and *η*_2_ represent the deviation of sibling one and sibling two’s dosage from the expected offspring genotypic dosage given the parental genotypes. Readers unfamiliar with the concept of segregation variance are directed to Wang and Xu (2019) for a lucid explanation of these terms and their derivation) [23]. In all cases, the subscript i refers to the *i*th family. Without loss of generality, we assume the variance of the genotype dosages and phenotype terms is one. The variances of the random effects are:

$$Var(\varepsilon_{1})=Var(\varepsilon_{2})=\sigma^{2}$$

$$Var(\tau )=\varphi^{2}$$

Var(η)=0.5

In the case of X chromosome loci for sibling pairs, we assume that the effect of genotypes on offspring phenotype are equal in males and females (i.e. the coefficients *b*, *d* and *f* are equal regardless of whether the sibling is male or female). We assume that female genotypes are standardized to unit variance whilst male genotypes have twice this variance and consequently explain double the variance in the offspring phenotype. We assumed sibling phenotypes at X chromosomal loci are generated according to the following model:

$$Y_{1i}=bX_{1i}+dX_{m,i}+fX_{f,i}+\tau_{i}+\varepsilon_{1i}$$

$$Y_{2i}=bX_{2i}+dX_{m,i}+fX_{f,i}+\tau_{i}+\varepsilon_{2i}$$

$$X_{1i}=X_{m,i}+\eta_{Mi}$$

$$X_{2i}=0.5X_{m,i}+{0.5X}_{f,i}+\eta_{Fi}$$

where the terms are defined similar to the sibling model above where sibling one is male and sibling two is female, and *η*_M_ and *η*_F_ are random effects due to segregation in male and female offspring. The variances of the random effects are:

$$Var(\varepsilon_{1})=\sigma_{\varepsilon 1}^{2}$$

$$Var(\varepsilon_{2})=\sigma_{\varepsilon 2}^{2}$$

$$Var(\tau )=\varphi^{2}$$

$$Var(\eta_{M})=1$$

$$Var(\eta_{F})=0.25$$

At X chromosome loci, the covariances between genotype dosages of relative pairs are sex-dependent:

Mother-Daughter:

$$\mathrm{Cov}\left(X_{2},X_{m}\right)=0.5$$

Mother-Son:

$$\mathrm{Cov}\left(X_{1},X_{m}\right)=1$$

Father-Daughter:

$$\mathrm{Cov}\left(X_{2},X_{f}\right)=1$$

Father-Son:

$$\mathrm{Cov}\left(X_{1},X_{f}\right)=0$$

Brother-Brother:

$$\mathrm{Cov}\left(X_{1},X_{2}\right)=1$$

Sister-Sister:

$$\mathrm{Cov}\left(X_{1},X_{2}\right)=0.75$$

Brother-Sister:

$$\mathrm{Cov}\left(X_{1},X_{2}\right)=0.5$$

and so there are three separate models for female-female, male-male, and opposite sex sibling pairs (see S1 Text for more details).

Finally, in the case of (maternal) half sibling pairs (i.e. half siblings who share a common mother) at autosomal loci we assume the model:

$$Y_{1i}=bX_{1i}+dX_{m,i}+fX_{f1,i}+\tau_{i}+\varepsilon_{1i}$$

$$Y_{2i}=bX_{2i}+dX_{m,i}+fX_{f2,i}+\tau_{i}+\varepsilon_{2i}$$

$$X_{1i}=0.5(X_{m,i}+X_{f1,i})+\eta_{1i}$$

$$X_{2i}=0.5(X_{m,i}+X_{f2,i})+\eta_{2i}$$

where the subscripts *f1* and *f2* denote the fathers of half sibling one and half sibling two respectively. The variances of the random effects are

$$Var(\varepsilon_{1})=Var(\varepsilon_{2})=\sigma^{2}$$

$$Var(\tau )=\varphi^{2}$$

Var(η)=0.5

Paternal half sibling pairs can be parameterized analogously. The reason we don’t show this explicitly is that for autosomal loci, the power to detect maternal effects using paternal half sibling pairs is the same as the power to detect paternal effects using maternal half sibling pairs, and the power to detect paternal effects using paternal half sibling pairs is the same as the power to detect maternal effects using maternal half sibling pairs.

### Imputing expected gene dosages for parents given observed offspring genotypes

**Fig 2:**
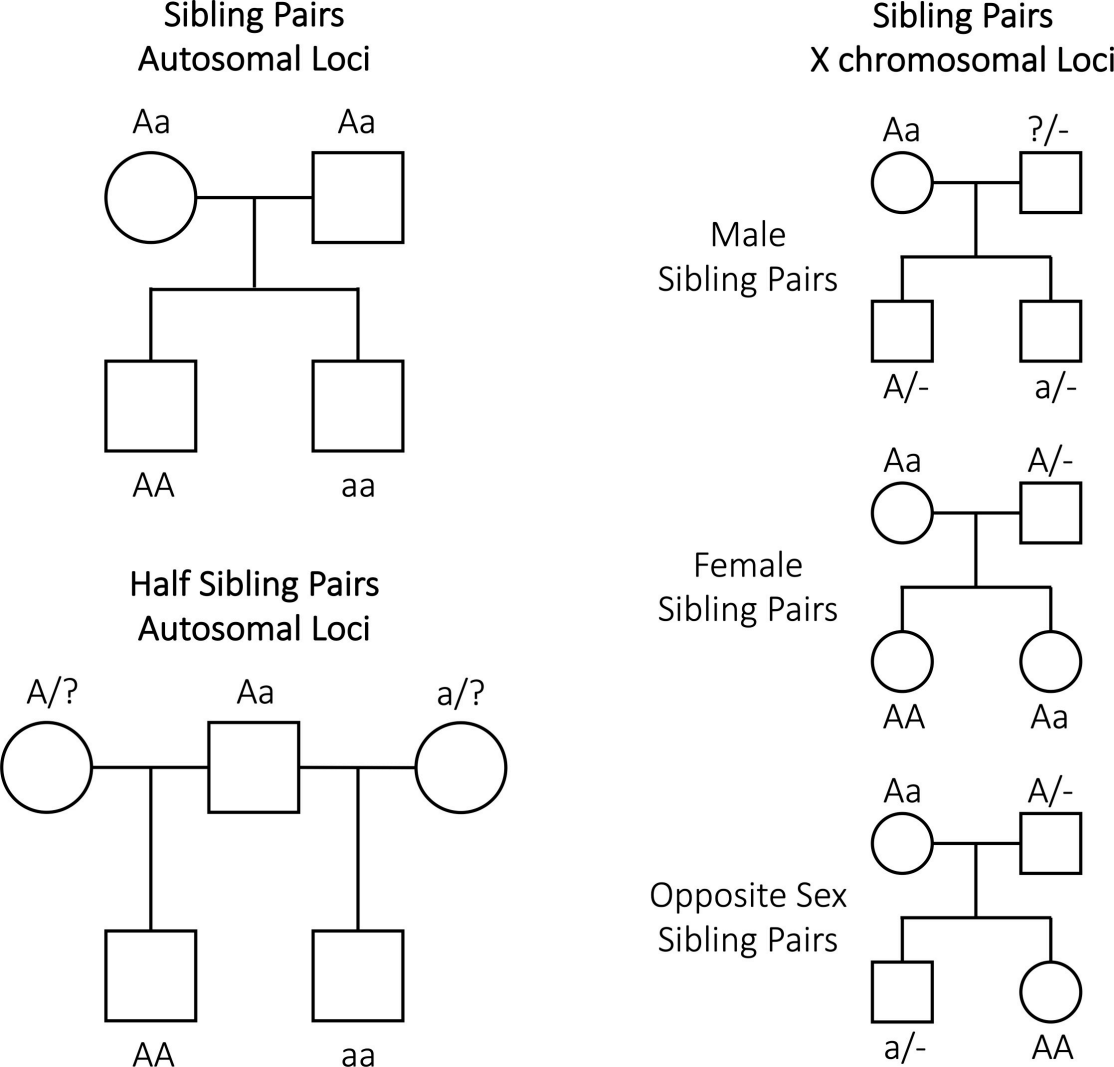
Illustration showing the intuition behind why the genotypes of relative pairs such as siblings and half siblings provide information on parental genotypes. In the case of sibling pairs at autosomal loci, sibling genotypes provide information on parental genotypes. However, mothers and fathers have the same expected genotypes and so separate genotypes for mothers and fathers cannot be imputed given only genotype information from sibling pairs. However, mothers and fathers have different expected genotypes given sibling pair genotypes at non-autosomal X chromosome loci, and so different parental genotypes can be imputed at these loci. Likewise, in the case of half sibling pairs, mothers and fathers have different expectations for their genotypes given half sibling genotypes, and so different dosages for the parents can be imputed at loci. Male individuals are uninformative for the genotypes of their fathers at (non-pseudoautosomal) X chromosome loci.

Unfortunately, parental genotypes are not always available and so the models described above cannot always be fit to the data. In these situations, it may be possible to impute parental genotype dosages using information from relative pairs (like siblings or half siblings) and subsequently include these imputed dosages in downstream analyses. The intuition for why relative pairs enable imputation of parental genotypes is illustrated in Fig 2. Essentially, an individual’s sibling/half sibling provides additional information on the likely genotype of their parents- so that some parental genotypes are more probable than others given the observed genotype data. For example in Fig 2, it is possible to conclude that both parents of siblings who have genotypes “AA” and “aa” at an autosomal locus must be heterozygous. Likewise, maternal half siblings whom have genotype “AA” and “aa” at an autosomal locus, imply that their shared mother must be genotype “Aa” and their fathers “AA” or “Aa” and “Aa” or “aa” respectively (the exact probabilities depending on the allele frequencies at the locus under consideration). We calculated the probability of maternal and paternal biallelic SNP genotypes given data from sibling pairs or half sibling pairs at the same locus. We did this for autosomal and non-pseudoautosomal X chromosome loci for biallelic SNP markers using Bayes Theorem e.g. for an autosomal locus:

$$Prob(G_{P}=i|G_{1}=j,G_{2}=k)=\frac{Prob(G_{1}=j,G_{2}=k|G_{P}=i)Prob(G_{P}=i)}{Prob(G_{1}=j,G_{2}=k)}$$

where *G*_*P*_ ∈ {“AA”, “Aa”, “aa”} refers to the genotype of the parent, and *G*_*1*_ and *G*_*2*_ the genotypes of offspring one and two. Given conditional genotype probabilities, it is then a simple matter to calculate the expected (unstandardized) genotype dosages of the parents for a given pair of offspring genotypes:

$$Expected\;Dosage=Prob(G_{P}=Aa|G_{1}=j,G_{2}=k)+2\times Prob(G_{P}=aa|G_{1}=j,G_{2}=k)$$

In the case of full sibling pairs, separate maternal and paternal genotypes can be resolved for X-linked loci. However, in the case of autosomal loci, the expected dosage for maternal and paternal genotypes is the same, meaning that it is impossible to distinguish maternal from paternal genotypes. In these situations, it is still possible to calculate a single imputed parental dosage (and include this term in downstream regression models in which case the combined effect of maternal and paternal genetic effects *c* = *d* + *f* on the offspring phenotype will be estimated—see Results section for further details).

Derivations of expected parental genotype dosages given sibling/half sibling genotypes for autosomes and the X chromosome are given in S1 Table, S2 Table, S3 Table, and S4 Table. In all derivations we use the subscript *m* to refer to the mother’s genotype (“m” for mothers), the subscript *f* to refer to father’s genotype (“f” for fathers), and the subscript *p* to refer to the estimated parental genotype (“p” for parental- typically when the mother’s and father’s genotypes cannot be distinguished as is the case for autosomal loci in sibling pairs).

### Statistical model for testing association

In order to obtain estimates of maternal, paternal and offspring genetic effects on the offspring phenotype, mother’s and/or father’s expected dosages can be included as terms in the fixed effects part of a linear mixed model together with the observed dosages of the offspring genotypes and a random effects term for family membership. In the case of sibling pairs at autosomal SNPs, we investigated the properties of the following tests of association:

1. Omnibus test: We compared the full model where free terms for the offspring and parental genetic effect(s) were estimated versus a model where the offspring and parental regression coefficient(s) were fixed to zero (i.e. either a three degree of freedom test if genotypes for both parents were available (comparing a model with free *b*, *d* and *f* to a model with these three parameters fixed to zero) or a two degrees of freedom test if a single parental genotype was imputed (comparing a model with free parental and offspring genetic effects to a model with these two parameters fixed to zero)).
2. Test using offspring genotypes only: We compared a model where there was a free term for the offspring genetic effect only (*b*), against a model where this term was set to zero (i.e. a one degree of freedom test). In other words, the effect of parental genotypes was not modelled in this analysis, even though parental genetic effects may influence the offspring phenotypes and parental genotypes may or may not be present.
3. Test of the offspring genetic effect: We compared the full model where free terms for the offspring and parental genetic effect were estimated versus a model where the offspring regression coefficient was fixed to zero (i.e. a one degree of freedom test).
4. Test of the parental genetic effect: We compared the full model where free terms for the offspring and parental genetic effect were estimated versus a model where the imputed parental regression coefficient was fixed to zero (i.e. a one degree of freedom test).

In the case of half sibling pairs, as well as sibling pairs at X chromosome SNPs, we investigated the properties of the following tests of association:

1. Omnibus test: We compared the full model where free terms for offspring, maternal and paternal genetic effects are estimated versus a model where the offspring, maternal and paternal regression coefficients were fixed to zero (i.e. comparing a model with free *b*, *d* and *f* to a model with these three parameters fixed to zero- a three degrees of freedom test).
2. Test using offspring genotypes only: We compared a model where there was a free term for the offspring genetic effect only (*b*), against a model where this term was set to zero (a one degree of freedom test). In other words, the effect of parental genotypes were not modelled in this analysis, even though maternal and/or paternal genetic effects may influence the offspring phenotypes and parental genotypes may or may not be present.
3. Test of the offspring genetic effect: We compared the full model where free terms for offspring, maternal and imputed paternal genetic effects are estimated versus a model where the offspring regression coefficient was fixed to zero (i.e. comparing a model with free *b*, *d*, and *f* to a model with *d* and *f* estimated and *b* fixed to zero- a one degree of freedom test).
4. Test of the maternal genetic effect: We compared the full model where free terms for offspring, maternal and paternal genetic effects are estimated versus a model where the maternal regression coefficient was fixed to zero (i.e. comparing a model with free *b*, *d* and *f* to a model with *b* and *f* estimated and *d* fixed to zero a one degree of freedom test).
5. Test of the paternal genetic effect: We compared the full model where free terms for offspring, maternal and paternal genetic effects are estimated versus a model where the paternal regression coefficient was fixed to zero (i.e. comparing a model with free *b*, *d*, and *f* to a model with *d* and *b* estimated and *f* fixed to zero- a one degree of freedom test).

In the case of the Omnibus test (Model 1) and the test using the offspring genotypes only (Model 2), the focus is on locus detection (i.e. whether there is a genetic effect at the locus, regardless of whether it is mediated through the offspring or parental genomes). In contrast, in the case of the tests for the parental, maternal, paternal or offspring genetic effects (Models 3 to 5), the focus is on partitioning a known locus into its indirect parental genetic and/or offspring genetic components. These tests are more relevant if the goal is to determine which genome mediates a known genetic effect on the offspring phenotype, or if the objective is on deriving unbiased effect estimates of genetic effects e.g. for Mendelian randomization analyses.

### Calculating power analytically using the non-centrality parameter

We derived closed form expressions for the non-centrality parameter of the statistical tests described above for actual and imputed parental genotypes and confirmed the results of these against simulations (see below). We have implemented these asymptotic power calculations in a series of applications which are freely available on our website (http://evansgroup.di.uq.edu.au/power-calculators.html). In the results section, we use our utilities to compare the statistical power to detect genetic effects when parental genotypes are available and when they need to be imputed for both sibling and half sibling pairs.

### Exploring parameter bias, power and type 1 error of tests of genetic association via simulation

In order to confirm our asymptotic results, we investigated parameter bias, power and type 1 error rate of tests of genetic association via simulation. Genotypes were simulated for nuclear families (mother, father and two siblings) and maternal half sibling families (common mother, two fathers and two half siblings). For our simulations, we varied the size of genetic effects (three conditions: *b*^*2*^ = *d*^*2*^ = *f*^*2*^ = 0; *b*^*2*^ = 0.1%, *d*^*2*^ = *f*^*2*^ = 0.05%; *b*^*2*^ = *d*^*2*^ = *f*^*2*^ = 0.1%), frequency of the trait decreasing allele (three conditions: *p* = 0.1, *p* = 0.5, *p* = 0.9), and shared residual variance (two conditions: *φ*^2^ = 0; *φ*^2^ = 0.2). For all simulations we used N = 2000 sibling pairs/half sibling pairs, a type 1 error rate of α = 0.05, and 1000 replications. For each condition, we assumed that either parental genotypes were available, or they were not, in which case we calculated the expected genotype dosages of the parents using their offspring genotypes based on the formulae from the preceding section (S1 Table, S2 Table, S3 Table, S4 Table). Offspring phenotype was then regressed on offspring genotype, and imputed (or physically genotyped) parental dosages using the *lmer* package in R. Family was included as a random effect in these analyses. Tests were conducted using full information maximum likelihood. R code implementing the simulations is provided in the S1 Text.

### Effect of differential missing rates on power, bias and type 1 error rate

We investigated the sensitivity of our method to situations where parental genotypes were not missing completely at random and needed to be imputed. This might occur for example where a locus is associated with premature mortality, in which case parents with the risk genotype are more likely to be missing and so need to be imputed. We assumed that the probability that parents were missing was related to the genotype at the locus needing to be imputed. We performed simulations where the relationship between parental genotype and risk of missingness was mutiplicative (i.e. Prob(genotype AA is missing) = 20%, Prob(genotype Aa is missing) = 30%; Prob(genotype aa is missing) = 40%), dominant (i.e. Prob(genotype AA is missing) = 20%, Prob(genotype Aa is missing) = 40%; Prob(genotype aa is missing) = 40%), or recessive (i.e. Prob(genotype AA is missing) = 20%, Prob(genotype Aa is missing) = 20%; Prob(genotype aa is missing) = 40%). We assumed that the base allele frequency in the population was either p = 0.1 or 0.5, the shared residual variance between sibling phenotypes was *φ*^2^ = 0 or *φ*^2^ = 0.5, and we varied the size of maternal and offspring genetic effects (three conditions: *b*^*2*^ = *d*^*2*^ = 0%; *b*^*2*^ = 1%, *d*^*2*^ = 0; *b*^*2*^ = *d*^*2*^ = 1%). For all conditions, we simulated N = 2000 sibling pairs, all with missing parental genotypes, and performed 1000 replications where we estimated an offspring genetic effect and a parental (maternal) genetic effect. We examined bias, power and type 1 error (α = 0.05) and compared this to asymptotic power calculations where parental genotypes were missing completely at random. It is worth noting that these simulations represent rather extreme situations in that in real data we would rarely expect missing rates to be so strongly related to a single genotype, particularly in the case of complex traits- but are useful to get some idea of the sensitivity of our method to substantial deviations from underlying assumptions.

### Application to educational attainment in the UK biobank

In order to illustrate the potential utility of our procedure we imputed the parental genotypes of 19066 full sibling pairs of white British ancestry from the UK Biobank with educational attainment data (N = 18761 sibling pairs whom both reported educational attainment, and N = 305 sibling pairs where only one sibling reported educational attainment) at 1264 SNPs known to be robustly associated with educational attainment with minor allele frequency > 0.01 (S5 Table) [24]. We constructed unweighted polygenic risk score dosages for individuals using all SNPs (using either physically genotyped SNPs in the case of sibling pairs or imputed SNP dosages in the case of their imputed parents) oriented to the increasing allele for educational attainment. We used an unweighted polygenic score rather than a weighted score in analyses because it was not clear from the original educational attainment GWAS what portion of the reported SNPs’ effects on educational attainment was due to parental genetic effects and what portion was due to offspring genetic effects. Educational attainment was measured by self-report according to the following coding (4 = College or University degree; 3 = professional qualifications; 2 = A levels; 1 = O levels; 0 = None of the above). We regressed educational attainment on own polygenic score and imputed parental polygenic score including the top five GWAS derived principal components, sex, and year of birth as fixed effects and family as a random effect using the *lme* package in R. As a sensitivity analysis, we performed similar analyses, but using only 72 out of the 74 genome-wide significant SNPs that were available in our data from the Okbay et al (2016) GWAS of educational attainment (i.e. which did not contain the UK Biobank in the discovery analyses) to construct the unweighted genetic scores [25]. We also compared our results to similar analyses using ordinary least squares regression analyses involving N = 4071 mother-offspring duos, N = 1809 father-offspring duos and a set of N = 1064 parent-offspring trios from the UK Biobank where the offspring had reported their educational attainment (this latter analysis involving trios comprised individuals from the mother-offspring and father-offspring duos analyses). The set of covariates used in these latter analyses was the same except that parental polygenic risk score was not imputed and based on physically genotyped SNPs.

### Software to impute parental genotypes

We have coded the parental imputation routines described above in a C++ software package called **IMPISH** (**IM**puting **P**arental genotypes in **S**iblings and **H**alf siblings) which is freely available on our website (http://evansgroup.di.uq.edu.au/software.html). IMPISH uses source code adapted from the GCTA software package (version 1.26.0) that has been modified to impute parental genotype data given genotypes from sibling or half sibling pairs [26]. IMPISH accepts data in the form of PLINK style binary.bed,.bim and.fam file formats [27]. Users can elect to output expected parental genotype dosages in PLINK dosage format or have the software compute these internally and utilize them in genome-wide association testing. IMPISH calculates allele frequencies from the (sibling/half sibling) data that the user provides, and calculates genotype frequencies and expected genotypes assuming Hardy-Weinberg equilibrium. IMPISH fits a genetic mixed linear model with fixed effects for offspring genotype and (imputed) mother’s and father’s genotypes and allows users to compute these statistics across the genome in a computationally efficient fashion. A genome-wide genetic relationship matrix is used in the random effects part of the model just as in the original GCTA software, allowing users to account for population stratification and cryptic relatedness in their analyses. If users choose, they can also include a relationship matrix in the random effects part of the model that specifies the correlation between individuals in terms of the family environment (i.e. ones down the main diagonal and ones in elements (*i*, *j*) and (*j*, *i*) of the matrix if individuals *i* and *j* are from the same family, zeros elsewhere in the matrix) although this matrix will need to be constructed and input by the user. Currently, IMPISH only performs analyses on sibling pairs/half sibling pairs that have had their parental genotypes imputed, and will remove other individuals from the analysis automatically.

To quantify the computational requirements of the **IMPISH** software, we simulated datasets that ranged in size from *N* = 1,000 to 20,000 sibling pairs and *M* = 500,000 autosomal SNP markers. The datasets were simulated using an approach similar to that described above. We benchmarked the running time and memory use of the **IMPISH** software by running simulations on these datasets. Reported runtimes are the medians of five identical runs in a computing environment with 256 GB memory and 1 CPU core with solid-state disk in one compute node.

## Results

### Derivation of non-centrality parameters and asymptotic power for tests of association in sibling pairs (autosomal loci)

Under full information maximum likelihood, all the tests of association considered in this manuscript are distributed as non-central chi-square distributions under the alternative hypothesis of genetic association, with degrees of freedom equal to the difference in the number of free parameters between full and reduced models. The non-centrality parameter (*ζ*) of these distributions is equal to twice the difference in expected log-likelihoods between the full and reduced models. Given the non-centrality parameter (*ζ*) of the statistical test, the power to detect association (P) can be obtained by the formula:

$$P=\int_{\chi_{\alpha }^{\prime 2}(\nu ,0)}^{\infty }d\chi^{\prime 2}(\nu ,\zeta )$$

where $\chi_{\alpha }^{\prime 2}(\nu ,0)$ is the 100(1 - α) percentage point of the central *χ*^*2*^ distribution with *ν* degrees of freedom, and *χ*′^2^(*ν*,*ζ*) denotes a non-central chi-square distribution with non-centrality parameter *ζ* and degrees of freedom *ν*. In the section below, we derive the expected covariance matrix of the residuals for each statistical model and its associated expected minus two log-likelihood. From these values the non-centrality parameter and statistical power of the relevant test of association can be calculated.

To illustrate our derivations, we consider the case of sibling pairs with phenotype data *Y*_1_ and *Y*_2_, and corresponding observed genotype dosages *X*_1_ and *X*_2_, at an autosomal single nucleotide polymorphism (SNP). Similar derivations for sibling pairs at X chromosome loci and for half sibling pairs on the autosomes are provided in the S1 Text. The calculation of the genotypic dosage assumes additivity (i.e. no dominance), and without loss of generality, all genotype dosages and phenotypes are standardized to have mean 0 and variance 1. In situations where the observed genotype data of parents (i.e. father’s genotype dosage *X*_f_ and mother’s genotype dosage *X*_m_) are unavailable, mother’s and father’s genotypic dosages are imputed from the genotype dosages of the sibling pairs as:

$$X_{m}’=E(X_{m}|X_{1},X_{2})$$

$$X_{f}’=E(X_{f}|X_{1},X_{2})$$

We assume the model above for sibling pairs (see Methods) and random mating so that Cov(*X*_*m*_, *X*_*f*_) = 0. The covariances between genotype dosages are:

$$Cov\left(X_{1},X_{2}\right)=Cov\left(X_{1},X_{m}\right)=Cov\left(X_{2},X_{m}\right)=Cov\left(X_{1},X_{f}\right)=Cov\left(X_{2},X_{f}\right)=0.5$$

the covariance between phenotypes and genotype dosages are:

$$Cov(Y_{1},X_{1})=Cov(Y_{2},X_{2})=b+0.5\left(d+f\right)$$

$$Cov(Y_{1},X_{2})=Cov(Y_{2},X_{1})=0.5(b+d+f)$$

$$Cov(Y_{1},X_{m})=Cov(Y_{2},X_{m})=0.5b+d$$

$$Cov(Y_{1},X_{f})=Cov(Y_{2},X_{f})=0.5b+f$$

and the covariance between the two phenotypes is:

$$Cov\left(Y_{1},Y_{2}\right)=0.5b^{2}+d^{2}+f^{2}+bd+bf+\varphi^{2}$$

The phenotypic variance in the offspring phenotype (*Y*) can be decomposed as follows:

$$Var(Y)=(b^{2}+d^{2}+f^{2}+bd+bf)+\varphi^{2}+\sigma^{2}=1$$

Full sibling relationships do not provide any information to distinguish between alleles of maternal versus paternal origin at autosomal loci. In other words, in these situations, the mother’s imputed dosage $X_{m}^{\prime }$ is equal to the father’s imputed dosage $X_{f}^{\prime }$. Thus, when using sibling pair data at autosomal loci, only one parental genotype dosage ($X_{p}^{\prime }=X_{m}^{\prime }=X_{f}^{\prime }$) can be included in downstream regression models, in which case the parameter *c* is estimated, which equals the combined effects of *d* and *f* such that *c = d + f*.

The variance of the imputed parental genotype dosage (relative to the standardized observed genotype dosages), $Var(X_{p}^{\prime })$, imputed from full sibling pairs is derived in the S1 Text and is:

$$Var(X_{p}^{\prime })=\frac{2H^{2}+5H+12}{4(H+2)(H+4)}$$

where *H* is the expected heterozygosity, given the allele frequencies *p* and *q* = *1*—*p*:

H=2pq

The covariance between actual and imputed genotype is equal to the variance of the imputed genotype:

$$Cov(X_{m},X_{p}^{\prime })=Cov(X_{f},X_{p}^{\prime })=Var(X_{p}^{\prime })$$

and the covariances between the imputed parental genotype and sib genotypes and phenotypes are:

$$Cov(X_{p}^{\prime },X_{1})=Cov(X_{p}^{\prime },X_{2})=0.5$$

$$Cov(X_{p}^{\prime },Y_{1})=Cov(X_{p}^{\prime },Y_{2})=b/2+c\times Var(X_{p}^{\prime })$$

When actual genotypes are available for the mother and father, the linear mixed model is:

$$Y_{1i}=bX_{1i}+dX_{m,i}+fX_{f,i}+\tau_{i}+\varepsilon_{1i}$$

$$Y_{2i}=bX_{2i}+dX_{m,i}+fX_{f,i}+\tau_{i}+\varepsilon_{2i}$$

The fixed effects *b*, *d*, and *f* may be estimated by generalised least squares (GLS), where the covariance matrix of random effects is:

$$\Omega =\left(\begin{array}{cc} \sigma^{2}{+\varphi }^{2} & \varphi^{2}\\ \varphi^{2} & \sigma^{2}{+\varphi }^{2} \end{array}\right)$$

The inverse of the covariance matrix of random effects is:

$$\Omega^{-1}=\frac{1}{\sigma^{2}(\sigma^{2}{+2\varphi }^{2})}\left(\begin{array}{cc} \sigma^{2}{+\varphi }^{2} & {-\varphi }^{2}\\ {-\varphi }^{2} & \sigma^{2}{+\varphi }^{2} \end{array}\right)$$

The asymptotic GLS estimates of a k x 1 vector of parameters $\hat{\beta }$ are given by:

$$\hat{\beta }={E(X^{T}\Omega^{-1}X)}^{-1}E(X^{T}\Omega^{-1}Y)$$

where **X** is a 2 x *k* matrix consisting of the genotypes of the offspring and/or the imputed or genotyped parents of offspring one and two (where *k* is the number of regressors), and **Y** is a 2 x 1 matrix of offspring phenotypes [28].

It is then possible to derive the residual covariance matrix (**Σ**), and subsequently the expected minus two log-likelihood (-2lnL) of the model per relative pair [29]:

E(−2lnL)=ln|Σ|+2

The non-centrality parameter (*ζ*) for a test of the difference in fit between a model and a nested sub model is equal to the difference in expected minus two log-likelihoods between the models:

$$\zeta =E(-2lnL_{N})-E(-2lnL_{F})$$

where E(−2lnL_F_) and E(−2lnL_N_) represent the expected minus two log-likelihoods under the full and nested models respectively.

In order to illustrate calculation of the residual covariance matrix, the expected minus two log-likelihood (and consequently the non-centrality parameters), we consider the following models in the case of sibling pairs at autosomal loci (additional derivations of these quantities for sibling and half sibling pairs at autosomal and X chromosome loci for all models and nested sub-models are provided in the S1 Text):

Null model of no association in sibling pairs

The residual covariance matrix is simply the covariance matrix of *Y*:

$$\Sigma =\Sigma_{Y}$$

$$=\left(\begin{array}{cc} 1 & 0.5b^{2}+d^{2}+f^{2}+bd+bf+\varphi^{2}\\ 0.5b^{2}+d^{2}+f^{2}+bd+bf+\varphi^{2} & 1 \end{array}\right)$$

The expected minus two log-likelihood (-2lnL) of the model per sibling pair is therefore:

$$E(-2lnL)=ln({1-(0.5b^{2}+d^{2}+f^{2}+bd+bf+\varphi^{2})}^{2})+2$$

Full Omnibus Model in sibling pairs (terms for *X*_*m*_, *X*_*f*_, *X*_1_ and *X*_2_:

In the case of sibling pairs with genotyped parents, the **X** matrix contains three columns; column 1 with elements *X*_1_ and *X*_2_, column 2 with elements *X*_*m*_ and *X*_*m*_, and column 3 with elements *X*_*f*_ and *X*_*f*_. The asymptotic GLS estimate of the regression coefficients of columns 1, 2 and 3 are:

$$\hat{g}={\left(\hat{b},\hat{d,}\hat{f}\right)}^{T}=E{\left(X^{T}\Omega^{-1}X\right)}^{-1}E(X^{T}\Omega^{-1}Y)$$

$$={\left(b,d,f\right)}^{T}$$

The residual covariance matrix is:

$$\Sigma =E(Y-X\hat{g}){\left(Y-X\hat{g}\right)}^{T}$$

=Ω

$$=\left(\begin{array}{cc} \sigma^{2}{+\varphi }^{2} & \varphi^{2}\\ \varphi^{2} & \sigma^{2}{+\varphi }^{2} \end{array}\right)$$

Full omnibus model in sibling pairs with imputed parental genotypes (terms for $X_{p}^{\prime },X_{1}\;and\;X_{2}$)

When only imputed parental genotypes are available, the linear mixed model for sibling pairs becomes:

$$Y_{1i}=bX_{1i}+cX_{p,i}^{\prime }+\tau_{i}+\varepsilon_{1i}$$

$$Y_{2i}=bX_{2i}+cX_{p,i}^{\prime }+\tau_{i}+\varepsilon_{2i}$$

The **X** matrix contains two columns; column 1 with elements *X*_1_ and *X*_2_, and column 2 with elements $X_{p}^{\prime }$ and $X_{p}^{\prime }$. The asymptotic GLS estimate of the regression coefficients of columns 1 and 2 are:

$${\hat{g}=(\hat{b},\hat{c})}^{T}=E{\left(X^{T}\Omega^{-1}X\right)}^{-1}E(X^{T}\Omega^{-1}Y)$$

$$={\left(b,c\right)}^{T}$$

The residual covariance matrix is:

$$\Sigma =E(Y-X\hat{g}){\left(Y-X\hat{g}\right)}^{T}$$

$$=\Sigma_{Y}-E(X\hat{g}Y^{T}+Y{\hat{g}}^{T}X^{T}-X\hat{g}{\hat{g}}^{T}X^{T})$$

where:

$$E(X\hat{g}Y^{T})=E(Y{\hat{g}}^{T}X^{T})=\left(\begin{array}{cc} \hat{b}\times Cov(X_{1},Y_{1})+\hat{c}\times Cov(X_{p}^{\prime },Y_{1}) & \hat{b}\times Cov(X_{2},Y_{1})+\hat{c}\times Cov(X_{p}^{\prime },Y_{1})\\ \hat{b}\times Cov(X_{1},Y_{2})+\hat{c}\times Cov(X_{p}^{\prime },Y_{2}) & \hat{b}\times Cov(X_{2},Y_{2})+\hat{c}\times Cov(X_{p}^{\prime },Y_{2}) \end{array}\right)$$

and:

$$E(X\hat{g}{\hat{g}}^{T}X^{T})=\left(\begin{array}{cc} {\hat{b}}^{2}+\hat{b}\hat{c}+{\hat{c}}^{2}\times Var(X_{p}^{\prime }) & {0.5\hat{b}}^{2}+\hat{b}\hat{c}+{\hat{c}}^{2}\times Var(X_{p}^{\prime })\\ {0.5\hat{b}}^{2}+\hat{b}\hat{c}+{\hat{c}}^{2}\times Var(X_{p}^{\prime }) & {\hat{b}}^{2}+\hat{b}\hat{c}+{\hat{c}}^{2}\times Var(X_{p}^{\prime }) \end{array}\right)$$

### Comparison of simulated and asymptotic results

A summary of the results of our data simulations is presented in S1 Fig. Estimates of paternal, maternal and offspring genetic effects from the full omnibus models were unbiased, even when imputed parental genotype dosages were used in the place of real genotypes. Type 1 error rates were also maintained at expected levels (S6 Table, S7 Table, S8 Table, S9 Table, S10 Table). Estimates of statistical power, closely matched those from asymptotic calculations (S6 Table, S7 Table, S8 Table, S9 Table, S10 Table and see below).

### Results of asymptotic power calculations

We used our asymptotic formulae to investigate the statistical power to detect association across a range of different parameters, study designs and statistical tests (S11 Table, S12 Table, S13 Table, S14 Table, S15 Table). We highlight some general results from our power calculations that we hope investigators may find useful in terms of planning genetic association studies, particularly those aimed at identifying and/or estimating the contribution of indirect parental genetic effects on offspring phenotypes.

**Fig 3:**
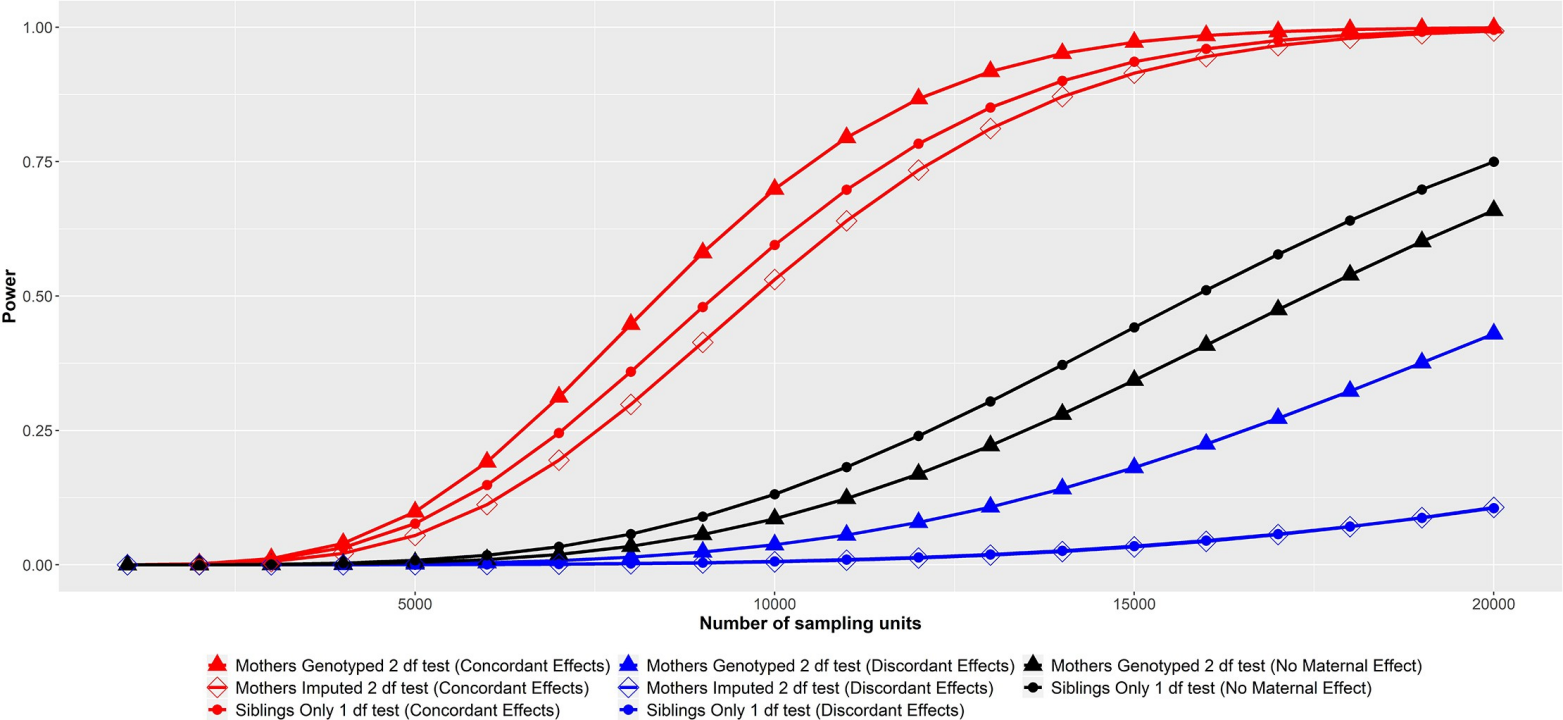
Power of locus detection in sibling pairs assuming directionally concordant maternal and offspring genetic effects (red lines: *d*^*2*^ = 0.1%; *b*^*2*^ = 0.1%), directionally discordant maternal and offspring genetic effects (blue lines: *d*^*2*^ = 0.1%; *b*^*2*^ = 0.1%), or offspring genetic effects only (black lines: *d*^*2*^ = 0%; *b*^*2*^ = 0.1%). Shown are results of a one degree of freedom test using sibling genotypes only (lines with circles), an omnibus two degree of freedom test using observed genotypes in siblings and their mothers (lines with triangles), and an omnibus two degree of freedom test of association when parental genotypes need to be imputed from sibling genotypes (lines with open boxes). For all calculations we assume an autosomal locus, shared residual variance *φ*^2^ = 0.2, a type 1 error rate α = 5x10^-8^, and where relevant, a decreasing allele frequency of p = 0.1. The graph shows that when observed genotypes in mothers are available, power to detect loci may be greatest when employing a two degree of freedom test, providing maternal effects are present, and particularly when maternal and offspring genetic effects are directionally discordant. In contrast, when maternal effects are absent, simply fitting a one degree of freedom model using sibling genotypes alone is often the best strategy. When parental genotypes are unavailable, there appears to be little gained from imputing genotypes in mothers in terms of power to detect loci. Note that power is similar for two conditions shown in this graph (i.e. in the case of discordant maternal and offspring genetic effects for the Siblings only one degree of freedom test and the two degrees of freedom test when mothers have to be imputed). For simplicity, we do not show results for the two degree of freedom test when mothers are imputed and there is no maternal effect (i.e. this condition has identical power to when mothers are genotyped).

A key question for researchers is, what is the optimal analysis strategy if the primary focus is on locus detection? According to our power calculations, the answer to this question, perhaps unsurprisingly, depends on the genetic architecture of the trait, in particular on the existence of indirect maternal/paternal genetic effects and whether these are in the same or opposing directions. Fig 3 displays power to detect a locus using sibling pairs when a locus is influenced by maternal and/or offspring genetic effects (e.g. a perinatal trait like birth weight). When the locus under study involves an offspring genetic effect only (black lines in Fig 3), which is probably the case for the majority of loci in the genome for most traits, then the most powerful strategy appears to be simply testing for an offspring genetic effect against the null model of no association (i.e. performing a one degree of freedom test just using the sibling pairs with no parental imputation). This includes situations where parents have been genotyped. This is because fitting the full omnibus model and testing against the null model requires extra degrees of freedom to model parental genetic effects (which in this case are not present) which adversely affects power. We note that this decrement does not appear to be great in the case of sibling pairs if only the mother is genotyped and paternal genetic effects are not modelled and do not contribute to the trait of interest, (Fig 3, S11 Table)- which is perhaps a reasonable assumption for many perinatal phenotypes.

In contrast, when indirect maternal (or paternal) genetic effects substantially influence the offspring phenotype (blue and red lines Fig 3), and parental genotypes are present, the full omnibus model (lines with triangles) often performs comparably or better than a simple one degree of freedom test using the sibling genotypes alone (lines with small circles). This is especially the case when offspring and/or parental genetic effects are directionally discordant (blue lines), as is frequently observed for some trait-locus combinations like fasting glucose associated loci and offspring birth weight [10,11]. Here the power of a simple one degree of freedom test involving the sibling genotypes only can be vastly diminished, because the discordant parental and offspring genetic effects tend to cancel each other out. In contrast, an omnibus test which models both offspring and indirect parental genetic effects performs much better in these situations. Importantly, when parental genotypes are unavailable, for many situations there appears to be little gained (and in some cases power is lost) by imputing parental genotypes and including these in an omnibus test if the focus is solely on locus detection (Fig 3; S11 Table, S12 Table).

**Fig 4:**
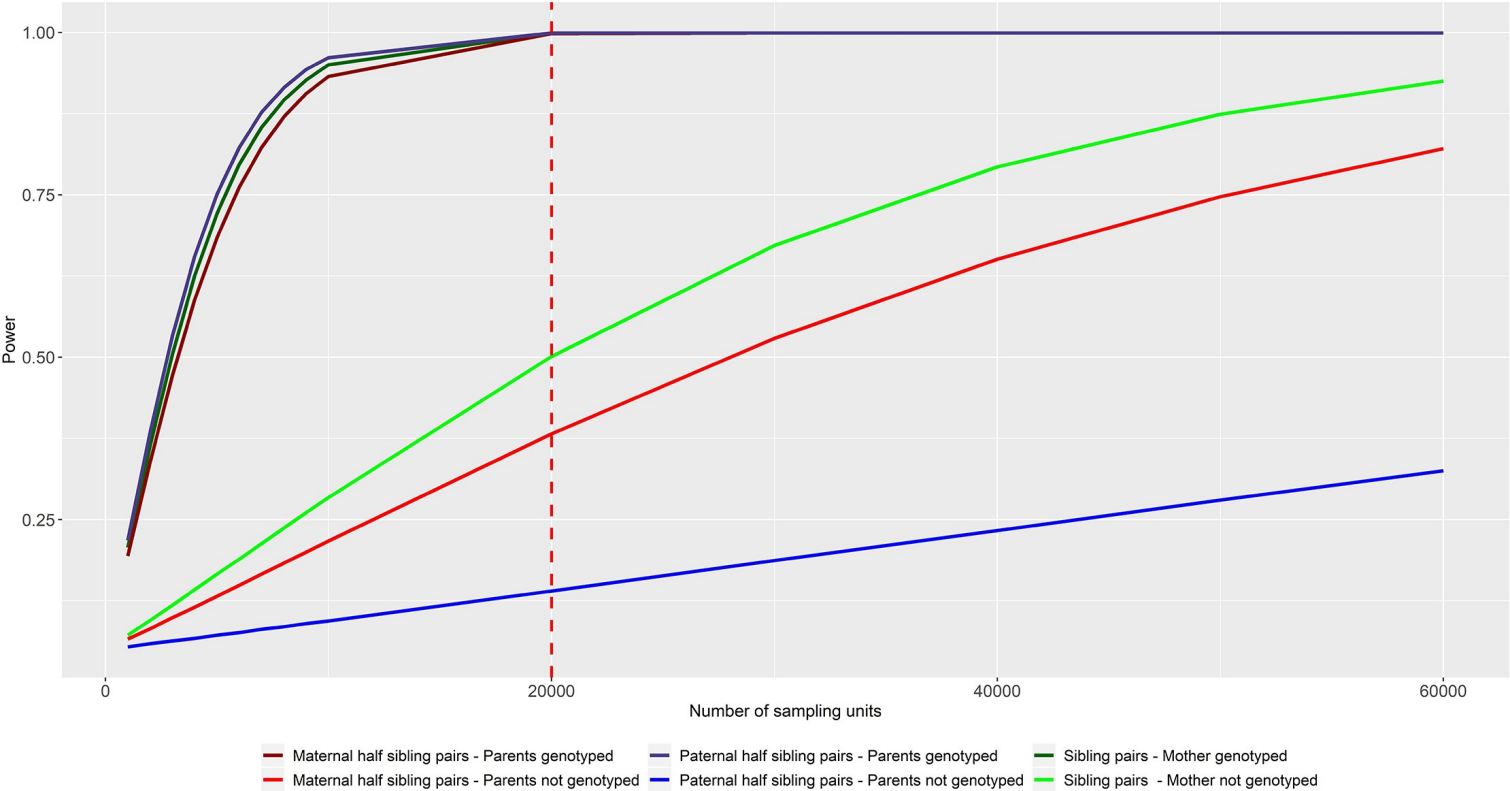
Power to resolve an autosomal maternal genetic effect (*d*^*2*^ = 0.1%; *f* ^*2*^ = 0%; *b*^*2*^ = 0%;) at a known genetic locus, using a conditional one degree of freedom test of association in sibling pairs (green lines), maternal half sibling pairs who share the same mother (red lines) and paternal half sibling pairs who share the same father (blue lines). All calculations assume p = 0.3 frequency of the trait decreasing allele; shared variance *φ*^2^ = 0.2; type 1 error rate α = 0.05). The red dashed vertical line in the figure indicates the approximate number of sibling pairs in the UK Biobank (N = 20,000). This figure highlights the advantage of having actual parental genotypes in the statistical model.

Another goal investigators might be interested in is partitioning effects at known genetic loci into direct offspring and indirect parental genetic components. This may be of relevance if investigators want to prove the existence of indirect maternal genetic effects on offspring phenotypes for example. Fig 4 displays the power to partition a genetic effect into maternal (or equivalently paternal) genetic sources of variation in the case of half sibling or sibling pairs, with and without parental genotypes at autosomal loci. The graph highlights the clear advantage in power of including actual as opposed to imputed parental genotypes in the statistical model when the focus is on resolving indirect parental genetic effects on offspring phenotypes. Fig 4 shows that if parental genotypes are unavailable, then a considerable number of sibling pairs (>40,000) and maternal half sibling pairs (>60,000) will be required to achieve high power (>80%, α = 0.05) to partition genetic effects at a locus- even for those of relatively large effect (*d*^2^ = 0.1%). Interestingly, paternal half sibling pairs who have not had their parents genotyped, provide much less power to estimate maternal genetic effects and require even larger numbers (a similar decrement in power is also observed in the case for maternal half sibling pairs if the interest is in estimating paternal genetic effects). The lower power of the imputed half sibling analyses compared to the imputed sibling analyses partially reflects the fact that only two sources of variation are modelled in the imputed sibling models (i.e. offspring and parental genetic sources of variation), whereas in the half sibling models, three different sources of variation are modelled (offspring, parental, maternal genetic sources of variation). If investigators believe that paternal genetic effects do not contribute to offspring trait variation (a reasonable assumption for perinatal traits), then one option to increase power is to fix this path to zero in models involving half siblings. Interestingly, the presence/absence of other genetic effects has little effect on power of the conditional tests of association for realistic effect sizes when correctly modelled.

In order to put the above numbers in context, in the publicly available UK Biobank dataset (which roughly includes ~20,000 sibling pairs), we estimate that an autosomal parental genotype would need to explain ~0.2% of the variance in the offspring phenotype in order to have 80% power to resolve an indirect parental genetic effect if parental genotypes need to be imputed (assuming the same parameters as in Fig 4). An indirect effect size this large is probably unrealistic for most traits, implying that larger samples will be needed to resolve genetic effects at known loci into indirect and direct genetic effects if parental genotypes need to be imputed. We also note that the power of the conditional tests is typically lower than omnibus tests, implying that omnibus tests of association should be used for locus discovery purposes whilst conditional tests of association should be reserved for partitioning effects/estimating effect sizes at known loci (S11 Table, S12 Table, S13 Table, S14 Table, S15 Table).

We found that the effect of the other parameters we investigated (allele frequency, shared residual variance) on statistical power was complicated and often interacted with the level of other factors in the calculation (S11 Table, S12 Table, S13 Table, S14 Table, S15 Table). Allele frequency exerted a modest effect on the power of most of the statistical tests examined, and its effect on power appeared to be symmetric around p = 0.5. The effect of the shared residual variance on statistical power was complex and depended on the statistical test, the underlying genetic model, allele frequency etc (S11 Table, S12 Table, S13 Table, S14 Table, S15 Table).

Imputing parental genotypes on the X chromosome has the advantage that separate maternal, paternal and offspring genetic effects can be resolved for sibling pairs (although at X linked loci, male siblings are uninformative for paternal transmissions, and so contribute nothing in terms of identifying paternal genetic effects when fathers have not been genotyped). We parameterize the statistical model at X linked loci so that unstandardized male genotypes are coded *G*_*1*_ ∈ {0, 2} and female genotypes are coded *G*_*2*_ ∈ {0, 1, 2}. We also assume that the regression coefficient of offspring phenotype on (mother’s/father’s/offspring’s) genotype is the same in male and female offspring. This means that male loci explain double the amount of variance in the phenotype compared to females (see S1 Text). We have coded the web utilities (http://evansgroup.di.uq.edu.au/power-calculators.html) so that users enter the variance in the offspring phenotype explained by mother’s, father’s and/or offspring genotypes at the locus. For offspring genetic effects in opposite sex siblings, users enter the variance explained by male loci.

The results of the power analyses for sibling pairs on the X chromosome are displayed in S13 Table, S14 Table, and S15 Table. The general pattern of results for loci on the X chromosome was similar to that described for the autosomes, and consequently we make similar recommendations regarding the appropriate analyses for locus detection and partitioning genetic effects at X linked loci. Comparing the power across the different study designs however revealed a few interesting results which we highlight. First, male sibling pairs offer increased power to resolve indirect paternal genetic effects on the X chromosome compared to the autosomes- so long as fathers have been genotyped. This is because (under random mating) father’s genotype is uncorrelated with mother’s and male offspring genotype at X linked loci. Correlation between genotypes at X linked loci in fathers and the phenotypes of their male offspring can therefore not be explained by mother’s or offspring’s genotype. The corollary is that male sibling pairs cannot be used to impute the genotypes of their fathers at X linked loci and so are uninformative for paternal genetic effects unless the father has been physically genotyped (opposite sex sibling pairs also provide slightly elevated power to detect paternal genetic effects when fathers have been genotyped for the same reason).

When parental genotypes are present, male siblings also provide lower power to detect X linked loci (3 degree of freedom tests) compared to many of the other study designs. The reason is the converse of the explanation above- paternal genetic effects do not contribute to the covariance between male sibling pairs. Opposite sex sibling pairs also provide reduced power to detect loci (3 degree of freedom tests), but this partly a consequence of how we parameterize the model of association on the X chromosome (i.e. we calculate the size of offspring genetic effects in reference to the variance explained by male offspring, meaning that the variance explained by the same locus in the sister will be half this amount). We choose not to compare results across the different study designs when parental genotypes are imputed because the different models and their tests are usually not equivalent (e.g. one can’t resolve paternal genotypes at X linked loci for male sibling pairs; opposite sex sibling pairs have their variances parameterized slightly differently to the other sibships etc). These results are tabulated in S13 Table, S14 Table, and S15 Table.

### Results of simulations investigating differential missing rates

S16 Table displays the results of simulations investigating the effect of differential missing rates on our method. In general, our simulations show that under some models, (strong) differential missing rates can produce modest biases in parameter estimates and inaccurate asymptotic power calculations. The exact effect depends on allele frequency in the base population, the residual correlation between the offspring pair, and the strength and type of process generating the missing data. Type 1 error rates, however, appeared to be appropriate under all the conditions we simulated.

### Analysis of educational attainment in the UK biobank

We found that own educational attainment polygenic risk score and imputed parental polygenic risk score were strongly related to own educational attainment (Table 1). Interestingly, in these analyses imputed parental genotypic risk score showed a stronger relationship with educational attainment than own genotypic risk score. This may reflect the fact that parental dosage represents the combined effect of mother’s and father’s genotypes on offspring educational attainment. Sensitivity analyses using polygenic risk scores constructed from 72 SNPs from the Okbay et al (2016) GWAS yielded similar results. Analysis of parent-offspring duos and trios also suggested that genetic risk scores in mothers and fathers also affected offspring educational attainment in addition to offspring’s own genotype.

**Table 1:** UK Biobank Results. Results of own educational attainment regressed on (A) own genotyped polygenic risk score (PRS) and imputed parental PRS in sibling pairs, (B) own genotyped PRS and genotyped maternal PRS in mother-offspring duos, (C) own genotyped PRS and genotyped paternal PRS in father-offspring duos, and (D) own genotyped PRS, genotyped maternal PRS and genotyped paternal PRS in parent-offspring trios. All analyses were corrected for sex, year of birth and the first five principal components (PC) from the UK Biobank GWAS data. Sex was coded as 1 and 0 for males and females respectively. PRS were constructed using 1264 SNPs associated with education attainment identified in Lee et al. (2018). A sensitivity analysis was performed for (A) using PRS constructed using 72 SNPs identified in Okbay et al. (2016).

		Estimate	Standard Error	p-value
**(A) Sibling pairs** (n = 19066)	Intercept	-77.003	2.295	4.22x10^-240^
	Own PRS	0.007	0.001	2.38x10^-46^
	Imputed Parental PRS	0.014	0.001	1.00x10^-49^
	Sex	0.186	0.014	8.15x10^-38^
	Birth Year	0.027	0.001	8.76x10^-131^
	PC 1	-0.007	0.004	0.119
	PC 2	-0.006	0.005	0.250
	PC 3	0.004	0.005	0.427
	PC 4	-0.015	0.003	4.15x10^-6^
	PC 5	0.005	0.001	5.75x10^-4^
Sensitivity analysis	Intercept	-49.501	2.210	9.76x10^-110^
	Own PRS	0.003	0.002	0.060
	Imputed Parental PRS	0.023	0.003	1.92x10^-11^
	Sex	0.187	0.015	8.84x10^-37^
	Birth Year	0.025	0.001	5.03x10^-112^
	PC 1	-0.010	0.005	0.027
	PC 2	-0.007	0.005	0.148
	PC 3	0.004	0.005	0.422
	PC 4	-0.018	0.003	9.71x10^-8^
	PC 5	0.007	0.001	1.62x10^-6^
**(B) Mother-offspring duos** (n = 4071)	Intercept	-116.600	17.470	2.89x10^-11^
	Own PRS	0.009	0.001	8.40x10^-19^
	Maternal PRS	0.004	0.001	3.69x10^-4^
	Sex	-0.048	0.045	0.287
	Birth Year	0.052	0.009	6.49x10^-9^
	PC 1	0.000	0.013	0.983
	PC 2	-0.010	0.013	0.427
	PC 3	-0.001	0.014	0.953
	PC 4	-0.009	0.009	0.346
	PC 5	0.007	0.004	0.091
**(C) Father-offspring duos** (n = 1809)	Intercept	-128.800	29.650	1.48x10^-5^
	Own PRS	0.009	0.002	4.09x10^-8^
	Paternal PRS	0.003	0.002	0.058
	Sex	-0.031	0.065	0.638
	Birth Year	0.059	0.015	1.05x10^-4^
	PC 1	-0.017	0.018	0.338
	PC 2	-0.017	0.019	0.382
	PC 3	-0.025	0.020	0.200
	PC 4	-0.029	0.013	0.027
	PC 5	0.023	0.006	5.63x10^-5^
**(D) Parent-offspring trios** (n = 1064)	Intercept	-146.300	41.300	4.17x10^-4^
	Own PRS	0.006	0.003	0.024
	Maternal PRS	0.004	0.002	0.054
	Paternal PRS	0.003	0.002	0.169
	Sex	-0.097	0.087	0.265
	Birth Year	0.067	0.021	0.002
	PC 1	-0.024	0.026	0.365
	PC 2	-0.051	0.027	0.060
	PC 3	-0.028	0.027	0.312
	PC 4	-0.041	0.019	0.033
	PC 5	0.027	0.008	0.001

### IMPISH software performance

S17 Table shows the performance of the **IMPISH** software in terms of CPU times and time to perform genome-wide association. Our results show that **IMPISH** can be used to impute parental genotypes from large numbers of relative pairs and perform tests of association across the genome in a reasonable time frame. Note that IMPISH allows multi-threading so computation time will decrease even further when running the software on multiple threads.

## Discussion

In this manuscript we have shown that it is possible to impute parental genotypes given genotype data on sibling and half sibling pairs and then subsequently use this information to derive unbiased estimates of parental genetic effects. We are not the first to have developed methods for estimating genetic parameters in sibling pairs when parental genotype data is missing [30–32], nor are we the first to propose a method for estimating maternal genetic effects in the absence of genotype data from one or more parents. Weinberg and colleagues introduced a log-linear model for the analysis of case-parent trio data that could be used to test for the presence of maternal genetic effects on binary outcomes [5,6]. They subsequently showed how the Expectation Maximization algorithm could be used within the same log-linear framework to incorporate families into the analysis where genotype data from one or both parents were missing [33] or when genotypes from unaffected siblings were available [34]. Cordell and colleagues showed how parent-case trios and other family structures that included missing parents could be fitted in a coherent multinomial framework to test for maternal genetic effects [7] and also provided power calculations [35] and software to do so [36]. Our work builds upon these previous approaches, however, our approach differs in that the focus is on testing for indirect genetic effects (i.e. both maternal and paternal) in the case of quantitative traits (as opposed to binary affection status) when parental genotype data is not available (or only partially available), and we do so via genotype imputation. We also specifically consider the case of sibling and half sibling pairs (as opposed to case-parent triads, case and control-mother duos etc), and X chromosome as well as autosomal loci.

Our asymptotic calculations reveal that the power to partition known individual loci into parental and offspring genetic effects using imputed parental genotypes is low in general, and highlight the value in having parents genotyped if the interest is in resolving indirect parental genetic effects at known loci. In situations where parental genotypes are unavailable, we show that indirect parental genetic effects can still be estimated without bias, but very large numbers of sibling (or half sibling) pairs will be required (e.g. >40,000 sibling and >60,000 half sibling pairs). Whilst these sorts of numbers may be realistic in the case of siblings (e.g. UK biobank contains roughly 20,000 sibling pairs, and there are many twin cohorts around the world that contain large numbers of dizygotic twins), most cohorts contain very few half sibling pairs. For these reasons we suggest our method may currently be more suitable as a complement to existing large-scale genetic studies of parents and their children. For example, both the Norwegian MOBA and HUNT cohorts not only contain tens of thousands of parent-offspring trios and duos, but also large numbers of sibling pairs that could be combined with more traditional parent-offspring analyses to further increase power to detect parental genetic effects [19,20].

A key motivation for developing our approach was the realization that estimates of parental genetic effects derived from imputed genotypes could also be used in two sample Mendelian randomization (MR) studies examining possible causal relationships between parental exposures and offspring outcomes [2]. Whilst our method could be used to increase the power of existing MR analyses involving perinatal outcomes [9], an exciting novel application would be the examination of the influence of parental exposures on later life offspring outcomes. The majority of the world’s large-scale cohorts with genotyped mother-offspring pairs are relatively new historically [17,18,20,37]. This means that the children in these cohorts are not old enough to have developed many late onset diseases of interest including adverse cardiometabolic phenotypes. Consequently, it is currently difficult, if not impossible, to perform maternal-offspring MR studies on late onset diseases. Our procedure of imputing parental genotypes means that in principle mother-offspring MR analyses are now possible utilizing cohorts of mature sibling and half sibling pairs. Such an approach would enable the investigation of hypotheses in life course epidemiology such as the Developmental Origins of Health and Disease which posits a link between intrauterine growth restriction and the development of disease in the offspring in later life [38].

Besides low statistical power, there are a number of limitations with our approach. In the case of sibling pairs, different genotypes for mothers and fathers can be resolved at X linked (non-pseudoautosomal) loci. However, for autosomal loci, the expected dosage for parental genotypes is the same. This means that it is impossible to distinguish different genotypes for mothers and fathers using data from sibling pairs alone. Thus, utilization of sibling pairs to detect indirect genetic effects requires the non-trivial assumption that either paternal (or maternal) genetic effects do not affect the offspring phenotype under study. Whilst this assumption may be justified for certain perinatal phenotypes where the contribution of the father’s phenotype to trait variation in the offspring may be minimal (like birth weight), it may not be justifiable for other phenotypes. Sensitivity analyses could be performed by testing whether estimates derived from using sibling pairs are consistent with those derived from e.g. parent-offspring trios or even half sibling pairs where estimates of maternal, paternal and offspring genetic effects can be estimated consistently.

We have shown that for half sibling pairs, different genotype probabilities for mothers and fathers (and therefore expected dosages) can be resolved at genetic loci. This means that, in principle, the half sibling pairs within large publicly available biobanks could be leveraged to provide information on parental genotypes and consequently help obtain unbiased estimates of indirect parental genetic effects on offspring traits. This will be possible if there is explicit pedigree information that unequivocally identifies half sibling relationships. However, the task becomes more challenging if half siblings have to be identified on the basis of genetic information alone. This is because half siblings share the same expected number of alleles identical by descent as grandparent-grandchild pairs and avuncular relationships, making it difficult to distinguish between these relationships given only genetic data. The majority of grandparent-grandchild pairs can be differentiated from half sibling pairs on the basis of age (i.e. the age difference in most grandparent-grandchild relationships will be >30 years). However, it is much more difficult to resolve half sibling from avuncular pairs. Half siblings and avuncular pairs can be partially distinguished by the former’s longer haplotype sharing. Intuitively, this is because any chromosome segments that half siblings share have only gone through a total of two meioses since their common ancestor (i.e. transmission from the shared parent to half sibling one and transmission from the shared parent to half sibling two). In contrast, any shared haplotype segments have gone through a total of three meioses since the last common ancestor in the case of avuncular relationships (i.e. transmission from shared grandparent to uncle/aunt and transmission from shared grandparent to parent to child). However, classification is imperfect [39–41], but could be improved further through the use of additional information including age difference of the pair and reported information on the parents (e.g. half siblings who share the same mother should produce consistent reports of maternal illnesses). Any half sibling pairs that are identified would need to be classified into maternal half siblings (who share a mother) and paternal half siblings (who share a father). Genetic data on the sex chromosomes and mitochondria could help facilitate this differentiation.

Our approach of imputing parental genotypes and utilizing them in downstream analyses assumes that individual loci are in Hardy-Weinberg equilibrium and that parents mate randomly with respect to the locus under consideration. Therefore, in theory, any process that leads to deviations from Hardy-Weinberg equilibrium and/or random mating could affect the accuracy of our imputation and consequently asymptotic power calculations, downstream type 1 error rates and parameter estimates. This includes processes producing missing data at genetic loci (i.e. genotypes at the locus under consideration being related to missingness or selection into or out of the study), population stratification and non-random mating.

Our simulations show that our imputation procedure can produce moderately biased estimates of parental and offspring genetic effects if parental genotype is strongly related to missingness and deviations from asymptotic power under complete data (although type 1 error rate was unaffected for the scenarios we considered). However, the vast majority of loci across the genome are not expected to exhibit strong differential missing rates, and so for most genetic markers this possibility is unlikely to be a major concern. Those loci that do exhibit strong associations with missingness may also show departures from Hardy-Weinberg equilibrium and therefore we recommend that users of our approach test markers for departures from Hardy-Weinberg equilibrium as a matter of routine. Our approach should not be used for markers that exhibit strong departures from Hardy-Weinberg equilibrium, and only used with caution at loci that are expected to show strong relationships with missing rates (e.g. *APOE* alleles in studies of elderly individuals).

The presence of latent population substructure means that allele frequencies and hence parental genotype imputation will be less accurate at loci where differences exist in allele frequency across different sub-populations, and also that spurious association may exist between imputed parental genotypes, offspring genotypes and the offspring trait of interest. We therefore recommend that parental imputation only be performed in ancestrally homogenous samples. If a sample contains individuals from a range of different ancestries, then we recommend that parental imputation be performed in the subsamples separately if possible. Once parental genotypes have been imputed in the different subsamples, IMPISH includes facility for including ancestry informative principal components as fixed effects, and models the relatedness between individuals in the random effects part of the model via genetic relationship matrix, and so can model population structure in downstream analyses. The full effects of population stratification on our method will need to be investigated in future work and until then the method should only be used with caution in ancestrally heterogeneous samples.

Our method assumes random mating between spouses. Inbreeding produces severe departures from Hardy-Weinberg equilibrium and correlations between maternal and paternal genotypes across the entire genome, and so our method may not be appropriate in cohorts showing high levels of consanguinity. Likewise, it is well known that spouses positively assort for many traits of interest [42]. Positive assortment implies that the genotypes of spouses at loci underlying the trait on which the assortment is based (and those in linkage disequilibrium with them) will be correlated rather than random as our procedure assumes. Future work is therefore required to fully quantify the impact of assortative mating on our method.

For our asymptotic power calculations, we assume that the different sibling/half sibling pairs contributing to the estimates are unrelated. Users are warned that cryptic relatedness in samples will typically decrease power to detect association (i.e. a sample with cryptically related sibling/half sibling pairs will have reduced power compared to a similar sized sample of unrelated relative pairs when both samples are analysed using appropriate methods). That being said, our software package IMPISH models cryptic and known relatedness between individuals in the random effects part of the model ensuring that standard errors for tests of association in empirical data are appropriately computed.

Finally, we note that our analytical power calculations assume that trait errors are normally distributed. Thus, our power calculations may not be accurate in the case of data that are grossly non-normal and in these situations investigators may need to derive power estimates using simulation if an appropriate phenotype transformation is not possible.

There are several ways that our procedure could be improved/extended. First, we have only considered relative pairs in our derivations. Additional first degree relatives (i.e. additional siblings, the addition of one parent etc) would enable better genotype imputation and therefore increased power to detect parental genetic effects on offspring phenotypes. It is also possible that more distant relatives may also be informative for imputation, particularly if shared haplotypes could be identified within larger pedigree structures. Second, we have developed methods that are appropriate for the analysis of unselected normally distributed quantitative traits. Further work is required to generalize these approaches to the analysis of binary traits and selected samples. Third, we have only considered one SNP at a time. It is possible that the inclusion of haplotype information may increase imputation fidelity. Fourth we note that it is likely that family dynamics will alter the strength of indirect parental genetic effects depending on the relationship of offspring to their parents. For example, the relationship between half siblings and their birth parents is likely to be qualitatively different to those of full siblings in nuclear families. Thus, for later-life phenotypes especially, parental genetic effect size estimates in half siblings may not be comparable to those estimated from full siblings. This may be perhaps less of an issue for maternal genetic effects on perinatal phenotypes. Fifth we note that there are other ways to parameterize models of association on the X chromosome [43], and it would be possible to perform simulations and asymptotic power calculations to investigate the power of these tests of association similar to what we have done here. Sixth, it would be interesting to investigate whether imputed parental genotypes could be included in genome-wide variance component models that aim to estimate indirect parental genetic effects on offspring phenotypes [44]. Whilst we have shown that power to detect parental genetic effects at individual genetic loci may be low, the inclusion of imputed parental genotypes may help estimate the combined contribution of indirect genetic effects simultaneously across the genome. Likewise, the construction of genetic risk scores comprising imputed parental genotypes across several loci may also improve power to detect indirect parental genetic effects on offspring phenotypes. Finally, we note that the models that we have considered in this manuscript could be extended in a variety of ways including adding more relatives to help estimate sibling and/or parent of origin effects.

In conclusion, we have developed a suite of online genetic power calculators and software to assist researchers in detecting and partitioning loci that exhibit indirect parental genetic effects. We hope that our methods and utilities will form useful adjuncts to large ongoing genetic studies of parents and their offspring.

## Supporting information

S1 Text (DOCX)

S1 Fig Comparison of maternal (d), paternal (f) and offspring (b) genetic effects estimated using genotyped parental genotypes versus imputed parental genotypes in simulations. We simulated genetic effects accounting for 0.1% of the variance in the offspring trait (*b*^*2*^ = *d*^*2*^ = *f*^*2*^ = 0.1%), a trait decreasing allele frequency of *p* = 0.1, and shared residual variance of *φ*^2^ = 0.2. For all simulations we used N = 2000 sibling pairs/half sibling pairs, and 1000 replications. In the case of autosomal loci, red lines indicate the expected beta coefficients for parental (c) and offspring genetic effects (b) in full sibling pairs, and the expected beta coefficients for maternal (d), paternal (f), and offspring genetic effects (b) in half sibling pairs. Blue lines indicate the expected beta coefficients for paternal (f) and male fetal (b) effects at X chromosomal loci. Green lines indicate the expected beta coefficients for maternal (d) and female fetal (b) effect at X chromosomal loci. For opposite sex sibling pairs at X chromosomal loci, we simulated the fetal effect (b) to be the same for both siblings assuming using male genotypes. The full omnibus model was simulated and fitted in all simulations. R codes implementing the simulations are provided in the S1 Text.
(PNG)

S1 Table Probabilities (P) and expected dosages for imputed parental genotypes conditional on observed sibling pair genotypes at autosomal loci. (DOCX)

S2 Table Probabilities (P) and expected dosages for imputed parental genotypes conditional on observed sibling pair genotypes at non-pseudosomal X chromosome loci. (DOCX)

S3 Table Genotype probabilities (P) and expected dosages for imputed genotypes of shared parent conditional on observed half sibling pair genotypes at autosomal loci. (DOCX)

S4 Table Genotype probabilities (P) and expected dosages of imputed genotypes for the father of the first maternal half sibling and the father of the second maternal half sibling conditional on observed maternal half sibling genotypes at autosomal loci. (DOCX)

S5 Table SNPs used in constructing a polygenic score for educational attainment. (XLSX)

S6 Table Power (a -d) and type 1 error rate (e—h) for tests involving sibling pairs on autosomes. Results of simulations compared to asymptotic calculations (α = 0.05).
(XLSX)

S7 Table Power (a -d) and type 1 error rate (e—h) for tests involving half sibling pairs on autosomes. Results of simulations compared to asymptotic calculations (α = 0.05).
(XLSX)

S8 Table Power (a -d) and type 1 error rate (e—h) for tests involving male sibling pairs on the X chromosome. Results of simulations compared to asymptotic calculations (α = 0.05).
(XLSX)

S9 Table Power (a -d) and type 1 error rate (e—h) for tests involving female sibling pairs on the X chromosome. Results of simulations compared to asymptotic calculations (α = 0.05).
(XLSX)

S10 Table Power (a -d) and type 1 error rate (e—h) for tests involving opposite sex sibling pairs on the X chromosome. Results of simulations compared to asymptotic calculations (α = 0.05).
(XLSX)

S11 Table Power to detect association at autosomal loci using genotyped sibling pairs with and without parental genotypes (α = 0.05). (XLSX)

S12 Table Power to detect association at autosomal loci using genotyped half sibling pairs with and without parental genotypes (α = 0.05). (XLSX)

S13 Table Power to detect association at X chromosome loci using genotyped male-male sibling pairs with and without parental genotypes (α = 0.05). (XLSX)

S14 Table Power to detect association at X chromosome loci using genotyped female-female sibling pairs with and without parental genotypes (α = 0.05). (XLSX)

S15 Table Power to detect association at X chromosome loci using genotyped opposite sex sibling pairs with and without parental genotypes (α = 0.05). (XLSX)

S16 Table Simulations investigating the effect of missing parental genotypes on bias, power and type 1 error. (XLSX)

S17 Table Computational performance of IMPISH. (DOCX)

S18 Table Marginal probability of Sibling Pair Genotypes. (DOCX)

## References

[1] Bates TC, Maher BS, Medland SE, McAloney K, Wright MJ, Hansell NK, et al The Nature of Nurture: Using a Virtual-Parent Design to Test Parenting Effects on Children's Educational Attainment in Genotyped Families. Twin Res Hum Genet. 2018;21(2):73–83. 10.1017/thg.2018.11 29530109

[2] Evans DM, Moen GH, Hwang LD, Lawlor DA, Warrington NM. Elucidating the role of maternal environmental exposures on offspring health and disease using two-sample Mendelian randomization. Int J Epidemiol. 2019;48(3):861–75. 10.1093/ije/dyz019 30815700 PMC6659380

[3] Kong A, Thorleifsson G, Frigge ML, Vilhjalmsson BJ, Young AI, Thorgeirsson TE, et al The nature of nurture: Effects of parental genotypes. Science. 2018;359(6374):424–8. 10.1126/science.aan6877 29371463

[4] Lawlor D, Richmond R, Warrington N, McMahon G, Davey Smith G, Bowden J, et al Using Mendelian randomization to determine causal effects of maternal pregnancy (intrauterine) exposures on offspring outcomes: Sources of bias and methods for assessing them. Wellcome Open Res. 2017;2:11 10.12688/wellcomeopenres.10567.1 28405635 PMC5386135

[5] Wilcox AJ, Weinberg CR, Lie RT. Distinguishing the effects of maternal and offspring genes through studies of "case-parent triads". Am J Epidemiol. 1998;148(9):893–901. 10.1093/oxfordjournals.aje.a009715 9801020

[6] Weinberg CR, Wilcox AJ, Lie RT. A log-linear approach to case-parent-triad data: assessing effects of disease genes that act either directly or through maternal effects and that may be subject to parental imprinting. Am J Hum Genet. 1998;62(4):969–78. 10.1086/301802 9529360 PMC1377041

[7] Ainsworth HF, Unwin J, Jamison DL, Cordell HJ. Investigation of maternal effects, maternal-fetal interactions and parent-of-origin effects (imprinting), using mothers and their offspring. Genet Epidemiol. 2011;35(1):19–45. 10.1002/gepi.20547 21181895 PMC3025173

[8] Beaumont RN, Warrington NM, Cavadino A, Tyrrell J, Nodzenski M, Horikoshi M, et al Genome-wide association study of offspring birth weight in 86 577 women identifies five novel loci and highlights maternal genetic effects that are independent of fetal genetics. Hum Mol Genet. 2018;27(4):742–56. 10.1093/hmg/ddx429 29309628 PMC5886200

[9] Tyrrell J, Richmond RC, Palmer TM, Feenstra B, Rangarajan J, Metrustry S, et al Genetic Evidence for Causal Relationships Between Maternal Obesity-Related Traits and Birth Weight. JAMA. 2016;315(11):1129–40. 10.1001/jama.2016.1975 26978208 PMC4811305

[10] Warrington NM, Beaumont RN, Horikoshi M, Day FR, Helgeland O, Laurin C, et al Maternal and fetal genetic effects on birth weight and their relevance to cardio-metabolic risk factors. Nat Genet. 2019;51(5):804–14. 10.1038/s41588-019-0403-1 31043758 PMC6522365

[11] Warrington NM, Freathy RM, Neale MC, Evans DM. Using structural equation modelling to jointly estimate maternal and fetal effects on birthweight in the UK Biobank. Int J Epidemiol. 2018;47(4):1229–41. 10.1093/ije/dyy015 29447406 PMC6124616

[12] Yang Q, Millard LAC, Davey Smith G. Proxy gene-by-environment Mendelian randomization study confirms a causal effect of maternal smoking on offspring birthweight, but little evidence of long-term influences on offspring health. Int J Epidemiol. 2019 10.1093/ije/dyz250 31834381 PMC7660158

[13] Zhang G, Bacelis J, Lengyel C, Teramo K, Hallman M, Helgeland O, et al Assessing the Causal Relationship of Maternal Height on Birth Size and Gestational Age at Birth: A Mendelian Randomization Analysis. PLoS Med. 2015;12(8):e1001865 10.1371/journal.pmed.1001865 26284790 PMC4540580

[14] Zhang G, Feenstra B, Bacelis J, Liu X, Muglia LM, Juodakis J, et al Genetic Associations with Gestational Duration and Spontaneous Preterm Birth. N Engl J Med. 2017;377(12):1156–67. 10.1056/NEJMoa1612665 28877031 PMC5561422

[15] Zhang G, Srivastava A, Bacelis J, Juodakis J, Jacobsson B, Muglia LJ. Genetic studies of gestational duration and preterm birth. Best Pract Res Clin Obstet Gynaecol. 2018;52:33–47. 10.1016/j.bpobgyn.2018.05.003 30007778 PMC6290110

[16] Moen GH, Hemani G, Warrington NM, Evans DM. Calculating Power to Detect Maternal and Offspring Genetic Effects in Genetic Association Studies. Behav Genet. 2019;49(3):327–39. 10.1007/s10519-018-9944-9 30600410

[17] Boyd A, Golding J, Macleod J, Lawlor DA, Fraser A, Henderson J, et al Cohort Profile: the 'children of the 90s'—the index offspring of the Avon Longitudinal Study of Parents and Children. Int J Epidemiol. 2013;42(1):111–27. 10.1093/ije/dys064 22507743 PMC3600618

[18] Fraser A, Macdonald-Wallis C, Tilling K, Boyd A, Golding J, Davey Smith G, et al Cohort Profile: the Avon Longitudinal Study of Parents and Children: ALSPAC mothers cohort. Int J Epidemiol. 2013;42(1):97–110. 10.1093/ije/dys066 22507742 PMC3600619

[19] Krokstad S, Langhammer A, Hveem K, Holmen TL, Midthjell K, Stene TR, et al Cohort Profile: the HUNT Study, Norway. Int J Epidemiol. 2013;42(4):968–77. 10.1093/ije/dys095 22879362

[20] Magnus P, Irgens LM, Haug K, Nystad W, Skjaerven R, Stoltenberg C, et al Cohort profile: the Norwegian Mother and Child Cohort Study (MoBa). Int J Epidemiol. 2006;35(5):1146–50. 10.1093/ije/dyl170 16926217

[21] Sudlow C, Gallacher J, Allen N, Beral V, Burton P, Danesh J, et al UK biobank: an open access resource for identifying the causes of a wide range of complex diseases of middle and old age. PLoS Med. 2015;12(3):e1001779 10.1371/journal.pmed.1001779 25826379 PMC4380465

[22] Silventoinen K, Jelenkovic A, Sund R, Honda C, Aaltonen S, Yokoyama Y, et al The CODATwins Project: The Cohort Description of Collaborative Project of Development of Anthropometrical Measures in Twins to Study Macro-Environmental Variation in Genetic and Environmental Effects on Anthropometric Traits. Twin Res Hum Genet. 2015;18(4):348–60. 10.1017/thg.2015.29 26014041 PMC4696543

[23] Wang M, Xu S. Statistics of Mendelian segregation-A mixture model. J Anim Breed Genet. 2019;136(5):341–50. 10.1111/jbg.12394 31038229

[24] Lee JJ, Wedow R, Okbay A, Kong E, Maghzian O, Zacher M, et al Gene discovery and polygenic prediction from a genome-wide association study of educational attainment in 1.1 million individuals. Nat Genet. 2018;50(8):1112–21. 10.1038/s41588-018-0147-3 30038396 PMC6393768

[25] Okbay A, Beauchamp JP, Fontana MA, Lee JJ, Pers TH, Rietveld CA, et al Genome-wide association study identifies 74 loci associated with educational attainment. Nature. 2016;533(7604):539–42. 10.1038/nature17671 27225129 PMC4883595

[26] Yang JA, Benyamin B, McEvoy BP, Gordon S, Henders AK, Nyholt DR, et al Common SNPs explain a large proportion of the heritability for human height. Nature Genetics. 2010;42(7):565–U131. 10.1038/ng.608 20562875 PMC3232052

[27] Purcell S, Neale B, Todd-Brown K, Thomas L, Ferreira MA, Bender D, et al PLINK: a tool set for whole-genome association and population-based linkage analyses. Am J Hum Genet. 2007;81(3):559–75. 10.1086/519795 17701901 PMC1950838

[28] Lynch M, Walsh B. Genetics and analysis of quantitative traits: Sinauer Sunderland, MA; 1998.

[29] Sham PC, Cherny SS, Purcell S, Hewitt JK. Power of linkage versus association analysis of quantitative traits, by use of variance-components models, for sibship data. Am J Hum Genet. 2000;66(5):1616–30. 10.1086/302891 10762547 PMC1378020

[30] Spielman RS, Ewens WJ. A sibship test for linkage in the presence of association: the sib transmission/disequilibrium test. Am J Hum Genet. 1998;62(2):450–8. 10.1086/301714 9463321 PMC1376890

[31] Fulker DW, Cherny SS, Sham PC, Hewitt JK. Combined linkage and association sib-pair analysis for quantitative traits. Am J Hum Genet. 1999;64(1):259–67. 10.1086/302193 9915965 PMC1377724

[32] Dudbridge F, Holmans PA, Wilson SG. A flexible model for association analysis in sibships with missing genotype data. Ann Hum Genet. 2011;75(3):428–38. 10.1111/j.1469-1809.2010.00636.x 21241274

[33] Weinberg CR. Allowing for missing parents in genetic studies of case-parent triads. Am J Hum Genet. 1999;64(4):1186–93. 10.1086/302337 10090904 PMC1377843

[34] Rampersaud E, Morris RW, Weinberg CR, Speer MC, Martin ER. Power calculations for likelihood ratio tests for offspring genotype risks, maternal effects, and parent-of-origin (POO) effects in the presence of missing parental genotypes when unaffected siblings are available. Genet Epidemiol. 2007;31(1):18–30. 10.1002/gepi.20189 17096358 PMC2118060

[35] Gjerdevik M, Jugessur A, Haaland OA, Romanowska J, Lie RT, Cordell HJ, et al Haplin power analysis: a software module for power and sample size calculations in genetic association analyses of family triads and unrelated controls. BMC Bioinformatics. 2019;20(1):165 10.1186/s12859-019-2727-3 30940094 PMC6444579

[36] Howey R, Cordell HJ. PREMIM and EMIM: tools for estimation of maternal, imprinting and interaction effects using multinomial modelling. BMC Bioinformatics. 2012;13:149 10.1186/1471-2105-13-149 22738121 PMC3464602

[37] Wright J, Small N, Raynor P, Tuffnell D, Bhopal R, Cameron N, et al Cohort Profile: the Born in Bradford multi-ethnic family cohort study. Int J Epidemiol. 2013;42(4):978–91. 10.1093/ije/dys112 23064411

[38] Barker DJ. The fetal and infant origins of adult disease. BMJ. 1990;301(6761):1111 10.1136/bmj.301.6761.1111 2252919 PMC1664286

[39] Gusev A, Lowe JK, Stoffel M, Daly MJ, Altshuler D, Breslow JL, et al Whole population, genome-wide mapping of hidden relatedness. Genome Res. 2009;19(2):318–26. 10.1101/gr.081398.108 18971310 PMC2652213

[40] Hill WG, Weir BS. Variation in actual relationship as a consequence of Mendelian sampling and linkage. Genet Res (Camb). 2011;93(1):47–64. 10.1017/S0016672310000480 21226974 PMC3070763

[41] Hill WG, White IM. Identification of pedigree relationship from genome sharing. G3 (Bethesda). 2013;3(9):1553–71. 10.1534/g3.113.007500 23893739 PMC3755916

[42] Eaves L, Heath A, Martin N, Maes H, Neale M, Kendler K, et al Comparing the biological and cultural inheritance of personality and social attitudes in the Virginia 30,000 study of twins and their relatives. Twin Res. 1999;2(2):62–80. 10.1375/136905299320565933 10480741

[43] Clayton D. Testing for association on the X chromosome. Biostatistics. 2008;9(4):593–600. 10.1093/biostatistics/kxn007 18441336 PMC2536723

[44] Eaves LJ, Pourcain BS, Smith GD, York TP, Evans DM. Resolving the effects of maternal and offspring genotype on dyadic outcomes in genome wide complex trait analysis ("M-GCTA"). Behav Genet. 2014;44(5):445–55. 10.1007/s10519-014-9666-6 25060210 PMC4174369

