# Supplementary material for: Estimating indirect parental genetic effects on offspring phenotypes using virtual parental genotypes derived from sibling and half sibling pairs
Source: PLoS Genet. 2020 Oct 26;16(10):e1009154. doi: 10.1371/journal.pgen.1009154 (PMC7646364; doi:10.1371/journal.pgen.1009154)
Supplement: S1 Text — (DOCX) [file pgen.1009154.s001.docx]

**SUPPLEMENTARY MATERIALS**

Supplementary Methods p. 2

Derivation of the Variance of Imputed Parental Genotypes p. 2

for Sibling Pairs at Autosomal Loci

Derivation of non-centrality parameters p. 4

Sibling pairs autosomes p. 4

Maternal half sibling pairs autosomes p. 11

Siblings X chromosome loci p. 19

Simulation R Code p. 28

**SUPPLEMENTARY METHODS**

**Derivation of the Variance of Imputed Parental Genotypes for Sibling Pairs at Autosomal Loci**

Consider a pair of siblings who have been genotyped at a single biallelic locus with possible genotypes *AA*, *Aa*, and *aa*.

Let *p* be the frequency of allele *A*, and *q* = 1 – *p* be the frequency of allele *a*.

We assume that dosage is coded according to the number of copies of the *a* allele.

The marginal probability of observing a sibling pair with ordered genotypes *i* and *j* can be calculated by summing over the prior probability that the pair shares zero, one or two alleles identical by descent (IBD):

$$P\left( G_{1}=j,G_{2}=k \right)=\sum_{x\in\left\{ 0,1,2 \right\}} P\left( G_{1}=j,G_{2}=k|IBD=x \right)P(IBD=x)$$

where the prior IBD probabilities for sharing zero, one and two alleles are ¼, ½, and ¼ respectively. Explicit formulae for computing the marginal sibling pair probabilities are given in Supplementary Table 18.

The conditional probability of observing the parent’s genotype given the genotype of the sibling pair is given in Supplementary Table 1.

The joint probability of observing the parental genotype and sibling genotype is then equal to the product of the conditional probability of observing the parent’s genotype given the genotype of the siblings, multiplied by the marginal probability of the sibling genotypes.

$$P\left( G_{P}=i,G_{1}=j,G_{2}=k \right)=P\left( G_{P}=i|G_{1}=j,G_{2}=k \right)P(G_{1}=j,G_{2}=k)$$

The expected dosage of the imputed parental genotype given sibling one has genotype *j* and sibling two has genotype *k* is estimated using the following formula:

$${E(X}_{p}^{'}\left| G_{1}=j,G_{2}=k \right)= P\left( X_{P}=1|G_{1}=j,G_{2}=k \right)+2\times P\left( X_{P}=2|G_{1}=j,G_{2}=k \right)$$

The variance of the parental dosage imputed from full sibling pairs $Var(X_{p}^{'})$, can then be derived using the usual formula for the variance:

$$\mathrm{Var}\left( X_{p}^{'} \right)=E\left( {X_{p}^{'}}^{2} \right)-{(E\left( X_{p}^{'} \right))}^{2}$$

where the expected imputed parental dosage is calculated as:

$${E(X}_{p}^{'})=\sum_{i,j,k \in\{AA,Aa,aa\}} P\left( G_{P}=i,G_{1}=j,G_{2}=k \right){E(X}_{p}^{'}|G_{1}=j,G_{2}=k)$$

and the expected imputed parental dosage squared as:

$$E\left( {X_{p}^{'}}^{2} \right)=\sum_{i,j,k \in\{AA,Aa,aa\}} P\left( G_{P}=i,G_{1}=j,G_{2}=k \right)E({X_{p}^{'}}^{2}|G_{1}=j,G_{2}=k)$$

where $P\left( G_{P}=i,G_{1}=j,G_{2}=k \right)$ is the joint probability of observing parental genotype *i*, genotype *j* for sibling one, and genotype *k* for sibling two, ${E(X}_{p}^{'}|G_{1}=j,G_{2}=k$) is the expected dosage of the imputed parental genotype given sibling one has genotype *j* and sibling two has genotype *k*, and ${{E(X}_{p}^{'}}^{2}|G_{1}=j,G_{2}=k$) is the expected dosage squared of the imputed parental genotype given sibling one has genotype *j* and sibling two has genotype *k*.

Letting the heterozygosity *H* = 2*pq*, and dividing the result by *H* so that the variance of the true parental genotype is scaled to unit variance, the variance of the imputed parental genotype, $\mathrm{Var}(X_{p}^{'})$, imputed from full sibling pairs is:

$$\mathrm{Var}\left( X_{p}^{'} \right)=\frac{2H^{2}+5H+12}{4(H+2)(H+4)}$$

Similar formulae were used to calculate the variance of the imputed parental dosages in the case of X chromosomal loci and for half sibling pairs.

**Derivation of non-centrality parameters**

We derive the expected minus two log-likelihoods for siblings (autosomes), maternal half siblings (autosomes) and for male, female and opposite sex siblings (X chromosome).

**Siblings (Autosomes)**

The model for sibling pairs at autosomal loci is

$$Y_{1i}=bX_{1i}+dX_{m,i}+fX_{f,i}+\tau_{i}+\varepsilon_{1i}$$

$$Y_{2i}=bX_{2i}+dX_{m,i}+fX_{f,i}+\tau_{i}+\varepsilon_{2i}$$

$$X_{1i}=0.5(X_{m,i}+X_{p,i})+\eta_{1i}$$

$$X_{2i}=0.5(X_{m,i}+X_{p,i})+\eta_{2i}$$

where *Y_1_* and *Y_2_* are the phenotypes of siblings one and two, *X_1_*, *X_2_*, *X_m_* and *X_f_*  $\in$ {0, 1, 2} are the genotype dosages of siblings one and two and their mother and father respectively, *b*, *d* and *f* are the effect of the offspring, maternal and paternal genotypes on the offspring phenotype, *τ* is a random effect shared by the siblings, *ε*_1_ and *ε*_2_ are uncorrelated error terms for the two phenotypes, and *η*_1_ and *η*_2_ are random effects due to the segregation of alleles. Without loss of generality, effects were scaled so that the variance of the genotype dosages and phenotype terms was one. The variances of the random effects are:

$$\mathrm{Var}\left( \varepsilon_{1} \right)=\mathrm{Var}\left( \varepsilon_{2} \right)=\sigma^{2}$$

$$\mathrm{Var}\left( \tau\right)=\varphi^{2}$$

$$\mathrm{Var}\left( \eta\right)=0.5$$

We assume random mating so that Cov(*X_m_*, *X_f_*) = 0. The covariances between genotypes are:

Cov(*X*_1_, *X*_2_) = Cov(*X*_1_, *X*_m_) = Cov(*X*_2_, *X*_m_) = Cov(*X*_1_, *X*_f_) = Cov(*X*_2_, *X*_f_) = 0.5

the covariance between phenotypes and genotypes are:

Cov(*Y*_1,_ *X*_1_) = Cov(*Y*_2_, *X*_2_) = *b* + 0.5*(d + f)*

Cov(*Y*_1_, *X*_2_) = Cov(*Y*_2_, *X*_1_) = 0.5(*b* + *d + f)*

Cov(*Y*_1_, *X*_m_) = Cov(*Y*_2_, *X*_m_) = 0.5*b* + *d*

Cov(*Y*_1_, *X*_f_) = Cov(*Y*_2_, *X*_f_) = 0.5*b* + *f*

and the covariance between the two phenotypes is:

Cov*(Y_1_, Y_2_) = 0.5b^2^ + d^2^ + f^2^ + bd + bf +* $\varphi^{2}$

The phenotypic variance in the offspring phenotype (*Y*) can be decomposed as follows:

$$\mathrm{Var}\left( Y \right)=\left( b^{2}+d^{2}+f^{2}+bd+bf \right)+\varphi^{2}+\sigma^{2}=1$$

The variance of the true maternal and paternal genotypes prior to standardization is equivalent to the expected heterozygosity, given the allele frequencies *p* and *q*:

$$\mathrm{Var}\left( X_{m} \right)=\mathrm{Var}\left( X_{f} \right)=2pq=H$$

Full sibling relationships do not provide any information to distinguish between alleles of maternal versus paternal origin at autosomal loci, therefore the imputed maternal genotype $X_{m}^{'}$ will be equivalent to the imputed paternal genotype $X_{f}^{'}$. Thus, models using the imputed parental genotype $X_{p}^{'}$, estimate the parameter *c,* the combined effects of *d* and *f* such that *c = d + f.*

The variance of the imputed parental genotype, $\mathrm{Var}(X_{p}^{'})$, imputed from full sibling pairs is (see earlier in the Supplementary Materials for the derivation):

$$\mathrm{Var}\left( X_{p}^{'} \right)=\frac{2H^{2}+5H+12}{4(H+2)(H+4)}$$

The covariance between actual and imputed genotype is equal to the variance of the imputed genotype:

$$\mathrm{Cov}\left( X_{m},X_{p}^{'} \right)=\mathrm{Cov}\left( X_{f},X_{p}^{'} \right)=\mathrm{Var}(X_{p}^{'})$$

and the covariances between the imputed parental genotype and sib genotypes and phenotypes are:

$$\mathrm{Cov}\left( X_{p}^{'},X_{1} \right)=\mathrm{Cov}\left( X_{p}^{'},X_{2} \right)=0.5$$

$$\mathrm{Cov}\left( X_{p}^{'},Y_{1} \right)=\mathrm{Cov}\left( X_{p}^{'},Y_{2} \right)=b/2+c\times\mathrm{Var}\left( X_{p}^{'} \right)$$

**Power calculation under linear mixed model analysis for autosomal loci for sibling pairs**

When actual maternal and paternal genotypes are available, the linear mixed model is

$$Y_{1i}=bX_{1i}+dX_{m,i}+fX_{p,i}+\tau_{i}+\varepsilon_{1i}$$

$$Y_{2i}=bX_{2i}+dX_{m,i}+fX_{p,i}+\tau_{i}+\varepsilon_{2i}$$

The fixed effects *b*, *d,* and *f* may be estimated by generalised least squares (GLS), where the covariance matrix of random effects is:

$$\boldsymbol{\Omega}=\left( \begin{matrix} \sigma^{2}{+\varphi}^{2} & \varphi^{2} \\ \varphi^{2} & \sigma^{2}{+\varphi}^{2} \end{matrix} \right)$$

The inverse of the covariance matrix of random effects is:

$$\boldsymbol{\Omega}^{-1}=\frac{1}{\sigma^{2}(\sigma^{2}{+2\varphi}^{2})}\left( \begin{matrix} \sigma^{2}{+\varphi}^{2} & {-\varphi}^{2} \\ {-\varphi}^{2} & \sigma^{2}{+\varphi}^{2} \end{matrix} \right)$$

The asymptotic GLS estimates of a vector of parameters $\hat{\boldsymbol{\beta}}$ are given by

$$\hat{\boldsymbol{\beta}}={E\left( \mathbf{X}^{\mathbf{T}}\boldsymbol{\Omega}^{\mathbf{-1}}\mathbf{X} \right)}^{\mathbf{-1}}E\left( \mathbf{X}^{\mathbf{T}}\boldsymbol{\Omega}^{\mathbf{-1}}\mathbf{Y} \right)$$

We consider the following models:

Null model of no association

The residual covariance matrix is simply the covariance matrix of Y:

$$\boldsymbol{\Sigma}=\boldsymbol{\Sigma}_{\boldsymbol{Y}}$$

$$=\left( \begin{matrix} 1 & 0.5b^{2}+d^{2}+f^{2}+bd+bf+\varphi^{2} \\ 0.5b^{2}+d^{2}+f^{2}+bd+bf+\varphi^{2} & 1 \end{matrix} \right)$$

The expected minus two log-likelihood (-2lnL) of the model per sibling pair is therefore:

$$E(-2lnL)=ln({1-\left( 0.5b^{2}+d^{2}+f^{2}+bd+bf+\varphi^{2} \right)}^{2})+2$$

One parental genotype (maternal genotype) in the model:

The **X** matrix contains one column with elements $X_{m}$. The asymptotic GLS estimate of the regression coefficient of $X_{m}$ is:

$$\hat{d}=E\left( \mathbf{X}^{\mathbf{T}}\boldsymbol{\Omega}^{\mathbf{-1}}\mathbf{X} \right)^{\mathbf{-1}}E\left( \mathbf{X}^{\mathbf{T}}\boldsymbol{\Omega}^{\mathbf{-1}}\mathbf{Y} \right)$$

$$=E\left( 2\sigma^{2}X_{m}^{2} \right)^{-1}E\left( \sigma^{2}X_{m}\left( Y_{1}+Y_{2} \right) \right)$$

$$=\mathrm{Cov}\left( X_{m},Y \right)$$

$$=d+b/2$$

The residual covariance matrix is:

$$\boldsymbol{\Sigma}=E\left( \mathbf{Y}-\mathbf{X}\hat{d} \right)\left( \mathbf{Y}-\mathbf{X}\hat{d} \right)^{\boldsymbol{T}}$$

$$=E\left( \mathbf{YY}^{\boldsymbol{T}}-\mathbf{Y}\hat{d}^{T}\mathbf{X}^{\boldsymbol{T}}-\mathbf{X}\hat{d}\mathbf{Y}^{\boldsymbol{T}}+{\mathbf{X}\hat{d}\hat{d}}^{T}\mathbf{X}^{\boldsymbol{T}} \right)$$

$$=\boldsymbol{\Sigma}_{\mathbf{Y}}-\hat{d}\left( \boldsymbol{\Sigma}_{\mathbf{YX}}+\boldsymbol{\Sigma}_{\mathbf{XY}} \right)+\hat{d}^{2}\boldsymbol{\Sigma}_{\mathbf{X}}$$

where

$$\boldsymbol{\Sigma}_{\boldsymbol{YX}}=\left( \begin{matrix} b/2 + d & b/2 + d \\ b/2 + d & b/2 + d \end{matrix} \right)$$

$$\boldsymbol{\Sigma}_{\boldsymbol{XY}}=\left( \begin{matrix} b/2 + d & b/2 + d \\ b/2 + d & b/2 + d \end{matrix} \right)$$

$$\boldsymbol{\Sigma}_{\boldsymbol{X}}=\left( \begin{matrix} 1 & 1 \\ 1 & 1 \end{matrix} \right)$$

Therefore:

$$\boldsymbol{\Sigma}=\left( \begin{matrix} 1-\left( d+0.5b \right)^{2} & 0.25b^{2}+\varphi^{2} \\ 0.25b^{2}+\varphi^{2} & 1-\left( d+0.5b \right)^{2} \end{matrix} \right)$$

The expected -2lnL of the model per sibpair is therefore:

$$E(-2lnL)=\ln(\left( 1-\left( d+0.5b \right)^{2} \right)^{2}-{{(0.25b^{2}+\varphi}^{4})}^{2})+2$$

Both parental genotypes only in model (terms for $X_{m}$ and $X_{f}$ only):

The **X** matrix contains two columns with elements $X_{m}$ and $X_{f}$. The asymptotic GLS estimates for the regression coefficients of $X_{m}$ and $X_{f}$ are:

$$\hat{\boldsymbol{g}}=\left( \hat{d},\hat{f} \right)^{T}=E\left( \boldsymbol{X}^{\boldsymbol{T}}\boldsymbol{\Omega}^{\boldsymbol{-1}}\boldsymbol{X} \right)^{\boldsymbol{-1}}E\left( \boldsymbol{X}^{\boldsymbol{T}}\boldsymbol{\Omega}^{\boldsymbol{-1}}\boldsymbol{Y} \right)$$

$$=E\left( \begin{matrix} \frac{2X_{m}^{2}}{\sigma^{2}{+2\varphi}^{2}} & \frac{2X_{m}X_{f}}{\sigma^{2}{+2\varphi}^{2}} \\ \frac{2X_{m}X_{f}}{\sigma^{2}{+2\varphi}^{2}} & \frac{2X_{f}^{2}}{\sigma^{2}{+2\varphi}^{2}} \end{matrix} \right)^{-1}E\left( \begin{matrix} \frac{X_{m}\left( Y_{1}+Y_{2} \right)}{\sigma^{2}{+2\varphi}^{2}} \\ \frac{X_{f}\left( Y_{1}+Y_{2} \right)}{\sigma^{2}{+2\varphi}^{2}} \end{matrix} \right)$$

$$=\left( \begin{matrix} \frac{\mathrm{Cov}\left( X_{m},Y_{1} \right)+\mathrm{Cov}\left( X_{m},Y_{2} \right)}{2} & \frac{\mathrm{Cov}\left( X_{f},Y_{1} \right)+\mathrm{Cov}\left( X_{f},Y_{2} \right)}{2} \end{matrix} \right)^{T}$$

$$=\left( \begin{matrix} 0.5b+d & 0.5b+f \end{matrix} \right)^{T}$$

The residual covariance matrix is:

$$\boldsymbol{\Sigma}=E\left( \mathbf{Y}-\mathbf{X}\hat{\boldsymbol{g}} \right)\left( \mathbf{Y}-\mathbf{X}\hat{\boldsymbol{g}} \right)^{\boldsymbol{T}}$$

$$=\boldsymbol{\Sigma}_{\boldsymbol{Y}}-E(\boldsymbol{X}\hat{\boldsymbol{g}}\boldsymbol{Y}^{\boldsymbol{T}}+\mathbf{Y}{\hat{\boldsymbol{g}}}^{\boldsymbol{T}}\mathbf{X}^{\boldsymbol{T}}-\mathbf{X}\hat{\boldsymbol{g}}{\hat{\boldsymbol{g}}}^{\boldsymbol{T}}\mathbf{X}^{\boldsymbol{T}})$$

where

$$E\left( \mathbf{X}\hat{\boldsymbol{g}}\mathbf{Y}^{\boldsymbol{T}} \right)=E\left( \mathbf{Y}{\hat{\boldsymbol{g}}}^{\boldsymbol{T}}\mathbf{X}^{\mathbf{T}} \right)=\left( \begin{matrix} 0.5b^{2}+d^{2}+f^{2}+bd+bf & 0.5b^{2}+d^{2}+f^{2}+bd+bf \\ 0.5b^{2}+d^{2}+f^{2}+bd+bf & 0.5b^{2}+d^{2}+f^{2}+bd+bf \end{matrix} \right)$$

$$E\left( \boldsymbol{X}\hat{\boldsymbol{g}}{\hat{\boldsymbol{g}}}^{\boldsymbol{T}}\mathbf{X}^{\boldsymbol{T}} \right)=\left( \begin{matrix} 0.5b^{2}+d^{2}+f^{2}+bd+bf & 0.5b^{2}+d^{2}+f^{2}+bd+bf \\ 0.5b^{2}+d^{2}+f^{2}+bd+bf & 0.5b^{2}+d^{2}+f^{2}+bd+bf \end{matrix} \right)$$

Therefore:

$$\boldsymbol{\Sigma}=\left( \begin{matrix} 1-(0.5b^{2}+d^{2}+f^{2}+bd+bf) & \varphi^{2} \\ \varphi^{2} & 1-(0.5b^{2}+d^{2}+f^{2}+bd+bf) \end{matrix} \right)$$

The expected -2lnL of the model per sibling pair is therefore:

$$E(-2lnL)={ln(\left( 1-(0.5b^{2}+d^{2}+f^{2}+bd+bf \right))}^{2}-\varphi^{4})+2$$

Offspring genotypes in model only (terms for $X_{1}$ and $X_{2}$ only):

The **X** matrix contains one column with elements $X_{1}$ and $X_{2}$. The asymptotic GLS estimate of the regression coefficient of $X_{1}$ and $X_{2}$ is:

$$\hat{b}=E\left( \mathbf{X}^{\mathbf{T}}\boldsymbol{\Omega}^{\mathbf{-1}}\mathbf{X} \right)^{\mathbf{-1}}E\left( \mathbf{X}^{\mathbf{T}}\boldsymbol{\Omega}^{\mathbf{-1}}\mathbf{Y} \right)$$

$$=E\left( \left( \sigma^{2}+\varphi^{2} \right)\left( X_{1}^{2}+X_{2}^{2} \right)-2X_{1}X_{2}\varphi^{2} \right)^{-1}E\left( \left( \sigma^{2}+\varphi^{2} \right)\left( X_{1}Y_{1}+X_{2}Y_{2} \right)-\varphi^{2}\left( X_{1}Y_{2}+X_{2}Y_{1} \right) \right)$$

$$=b+\left( \frac{\sigma^{2}}{2\sigma^{2}+\varphi^{2}} \right)(d+f)$$

The residual covariance matrix is:

$$\boldsymbol{\Sigma}=E\left( \mathbf{Y}-\mathbf{X}\hat{b} \right)\left( \mathbf{Y}-\mathbf{X}\hat{b} \right)^{T}$$

$$=\boldsymbol{\Sigma}_{\mathbf{Y}}-\hat{b}\left( \boldsymbol{\Sigma}_{\mathbf{YX}}+\boldsymbol{\Sigma}_{\mathbf{XY}} \right)+\hat{b}^{2}\boldsymbol{\Sigma}_{\mathbf{X}}$$

where:

$$\boldsymbol{\Sigma}_{\mathbf{YX}}=\left( \begin{matrix} b+0.5(d+f) & 0.5(b+d+f) \\ 0.5(b+d+f) & b + 0.5(d+f) \end{matrix} \right)$$

$$\boldsymbol{\Sigma}_{\mathbf{XY}}=\left( \begin{matrix} b+0.5(d+f) & 0.5(b+d+f) \\ 0.5(b+d+f) & b + 0.5(d+f) \end{matrix} \right)$$

$$\boldsymbol{\Sigma}_{\mathbf{X}}=\left( \begin{matrix} 1 & 0.5 \\ 0.5 & 1 \end{matrix} \right)$$

Therefore:

$$\boldsymbol{\Sigma}=\left( \begin{matrix} 1-\left( 2b+d+f \right)\hat{b}+\hat{b}^{2} & 0.5b^{2}+d^{2}+f^{2}+bd+bf+\varphi^{2}-\left( b+d+f \right)\hat{b}+0.5\hat{b}^{2} \\ 0.5b^{2}+d^{2}+f^{2}+bd+bf+\varphi^{2}-\left( b+d+f \right)\hat{b}+0.5\hat{b}^{2} & 1-\left( 2b+d+f \right)\hat{b}+\hat{b}^{2} \end{matrix} \right)$$

The expected -2lnL of the model per sibling pair is:

$$E(-2lnL)=ln|\boldsymbol{\Sigma}|+2$$

Full Omnibus Model (terms for $X_{m}$, $X_{f}$, $X_{1}$ and $X_{2}$):

The **X** matrix contains three columns; column 1 with elements $X_{1}$ and $X_{2}$, column 2 with elements $X_{m}$ and $X_{m}$, and column 3 with elements $X_{f}$ and $X_{f}$. The asymptotic GLS estimate of the regression coefficients of columns 1, 2 and 3 are:

$$\hat{\boldsymbol{g}}={(\hat{b},\hat{d,}\hat{f})}^{T}=E\left( \mathbf{X}^{\mathbf{T}}\boldsymbol{\Omega}^{\mathbf{-1}}\mathbf{X} \right)^{\mathbf{-1}}E\left( \mathbf{X}^{\mathbf{T}}\boldsymbol{\Omega}^{\mathbf{-1}}\mathbf{Y} \right)$$

$$={(b,d,f)}^{T}$$

The residual covariance matrix is:

$$\boldsymbol{\Sigma}=E\left( \mathbf{Y}\boldsymbol{-}\mathbf{X}\hat{\boldsymbol{g}} \right)\left( \mathbf{Y}\boldsymbol{-}\mathbf{X}\hat{\boldsymbol{g}} \right)^{\boldsymbol{T}}$$

$$=\boldsymbol{\Omega}$$

$$=\left( \begin{matrix} \sigma^{2}{+\varphi}^{2} & \varphi^{2} \\ \varphi^{2} & \sigma^{2}{+\varphi}^{2} \end{matrix} \right)$$

The expected -2lnL of the model per sibling pair is:

$$E(-2lnL)=ln|\boldsymbol{\Sigma}|+2$$

Imputed parental genotypes only in model (terms for $X_{p}^{'}$ only)

When only imputed parental genotypes are available, the linear mixed model becomes:

$$Y_{1i}=bX_{1i}+cX_{p,i}^{'}+\tau_{i}+\varepsilon_{1i}$$

$$Y_{2i}=bX_{2i}+cX_{p,i}^{'}+\tau_{i}+\varepsilon_{2i}$$

The **X** matrix contains one column with elements $X_{p}^{'}$. The asymptotic GLS estimate of the regression coefficient of $X_{p}^{'}$ is:

$$\hat{c}=E\left( \mathbf{X}^{\mathbf{T}}\boldsymbol{\Omega}^{\mathbf{-1}}\mathbf{X} \right)^{\mathbf{-1}}E\left( \mathbf{X}^{\mathbf{T}}\boldsymbol{\Omega}^{\mathbf{-1}}\mathbf{Y} \right)$$

$$=E\left( \frac{2X_{p}^{'2}}{\sigma^{2}{+2\varphi}^{2}} \right)^{-1}E\left( \frac{X_{p}^{'} \left( Y_{1}+Y_{2} \right)}{\sigma^{2}{+2\varphi}^{2}} \right)$$

$$=\frac{\mathrm{Cov}\left( X_{p}^{'},Y \right)}{\mathrm{Var}\left( X_{p}^{'} \right)}=\frac{0.5b+c\times\mathrm{Var}\left( X_{p}^{'} \right)}{\mathrm{Var}(X_{p}^{'})}=c+\frac{0.5b}{\mathrm{Var}(X_{p}^{'})}$$

The residual covariance matrix is:

$$\boldsymbol{\Sigma}=E\left( \mathbf{Y}-\mathbf{X}\hat{c} \right)\left( \mathbf{Y}-\mathbf{X}\hat{c} \right)^{\boldsymbol{T}}$$

$$=E\left( \mathbf{YY}^{\boldsymbol{T}}-\mathbf{Y}\hat{c}^{T}\mathbf{X}^{\boldsymbol{T}}-\mathbf{X}\hat{c}\mathbf{Y}^{\boldsymbol{T}}+{\mathbf{X}\hat{c}\hat{c}}^{T}\mathbf{X}^{\boldsymbol{T}} \right)$$

$$=\boldsymbol{\Sigma}_{\mathbf{Y}}-\hat{c}\left( \boldsymbol{\Sigma}_{\mathbf{YX}}+\boldsymbol{\Sigma}_{\mathbf{XY}} \right)+\hat{c}^{2}\boldsymbol{\Sigma}_{\mathbf{X}}$$

where:

$$\boldsymbol{\Sigma}_{\mathbf{YX}}=\left( \begin{matrix} 0.5b + c\times\mathrm{Var}\left( X_{p}^{'} \right) & 0.5b + c\times\mathrm{Var}\left( X_{p}^{'} \right) \\ 0.5b + c\times\mathrm{Var}\left( X_{p}^{'} \right) & 0.5b + c\times\mathrm{Var}\left( X_{p}^{'} \right) \end{matrix} \right)$$

$$\boldsymbol{\Sigma}_{\mathbf{XY}}=\left( \begin{matrix} 0.5b + c\times\mathrm{Var}\left( X_{p}^{'} \right) & 0.5b + c\times\mathrm{Var}\left( X_{p}^{'} \right) \\ 0.5b + c\times\mathrm{Var}\left( X_{p}^{'} \right) & 0.5b + c\times\mathrm{Var}\left( X_{p}^{'} \right) \end{matrix} \right)$$

$$\boldsymbol{\Sigma}_{\mathbf{X}}=\left( \begin{matrix} \mathrm{Var}\left( X_{p}^{'} \right) & \mathrm{Var}\left( X_{p}^{'} \right) \\ \mathrm{Var}\left( X_{p}^{'} \right) & \mathrm{Var}\left( X_{p}^{'} \right) \end{matrix} \right)$$

The expected -2lnL of the model per sibling pair is:

$$E(-2lnL)=ln|\boldsymbol{\Sigma}|+2$$

Full omnibus model with imputed parental genotypes (terms for $X_{p}^{'}$, $X_{1}$ and $X_{2}$)

The **X** matrix contains two columns; column 1 with elements $X_{1}$ and $X_{2}$, and column 2 with elements $X_{p}^{'}$ and $X_{p}^{'}$. The asymptotic GLS estimate of the regression coefficients of columns 1 and 2 are:

$${\hat{\boldsymbol{g}}=(\hat{b},\hat{c})}^{T}=E\left( \mathbf{X}^{\mathbf{T}}\boldsymbol{\Omega}^{\mathbf{-1}}\mathbf{X} \right)^{\mathbf{-1}}E\left( \mathbf{X}^{\mathbf{T}}\boldsymbol{\Omega}^{\mathbf{-1}}\mathbf{Y} \right)$$

$$={(b,c)}^{T}$$

The residual covariance matrix is:

$$\boldsymbol{\Sigma}=E\left( \mathbf{Y}\boldsymbol{-}\mathbf{X}\hat{\boldsymbol{g}} \right)\left( \mathbf{Y}\boldsymbol{-}\mathbf{X}\hat{\boldsymbol{g}} \right)^{\boldsymbol{T}}$$

$$=\boldsymbol{\Sigma}_{\boldsymbol{Y}}-E(\mathbf{X}\hat{\boldsymbol{g}}\mathbf{Y}^{\boldsymbol{T}}+\mathbf{Y}{\hat{\boldsymbol{g}}}^{\boldsymbol{T}}\mathbf{X}^{\boldsymbol{T}}-\mathbf{X}\hat{\boldsymbol{g}}{\hat{\boldsymbol{g}}}^{\boldsymbol{T}}\mathbf{X}^{\boldsymbol{T}})$$

where:

$$E(\mathbf{X}\hat{\boldsymbol{g}}\mathbf{Y}^{\boldsymbol{T}})=E(\mathbf{Y}{\hat{\boldsymbol{g}}}^{\boldsymbol{T}}\mathbf{X}^{\boldsymbol{T}})=\left( \begin{matrix} \hat{b}\times\mathrm{Cov}\left( X_{1},Y_{1} \right)+\hat{c\times}\mathrm{Cov}(X_{p}^{'},Y_{1}) & \hat{b}\times\mathrm{Cov}\left( X_{2},Y_{1} \right)+\hat{c}\times\mathrm{Cov}(X_{p}^{'},Y_{1}) \\ \hat{b}\times\mathrm{Cov}\left( X_{1},Y_{2} \right)+\hat{c}\times\mathrm{Cov}(X_{p}^{'},Y_{2}) & \hat{b}\times\mathrm{Cov}\left( X_{2},Y_{2} \right)+\hat{c}\times\mathrm{Cov}(X_{p}^{'},Y_{2}) \end{matrix} \right)$$

and:

$$E\left( \mathbf{X}\hat{\boldsymbol{g}}{\hat{\boldsymbol{g}}}^{\boldsymbol{T}}\mathbf{X}^{\boldsymbol{T}} \right)=\left( \begin{matrix} \hat{b}^{2}+\hat{b}\hat{c}+c^{2}\times\mathrm{Var}(X_{p}^{'}) & {0.5\hat{b}}^{2}+\hat{b}\hat{c}+\hat{c}^{2}\times\mathrm{Var}(X_{p}^{'}) \\ {0.5\hat{b}}^{2}+\hat{b}\hat{c}+\hat{c}^{2}\times\mathrm{Var}(X_{p}^{'}) & \hat{b}^{2}+\hat{b}\hat{c}+c^{2}\times\mathrm{Var}(X_{p}^{'}) \end{matrix} \right)$$

The expected -2lnL of the model per sibling pair is:

$$E(-2lnL)=ln|\boldsymbol{\Sigma}|+2$$

**Maternal Half Siblings (Autosomes)**

The model for maternal half sibling pair is

$$Y_{1i}=bX_{1i}+dX_{m,i}+fX_{f1,i}+\tau_{i}+\varepsilon_{1i}$$

$$Y_{2i}=bX_{2i}+dX_{m,i}+fX_{f2,i}+\tau_{i}+\varepsilon_{2i}$$

$$X_{1i}=0.5(X_{m,i}+X_{f1,i})+\eta_{1i}$$

$$X_{2i}=0.5(X_{m,i}+X_{f2,i})+\eta_{2i}$$

We assume random mating, so that Cov(X_m_,X_f_) = Cov(X_m_,X_f1_) = Cov(X_m_,X_f2_) = 0

So that the covariances between genotypes are

Cov(*X*_1_,*X*_2_) = 0.25

Cov(*X*_1_,*X*_m_) = Cov(*X*_2_,*X*_m_) = Cov(*X*_1_,*X*_f1_) = Cov(*X*_2_,*X*_f2_) = 0.5

Cov(*X*_1_,*X*_f2_) = Cov(*X*_2_,*X*_f1_) = 0

Cov(*X*_f1_,*X*_f2_) = 0

The covariances between phenotypes and genotypes are then

Cov(*Y*_1,_*X*_1_) = Cov(*Y*_2_,*X*_2_) = *b* + *0.5(d+f)*

Cov(*Y*_1_,*X*_2_) = Cov(*Y*_2_,*X*_1_) = 0.25*b +* 0.5*d*

Cov(*Y*_1_,*X*_m_) = Cov(*Y*_2_,*X*_m_) = 0.5*b* + *d*

Cov(*Y*_1_,*X*_f1_) = Cov(*Y*_2_,*X*_f2_) = 0.5*b + f*

Cov(*Y*_1_,*X*_f2_) = Cov(*Y*_2_,*X*_f1_) = 0

The covariance of the two phenotypes is

Cov*(Y_1_,Y_2_) =* 0.25*b^2^ + d^2^ + bd +* $\varphi^{2}$

The phenotypic variance can be decomposed as follows

$$\mathrm{Var}\left( Y \right)=\left( b^{2}+d^{2}+f^{2}+bd+bf \right)+\varphi^{2}+\sigma^{2}=1$$

The variance of the true parental genotypes prior to standardization is equivalent to the expected heterozygosity, given the allele frequencies *p* and *q*

$$\mathrm{Var}\left( X_{m} \right)=\mathrm{Var}\left( X_{f} \right)=2pq=H$$

The variance of the imputed maternal and paternal genotypes are functions of the expected heterozygosity such that

$$\mathrm{Var}\left( X_{m}^{'} \right)=\frac{5(H+1)}{(H+4)(2H+3)}$$

$$\mathrm{Var}\left( X_{f}^{'} \right)=\frac{{(H+1)}^{2}}{(2H+3)(2H+1)}$$

The covariance between respective actual and imputed genotypes is equal to the variance of the imputed genotype:

$$\mathrm{Cov}\left( X_{m},X_{m}^{'} \right)=\mathrm{Var}(X_{m}^{'})$$

$$\mathrm{Cov}\left( X_{f},X_{f}^{'} \right)=\mathrm{Var}(X_{f}^{'})$$

The covariance between the two imputed paternal genotypes is a function of H:

$$\mathrm{Cov}\left( X_{f1}^{'},X_{f2}^{'} \right)= \frac{-H}{8H+4}$$

as is the covariance between the imputed paternal and imputed maternal genotypes:

$$\mathrm{Cov}\left( X_{m}^{'},X_{f1}^{'} \right)=\mathrm{Cov}\left( X_{m}^{'},X_{f2}^{'} \right)= \frac{H+1}{4H+6}$$

The covariance between the imputed parental and sib genotypes and phenotypes are:

$$\mathrm{Cov}\left( X_{m}^{'},X_{1} \right)=\mathrm{Cov}\left( X_{m}^{'},X_{2} \right)=\mathrm{Cov}\left( X_{f1}^{'},X_{1} \right)=\mathrm{Cov}\left( X_{f2}^{'},X_{2} \right)=0.5$$

$$\mathrm{Cov}\left( X_{m}^{'},Y_{1} \right)=\mathrm{Cov}\left( X_{m}^{'},Y_{2} \right)=0.5b+d\times\mathrm{Var}\left( X_{m}^{'} \right)+f\times\mathrm{Cov}\left( X_{m}^{'},X_{f}^{'} \right)$$

$$\mathrm{Cov}\left( X_{f1}^{'},Y_{1} \right)=\mathrm{Cov}\left( X_{f2}^{'},Y_{2} \right)=0.5b+d\times\mathrm{Cov}\left( X_{m}^{'},X_{f}^{'} \right)+f\times\mathrm{Var}\left( X_{f}^{'} \right)$$

$$\mathrm{Cov}\left( X_{f1}^{'},Y_{2} \right)=\mathrm{Cov}\left( X_{f2}^{'},Y_{1} \right)=d\times\mathrm{Cov}\left( X_{m}^{'},X_{f}^{'} \right)+f\times\mathrm{Cov}\left( X_{f1}^{'},X_{f2}^{'} \right)$$

$$\mathrm{Cov}\left( X_{f1}^{'},X_{2} \right)=\mathrm{Cov}\left( X_{f2}^{'},X_{1} \right)=0$$

**Power calculation under linear mixed model analysis for autosomal loci for maternal half siblings**

When actual maternal and paternal genotypes are available, the linear mixed model is

$$Y_{1i}=bX_{1i}+dX_{m,i}+fX_{f1,i}+\tau_{i}+\varepsilon_{1i}$$

$$Y_{2i}=bX_{2i}+dX_{m,i}+fX_{f2,i}+\tau_{i}+\varepsilon_{2i}$$

The fixed effects *b* and *d* are estimated by generalised least squares (GLS), where the covariance matrix of random effects is:

$$\boldsymbol{\Omega}=\left( \begin{matrix} \sigma^{2}{+\varphi}^{2} & \varphi^{2} \\ \varphi^{2} & \sigma^{2}{+\varphi}^{2} \end{matrix} \right)$$

The inverse of the covariance matrix of random effects is

$$\boldsymbol{\Omega}^{\boldsymbol{-1}}=\frac{1}{\sigma^{2}(\sigma^{2}{+2\varphi}^{2})}\left( \begin{matrix} \sigma^{2}{+\varphi}^{2} & {-\varphi}^{2} \\ {-\varphi}^{2} & \sigma^{2}{+\varphi}^{2} \end{matrix} \right)$$

The asymptotic GLS estimates of a vector of parameters $\boldsymbol{\beta}$ are given by

$$\hat{\boldsymbol{\beta}}={E\left( \mathbf{X}^{\boldsymbol{T}}\boldsymbol{\Omega}^{\boldsymbol{-1}}\mathbf{X} \right)}^{\boldsymbol{-1}}E\left( \mathbf{X}^{\boldsymbol{T}}\boldsymbol{\Omega}^{\mathbf{-1}}\mathbf{Y} \right)$$

We consider the following models:

Null model of no association

The residual covariance matrix is simply the covariance matrix of Y:

$$\boldsymbol{\Sigma}=\boldsymbol{\Sigma}_{\boldsymbol{Y}}$$

$$=\left( \begin{matrix} 1 & 0.25b^{2} + d^{2} + bd + \varphi^{2} \\ 0.25b^{2} + d^{2} + bd + \varphi^{2} & 1 \end{matrix} \right)$$

The expected -2lnL of the model per sibpair is therefore

$$E(-2lnL)={ln(1-\left( 0.25b^{2} + d^{2} + bd + \varphi^{2} \right)}^{2})+2$$

Both parental genotypes in model (terms for $X_{m}$ and $X_{f}$ only):

The **X** matrix contains one column with elements $X_{m}$ and another with elements $X_{f1}$ and $X_{f2}$. The asymptotic GLS estimates of the regression coefficients of $X_{m}$ and $X_{f}$ are

$$\hat{\boldsymbol{g}}=\left( \hat{d},\hat{f} \right)^{T}=E\left( \mathbf{X}^{\boldsymbol{T}}\boldsymbol{\Omega}^{\boldsymbol{-1}}\mathbf{X} \right)^{-1}E\left( \mathbf{X}^{\boldsymbol{T}}\boldsymbol{\Omega}^{\boldsymbol{-1}}\mathbf{Y} \right)$$

$$=E\left( \begin{matrix} \frac{2X_{m}^{2}}{\sigma^{2}{+2\varphi}^{2}} & \frac{X_{m}X_{f1}+X_{m}X_{f2}}{\sigma^{2}{+2\varphi}^{2}} \\ \frac{X_{m}X_{f1}+X_{m}X_{f2}}{\sigma^{2}{+2\varphi}^{2}} & \frac{{(X}_{f1}^{2}+X_{f2}^{2})\left( \sigma^{2}{+\varphi}^{2} \right)-2\varphi^{2}X_{f1}X_{f2}}{\sigma^{2}(\sigma^{2}{+2\varphi}^{2})} \end{matrix} \right)^{-1}E\left( \begin{matrix} \frac{X_{m}\left( Y_{1}+Y_{2} \right)}{\sigma^{2}{+2\varphi}^{2}} \\ \frac{{(\sigma}^{2}{+\varphi}^{2})\left( Y_{1}X_{f1}+Y_{2}X_{f2} \right)-\varphi^{2}\left( Y_{1}X_{f2}+Y_{2}X_{f1} \right)}{\sigma^{2}(\sigma^{2}{+2\varphi}^{2})} \end{matrix} \right)$$

$$=\left( \begin{matrix} \frac{\mathrm{Cov}\left( X_{m},Y_{1} \right)+\mathrm{Cov}\left( X_{m},Y_{2} \right)}{2} & \frac{\mathrm{Cov}\left( X_{f1},Y_{1} \right)+\mathrm{Cov}\left( X_{f2},Y_{2} \right)}{2} \end{matrix} \right)^{T}$$

$$=\left( \begin{matrix} 0.5b+d & 0.5b+f \end{matrix} \right)^{T}$$

The residual covariance matrix is

$$\boldsymbol{\Sigma}=E\left( \mathbf{Y}-\mathbf{X}\hat{\boldsymbol{g}} \right)\left( \mathbf{Y}\boldsymbol{-}\mathbf{X}\hat{\boldsymbol{g}} \right)^{\boldsymbol{T}}$$

$$=\boldsymbol{\Sigma}_{\boldsymbol{Y}}-E(\mathbf{X}\hat{\boldsymbol{g}}\mathbf{Y}^{\boldsymbol{T}}+\mathbf{Y}{\hat{\boldsymbol{g}}}^{\boldsymbol{T}}\mathbf{X}^{\boldsymbol{T}}-\mathbf{X}\hat{\boldsymbol{g}}{\hat{\boldsymbol{g}}}^{\boldsymbol{T}}\mathbf{X}^{\boldsymbol{T}})$$

where

$$E\boldsymbol{(}\mathbf{X}\hat{\boldsymbol{g}}\mathbf{Y}^{\boldsymbol{T}}\boldsymbol{)}=E\boldsymbol{(}\mathbf{Y}{\hat{\boldsymbol{g}}}^{\boldsymbol{T}}\mathbf{X}^{\boldsymbol{T}}\boldsymbol{)}=\left( \begin{matrix} \hat{d}\times\mathrm{Cov}\left( X_{m},Y_{1} \right)+\hat{f}\times\mathrm{Cov}(X_{f1},Y_{1}) & \hat{d}\times\mathrm{Cov}\left( X_{m},Y_{2} \right) \\ \hat{d}\times\mathrm{Cov}\left( X_{m},Y_{1} \right) & \hat{d}\times\mathrm{Cov}\left( X_{m},Y_{2} \right)+\hat{f}\times\mathrm{Cov}(X_{f2},Y_{2}) \end{matrix} \right)$$

$$E\left( \mathbf{X}\hat{\boldsymbol{g}}{\hat{\boldsymbol{g}}}^{\boldsymbol{T}}\mathbf{X}^{\boldsymbol{T}} \right)=\left( \begin{matrix} \hat{d}^{2}+\hat{f}^{2} & \hat{d}^{2} \\ \hat{d}^{2} & \hat{d}^{2}+\hat{f}^{2} \end{matrix} \right)$$

Therefore

$$\boldsymbol{\Sigma}=\left( \begin{matrix} 1-{(0.5b+d)}^{2} & \varphi^{2} \\ \varphi^{2} & 1-{(0.5b+d)}^{2} \end{matrix} \right)$$

The expected -2lnL of the model per half sibling pair is:

$$E(-2lnL)=\ln|\boldsymbol{\Sigma}|+2$$

Offspring genotypes in model only (terms $X_{1}$ and $X_{2}$ only):

The **X** matrix contains one column with elements $X_{1}$ and $X_{2}$. The asymptotic GLS estimate of the regression coefficient of $X_{1}$ and $X_{2}$ is

$$\hat{b}=E\left( \mathbf{X}^{\boldsymbol{T}}\boldsymbol{\Omega}^{\boldsymbol{-1}}\mathbf{X} \right)^{\boldsymbol{-1}}E\left( \mathbf{X}^{\boldsymbol{T}}\boldsymbol{\Omega}^{\boldsymbol{-1}}\mathbf{Y} \right)$$

$$=E\left( \left( \sigma^{2}+\varphi^{2} \right)\left( X_{1}^{2}+X_{2}^{2} \right)-2X_{1}X_{2}\varphi^{2} \right)^{-1}E\left( \left( \sigma^{2}+\varphi^{2} \right)\left( X_{1}Y_{1}+X_{2}Y_{2} \right)-\varphi^{2}\left( X_{1}Y_{2}+X_{2}Y_{1} \right) \right)$$

$$=\frac{\left( \sigma^{2}+\varphi^{2} \right)\left( \mathrm{Cov}\left( X_{1},Y_{1} \right)+\mathrm{Cov}\left( X_{2},Y_{2} \right) \right)-\varphi^{2}\left( \mathrm{Cov}\left( X_{1},Y_{2} \right)+\mathrm{Cov}\left( X_{2},Y_{1} \right) \right)}{\left( \sigma^{2}+\varphi^{2} \right)\left( \mathrm{Var}\left( X_{1} \right)+\mathrm{Var}\left( X_{2} \right) \right)-2\varphi^{2}\times Cov(X_{1},X_{2})}$$

$$=b+\frac{\left( {d\sigma}^{2}+f\sigma^{2}+f\varphi^{2} \right)}{\left( {2\sigma}^{2}+2\varphi^{2}-0.5\varphi^{2} \right)}$$

The residual covariance matrix is

$$\boldsymbol{\Sigma}=E\left( \mathbf{Y}-\mathbf{X}\hat{b} \right)\left( \mathbf{Y}-\mathbf{X}\hat{b} \right)^{T}$$

$$=\boldsymbol{\Sigma}_{\mathbf{Y}}-\hat{b}\left( \boldsymbol{\Sigma}_{\mathbf{YX}}+\boldsymbol{\Sigma}_{\mathbf{XY}} \right)+\hat{b}^{2}\boldsymbol{\Sigma}_{\mathbf{X}}$$

Where

$$\boldsymbol{\Sigma}_{\mathbf{YX}}=\left( \begin{matrix} b + 0.5(d+f) & 0.25b + 0.5d \\ 0.25b + 0.5d & b + 0.5(d+f) \end{matrix} \right)$$

$$\boldsymbol{\Sigma}_{\mathbf{XY}}=\left( \begin{matrix} b + 0.5(d+f) & 0.25b + 0.5d \\ 0.25b + 0.5d & b + 0.5(d+f) \end{matrix} \right)$$

$$\boldsymbol{\Sigma}_{\mathbf{X}}=\left( \begin{matrix} 1 & 0.25 \\ 0.25 & 1 \end{matrix} \right)$$

Therefore

$$\boldsymbol{\Sigma}=\left( \begin{matrix} 1-\left( 2b+d+f \right)\hat{b}+\hat{b}^{2} & 0.25b^{2} + d^{2} + bd + \varphi^{2}-\left( 0.5b+d \right)\hat{b}+0.25\hat{b}^{2} \\ 0.25b^{2} + d^{2} + bd + \varphi^{2}-\left( 0.5b+d \right)\hat{b}+0.25\hat{b}^{2} & 1-\left( 2b+d+f \right)\hat{b}+\hat{b}^{2} \end{matrix} \right)$$

The expected -2lnL of the model per half sibling pair is:

$$E(-2lnL)=ln|\boldsymbol{\Sigma}|+2$$

Full Omnibus Model (terms for $X_{m}$, $X_{f}$, $X_{1}$ and $X_{2}$):

The **X** matrix contains three columns; column 1 with elements $X_{1}$ and $X_{2}$, column 2 with elements $X_{m}$ and $X_{m}$, and column 3 with $X_{f1}$ and $X_{f2}$. The asymptotic GLS estimate of the regression coefficients of columns 1 and 2 are

$$\hat{\boldsymbol{g}}={(\hat{b},\hat{d},\hat{f})}^{T}=E\left( \mathbf{X}^{\boldsymbol{T}}\boldsymbol{\Omega}^{\boldsymbol{-1}}\mathbf{X} \right)^{\boldsymbol{-1}}E\left( \mathbf{X}^{\boldsymbol{T}}\boldsymbol{\Omega}^{\boldsymbol{-1}}\mathbf{Y} \right)$$

$$={(b,d, f)}^{T}$$

The residual covariance matrix is

$$\boldsymbol{\Sigma}=E\left( \mathbf{Y}\boldsymbol{-}\mathbf{X}\hat{\boldsymbol{g}} \right)\left( \mathbf{Y}\boldsymbol{-}\mathbf{X}\hat{\boldsymbol{g}} \right)^{\boldsymbol{T}}$$

$$=\boldsymbol{\Omega}$$

$$=\left( \begin{matrix} \sigma^{2}{+\varphi}^{2} & \varphi^{2} \\ \varphi^{2} & \sigma^{2}{+\varphi}^{2} \end{matrix} \right)$$

The expected -2lnL of the model per half sibling pair is:

$$E(-2lnL)=ln|\boldsymbol{\Sigma}|+2$$

Imputed parental genotypes only in model (terms for $X_{m}^{'}$ and $X_{f}^{'}$):

When only imputed maternal genotypes are available, the linear mixed model becomes

$$Y_{1i}=bX_{1i}+dX_{m,i}^{'}+fX_{f1,i}^{'}+\tau_{i}+\varepsilon_{1i}$$

$$Y_{2i}=bX_{2i}+dX_{m,i}^{'}+fX_{f2,i}^{'}+\tau_{i}+\varepsilon_{2i}$$

The **X** matrix contains one column with elements $X_{m}^{'}$ and another with elements $X_{f1}^{'}$ and $X_{f2}^{'}$. The asymptotic GLS estimates of the regression coefficients of $X_{m}^{'}$ and $X_{f}^{'}$ are

$$\hat{\boldsymbol{g}}=\left( \hat{d},\hat{f} \right)^{T}=E\left( \mathbf{X}^{\boldsymbol{T}}\boldsymbol{\Omega}^{\boldsymbol{-1}}\mathbf{X} \right)^{\boldsymbol{-1}}E\left( \mathbf{X}^{\boldsymbol{T}}\boldsymbol{\Omega}^{\boldsymbol{-1}}\mathbf{Y} \right)$$

$$=E\left( \begin{matrix} \frac{2{X_{m}^{'}}^{2}}{\sigma^{2}{+2\varphi}^{2}} & \frac{X_{m}^{'}X_{f1}^{'}+X_{m}^{'}X_{f2}^{'}}{\sigma^{2}{+2\varphi}^{2}} \\ \frac{X_{m}^{'}X_{f1}^{'}+X_{m}^{'}X_{f2}^{'}}{\sigma^{2}{+2\varphi}^{2}} & \frac{({X_{f1}^{'}}^{2}+{X_{f2}^{'}}^{2})\left( \sigma^{2}{+\varphi}^{2} \right)-2\varphi^{2}X_{f1}^{'}X_{f2}^{'}}{\sigma^{2}(\sigma^{2}{+2\varphi}^{2})} \end{matrix} \right)^{-1}E\left( \begin{matrix} \frac{X_{m}^{'}\left( Y_{1}+Y_{2} \right)}{\sigma^{2}{+2\varphi}^{2}} \\ \frac{{(\sigma}^{2}{+\varphi}^{2})\left( Y_{1}X_{f1}^{'}+Y_{2}X_{f2}^{'} \right)-\varphi^{2}\left( Y_{1}X_{f2}^{'}+Y_{2}X_{f1}^{'} \right)}{\sigma^{2}(\sigma^{2}{+2\varphi}^{2})} \end{matrix} \right)$$

The residual covariance matrix is

$$\boldsymbol{\Sigma}=E\left( \mathbf{Y}\boldsymbol{-}\mathbf{X}\hat{\boldsymbol{g}} \right)\left( \mathbf{Y}\boldsymbol{-}\mathbf{X}\hat{\boldsymbol{g}} \right)^{\boldsymbol{T}}$$

$$=\boldsymbol{\Sigma}_{\boldsymbol{Y}}-E(\mathbf{X}\hat{\boldsymbol{g}}\mathbf{Y}^{\boldsymbol{T}}+\mathbf{Y}{\hat{\boldsymbol{g}}}^{\boldsymbol{T}}\mathbf{X}^{\boldsymbol{T}}-\mathbf{X}\hat{\boldsymbol{g}}{\hat{\boldsymbol{g}}}^{\boldsymbol{T}}\mathbf{X}^{\boldsymbol{T}})$$

where

$$E(\mathbf{X}\hat{\boldsymbol{g}}\mathbf{Y}^{\boldsymbol{T}})=E(\mathbf{Y}{\hat{\boldsymbol{g}}}^{\boldsymbol{T}}\mathbf{X}^{\boldsymbol{T}})=\left( \begin{matrix} \hat{d}\times\mathrm{Cov}\left( X_{m}^{'},Y_{1} \right)+\hat{f}\times\mathrm{Cov}(X_{f1}^{'},Y_{1}) & \hat{d}\times\mathrm{Cov}\left( X_{m}^{'},Y_{2} \right)+\hat{f}\times\mathrm{Cov}(X_{f1}^{'},Y_{2}) \\ \hat{d}\times\mathrm{Cov}\left( X_{m}^{'},Y_{1} \right)+\hat{f}\times\mathrm{Cov}(X_{f2}^{'},Y_{1}) & \hat{d}\times\mathrm{Cov}\left( X_{m}^{'},Y_{2} \right)+\hat{f}\times\mathrm{Cov}(X_{f2}^{'},Y_{2}) \end{matrix} \right)$$

$$E\left( \mathbf{X}\hat{\boldsymbol{g}}{\hat{\boldsymbol{g}}}^{\boldsymbol{T}}\mathbf{X}^{\boldsymbol{T}} \right)=\left( \begin{matrix} \hat{d}^{2}\times\mathrm{Var}\left( X_{m}^{'} \right)+2\hat{d}\hat{f}\times\mathrm{Cov}\left( X_{m}^{'},X_{f1}^{'} \right)+\hat{f}^{2}\times\mathrm{Var}(X_{f1}^{'}) & \hat{d}^{2}\times\mathrm{Var}\left( X_{m}^{'} \right)+2\hat{d}\hat{f}\times\mathrm{Cov}\left( X_{m}^{'},X_{f1}^{'} \right)+\hat{f}^{2}\times\mathrm{Cov}(X_{f1}^{'},X_{f2}^{'}) \\ \hat{d}^{2}\times\mathrm{Var}\left( X_{m}^{'} \right)+2\hat{d}\hat{f}\times\mathrm{Cov}\left( X_{m}^{'},X_{f2}^{'} \right)+\hat{f}^{2}\times\mathrm{Cov}(X_{f1}^{'},X_{f2}^{'}) & \hat{d}^{2}\times\mathrm{Var}\left( X_{m}^{'} \right)+2\hat{d}\hat{f}\times\mathrm{Cov}\left( X_{m}^{'},X_{f2}^{'} \right)+\hat{f}^{2}\times\mathrm{Var}(X_{f2}^{'}) \end{matrix} \right)$$

The expected -2lnL of the model per half sibling pair is:

$$E(-2lnL)=ln|\boldsymbol{\Sigma}|+2$$

Full omnibus model with imputed parental genotypes (terms for $X_{m}^{'}$,$X_{f}^{'}$, $X_{1}$ and $X_{2}$):

The **X** matrix contains three columns; column 1 with elements $X_{1}$ and $X_{2}$, column 2 with elements $X_{m}^{'}$ and $X_{m}^{'}$, and column 3 with elements $X_{f1}^{'}$ and $X_{f2}^{'}$. The asymptotic GLS estimate of the regression coefficients of columns 1, 2, and 3 are

$${\hat{\boldsymbol{g}}=(\hat{b},\hat{d}, \hat{f})}^{T}=E\left( \mathbf{X}^{\boldsymbol{T}}\boldsymbol{\Omega}^{\boldsymbol{-1}}\mathbf{X} \right)^{\boldsymbol{-1}}\mathbf{E}\left( \mathbf{X}^{\boldsymbol{T}}\boldsymbol{\Omega}^{\boldsymbol{-1}}\mathbf{Y} \right)$$

where

$$E\left( \mathbf{X}^{\boldsymbol{T}}\boldsymbol{\Omega}^{\boldsymbol{-1}}\mathbf{X} \right)^{\boldsymbol{-1}}=\left( \begin{matrix} \frac{\left( \sigma^{2}{+\varphi}^{2} \right)\left( \mathrm{Var}\left( X_{1} \right)+\mathrm{Var}\left( X_{2} \right) \right)-2\varphi^{2}\left( \mathrm{Cov}(X_{1},X_{2}) \right)}{\sigma^{2}(\sigma^{2}{+2\varphi}^{2})} & \frac{\mathrm{Cov}\left( X_{1},X_{m}^{'} \right)+\mathrm{Cov}\left( X_{2},X_{m}^{'} \right)}{\sigma^{2}{+2\varphi}^{2}} & \frac{\left( \sigma^{2}{+\varphi}^{2} \right)\left( \mathrm{Cov}(X_{f1}^{'},X_{1})+\mathrm{Cov}(X_{f2}^{'},X_{2}) \right)-\varphi^{2}\left( \mathrm{Cov}(X_{f1}^{'},X_{2})+\mathrm{Cov}(X_{f2}^{'},X_{1}) \right)}{\sigma^{2}(\sigma^{2}{+2\varphi}^{2})} \\ \frac{\mathrm{Cov}\left( X_{1},X_{m}^{'} \right)+\mathrm{Cov}\left( X_{2},X_{m}^{'} \right)}{\sigma^{2}{+2\varphi}^{2}} & \frac{2\times\mathrm{Var}(X_{m}^{'})}{\sigma^{2}{+2\varphi}^{2}} & \frac{2\times\mathrm{Cov}(X_{m}^{'},X_{f}^{'})}{\sigma^{2}{+2\varphi}^{2}} \\ \frac{\left( \sigma^{2}{+\varphi}^{2} \right)\left( \mathrm{Cov}(X_{f1}^{'},X_{1})+\mathrm{Cov}(X_{f2}^{'},X_{2}) \right)-\varphi^{2}\left( \mathrm{Cov}(X_{f1}^{'},X_{2})+\mathrm{Cov}(X_{f2}^{'},X_{1}) \right)}{\sigma^{2}(\sigma^{2}{+2\varphi}^{2})} & \frac{2\times\mathrm{Cov}(X_{m}^{'},X_{f}^{'})}{\sigma^{2}{+2\varphi}^{2}} & \frac{\left( \sigma^{2}{+\varphi}^{2} \right)\left( \mathrm{Var}(X_{f1}^{'})+\mathrm{Var}(X_{f2}^{'}) \right)-2\varphi^{2}\times Cov(X_{f1}^{'},X_{f2}^{'})}{\sigma^{2}(\sigma^{2}{+2\varphi}^{2})} \end{matrix} \right)^{-1}$$

$$E\left( \mathbf{X}^{\boldsymbol{T}}\boldsymbol{\Omega}^{\boldsymbol{-1}}\mathbf{Y} \right)=\left( \begin{matrix} \frac{\left( \sigma^{2}{+\varphi}^{2} \right)\left( \mathrm{Cov}\left( X_{1},Y_{1} \right)+\mathrm{Cov}\left( X_{2},Y_{2} \right) \right)-\varphi^{2}\left( \mathrm{Cov}\left( X_{1},Y_{2} \right)+\mathrm{Cov}\left( X_{2},Y_{1} \right) \right)}{\sigma^{2}(\sigma^{2}{+2\varphi}^{2})} \\ \frac{\mathrm{Cov}\left( Y_{1},X_{m}^{'} \right)+\mathrm{Cov}\left( Y_{2},X_{m}^{'} \right)}{\sigma^{2}{+2\varphi}^{2}} \\ \frac{\left( \sigma^{2}{+\varphi}^{2} \right)\left( \mathrm{Cov}(X_{f1}^{'},Y_{1})+\mathrm{Cov}(X_{f2}^{'},Y_{2}) \right)-\varphi^{2}\left( \mathrm{Cov}(X_{f1}^{'},Y_{2})+\mathrm{Cov}(X_{f2}^{'},Y_{1}) \right)}{\sigma^{2}(\sigma^{2}{+2\varphi}^{2})} \end{matrix} \right)$$

The residual covariance matrix is

$$\boldsymbol{\Sigma}=E\left( \mathbf{Y}-\mathbf{X}\hat{\boldsymbol{g}} \right)\left( \mathbf{Y}\boldsymbol{-}\mathbf{X}\hat{\boldsymbol{g}} \right)^{\boldsymbol{T}}$$

$$=\boldsymbol{\Sigma}_{\boldsymbol{Y}}-E(\mathbf{X}\hat{\boldsymbol{g}}\mathbf{Y}^{\boldsymbol{T}}+\mathbf{Y}{\hat{\boldsymbol{g}}}^{\boldsymbol{T}}\mathbf{X}^{\boldsymbol{T}}-\mathbf{X}\hat{\boldsymbol{g}}{\hat{\boldsymbol{g}}}^{\boldsymbol{T}}\mathbf{X}^{\boldsymbol{T}})$$

where

$$E(\mathbf{X}\hat{\boldsymbol{g}}\mathbf{Y}^{\boldsymbol{T}})=E(\mathbf{Y}{\hat{\boldsymbol{g}}}^{\boldsymbol{T}}\mathbf{X}^{\boldsymbol{T}})=\left( \begin{matrix} \hat{b}\times\mathrm{Cov}\left( X_{1},Y_{1} \right)+\hat{d}\times\mathrm{Cov}\left( X_{m}^{'},Y_{1} \right)+f\times\mathrm{Cov}(X_{f1}^{'},Y_{1}) & \hat{b}\times\mathrm{Cov}\left( X_{1},Y_{2} \right)+\hat{d}\times\mathrm{Cov}\left( X_{m}^{'},Y_{2} \right)+f\times\mathrm{Cov}(X_{f1}^{'},Y_{2}) \\ \hat{b}\times\mathrm{Cov}\left( X_{2},Y_{1} \right)+\hat{d}\times\mathrm{Cov}\left( X_{m}^{'},Y_{1} \right)+f\times\mathrm{Cov}(X_{f2}^{'},Y_{1}) & \hat{b}\times\mathrm{Cov}\left( X_{2},Y_{2} \right)+\hat{d}\times\mathrm{Cov}\left( X_{m}^{'},Y_{2} \right)+f\times\mathrm{Cov}(X_{f2}^{'},Y_{2}) \end{matrix} \right)$$

$$E\left( \mathbf{X}\hat{\boldsymbol{g}}{\hat{\boldsymbol{g}}}^{\boldsymbol{T}}\mathbf{X}^{\boldsymbol{T}} \right)=\left( \begin{matrix} \hat{b}^{2}+\hat{d}^{2}\mathrm{Var}\left( X_{m}^{'} \right)+\hat{f}^{2}\mathrm{Var}\left( X_{f}^{'} \right)+\hat{b}\hat{d}+\hat{b}\hat{f}+2\hat{d}\hat{f}\mathrm{Cov}\left( X_{m}^{'},X_{f}^{'} \right) & 0.25\hat{b}^{2}+\hat{d}^{2}\mathrm{Var}\left( X_{m}^{'} \right)+\hat{f}^{2}\mathrm{Cov}\left( X_{f1}^{'},X_{f2}^{'} \right)+\hat{b}\hat{d}+2\hat{d}\hat{f}\mathrm{Cov}\left( X_{m}^{'},X_{f}^{'} \right) \\ 0.25\hat{b}^{2}+\hat{d}^{2}\mathrm{Var}\left( X_{m}^{'} \right)+\hat{f}^{2}\mathrm{Cov}\left( X_{f1}^{'},X_{f2}^{'} \right)+\hat{b}\hat{d}+2\hat{d}\hat{f}\mathrm{Cov}\left( X_{m}^{'},X_{f}^{'} \right) & \hat{b}^{2}+\hat{d}^{2}\mathrm{Var}\left( X_{m}^{'} \right)+\hat{f}^{2}\mathrm{Var}\left( X_{f}^{'} \right)+\hat{b}\hat{d}+\hat{b}\hat{f}+2\hat{d}\hat{f}\mathrm{Cov}\left( X_{m}^{'},X_{f}^{'} \right) \end{matrix} \right)$$

The expected -2lnL of the model per half sibling pair is:

$$E(-2lnL)=ln|\boldsymbol{\Sigma}|+2$$

**Sibling Pairs at X Chromosome loci**

We assume that unstandardized male genotypes are coded *G_1_* $\in$ {0,2} and female genotypes coded as *G_2_* $\in$ {0,1 or 2}. This means that males have double the variance of females at non-pseudoautosomal X-linked loci. We assume that the regression coefficient of offspring phenotype on offspring genotype is the same in males and females. We parameterize our model so that X chromosome genotypes are standardized relative to the female genotypic variance, such that female siblings and mothers have unit variance, while male siblings and fathers have a variance equal to two. We also assume that male and female phenotypes are standardized. As covariances between sib pair genotypes are sex-dependent, three separate models must be constructed for female-female, male-male, and female-male sib pairs.

The causal model for a sib-pair where sibling one is male and sibling two is female is then

$$Y_{1i}=bX_{1i}+dX_{m,i}+fX_{f,i}+\tau+\varepsilon_{1i}$$

$$Y_{2i}=bX_{2i}+dX_{m,i}+fX_{f,i}+\tau+\varepsilon_{2i}$$

$$X_{1i}=X_{m,i}+\eta_{Mi}$$

$$X_{2i}=0.5X_{m,i}+{0.5X}_{f,i}+\eta_{Fi}$$

where *τ* is a random effect shared by the siblings, *ε*_1_ and *ε*_2_ are uncorrelated error terms for the two phenotypes, and *η*_M_ and *η*_F_ are random effects due to segregation. The variances of the random effects are

$$\mathrm{Var}\left( \varepsilon_{1} \right)=\sigma_{\varepsilon1}^{2}$$

$$\mathrm{Var}\left( \varepsilon_{2} \right)=\sigma_{\varepsilon2}^{2}$$

$$\mathrm{Var}\left( \tau\right)=\varphi^{2}$$

$$\mathrm{Var}\left( \eta_{M} \right)=1$$

$$\mathrm{Var}\left( \eta_{F} \right)=0.25$$

where $\mathrm{Var}\left( \varepsilon_{1} \right)=\mathrm{Var}\left( \varepsilon_{2} \right)=\sigma^{2}$ in the case of same sex siblings.

**Covariances**

We assume random mating, so that Cov(X_m_,X_f_) = 0

So that the covariances between genotypes are

Mother-Daughter

Cov(*X*_2_,*X*_m_) = 0.5

Mother-Son

Cov(*X*_1_,*X*_m_) = 1

Father-Daughter

Cov(*X*_2_,*X*_f_) = 1

Father-Son

Cov(*X*_1_,*X*_f_) = 0

Brother-Brother

Cov(*X*_1_,*X*_2_) = 1

Sister-Sister

Cov(*X*_1_,*X*_2_) = 0.75

Brother-Sister

Cov(*X*_1_,*X*_2_) = 0.5

The covariances between phenotypes and genotypes for the following sibling pair combinations are then:

Female-Female

Cov(*Y*_1,_*X*_1_) = Cov(*Y*_2_,*X*_2_) = $b+0.5d+f$

Cov(*Y*_1_,*X*_2_) = Cov(*Y*_2_,*X*_1_) = $0.75b+0.5d+f$

Cov(*Y*_1_,*X*_m_) = Cov(*Y*_2_,*X*_m_) =$0.5b+d$

Cov(*Y*_1_,*X*_f_) = Cov(*Y*_2_,*X*_f_) = $b+2f$

Male-Male

Cov(*Y*_1,_*X*_1_) = Cov(*Y*_2_,*X*_2_) = $2b+d$

Cov(*Y*_1_,*X*_2_) = Cov(*Y*_2_,*X*_1_) = $b+d$

Cov(*Y*_1_,*X*_m_) = Cov(*Y*_2_,*X*_m_) = $b+d$

Cov(*Y*_1_,*X*_f_) = Cov(*Y*_2_,*X*_f_) = $2f$

Male-Female

Cov(*Y*_1,_*X*_1_) = $2b+d$

Cov(*Y*_2_,*X*_2_) = $b+0.5d+f$

Cov(*Y*_1_,*X*_2_) = $0.5\left( b+d \right)+f$

Cov(*Y*_2_,*X*_1_) = $\left( 0.5b+d \right)$

Cov(*Y*_1_,*X*_m_) = $b+d$

Cov(*Y*_2_,*X*_m_) = $0.5b+d$

Cov(*Y*_1_,*X*_f_) = $2f$

Cov(*Y*_2_,*X*_f_) = $b+2f$

The covariance of the two phenotypes is

Female-Female

Cov*(Y_1_,Y_2_) =* $0.75b^{2}+d^{2}+{2f}^{2}+bd+2bf+\varphi^{2}$

Male-Male

Cov*(Y_1_,Y_2_) =* $b^{2}+d^{2}+{2f}^{2}+2bd+\varphi^{2}$

Male-Female

Cov*(Y_1_,Y_2_) =* $0.5b^{2}+d^{2}+{2f}^{2}+1.5bd+bf+\varphi^{2}$

The phenotypic variance of offspring can be decomposed as follows

Male

$$\mathrm{Var}\left( Y \right)=\left( 2b^{2}+d^{2}+2f^{2}+2bd \right)+\varphi^{2}+\sigma_{\varepsilon1}^{2}=1$$

Female

$$\mathrm{Var}\left( Y \right)=\left( b^{2}+d^{2}+{2f}^{2}+bd+2bf \right)+\varphi^{2}+\sigma_{\varepsilon2}^{2}=1$$

The variance of the imputed maternal genotype, $\mathrm{Var}(X_{m}^{'})$, is a function of p or the expected female heterozygosity *H* such that for the following sib-pairs

Female-Female

$$\mathrm{Var}\left( X_{m}^{'} \right)=\frac{2H^{2}+4H-7}{-3H-12}$$

Male-Male

$$\mathrm{Var}\left( X_{m}^{'} \right)=\frac{3}{H+4}$$

Male-Female

$$\mathrm{Var}\left( X_{m}^{'} \right)=\frac{2H^{2}+6H+7}{(2H+3)(H+4)}$$

The variance of the imputed paternal genotype, $\mathrm{Var}(X_{f}^{'})$, is a function of the male heterozygote frequency $H_{f}$ $H_{f}= 4pq$

Female-Female

$$\mathrm{Var}\left( X_{f}^{'} \right)=\frac{16-H_{f}}{12}$$

Male-Female

$$\mathrm{Var}\left( X_{f}^{'} \right)=\frac{H_{f}+4}{(H_{f}+3)}$$

Similarly, the covariance between imputed maternal and paternal genotypes are a function of the expected female heterozygosity *H*:

Female-Female:

$$\mathrm{Cov}\left( X_{m}^{'},X_{f}^{'} \right)=\frac{\sqrt{2}(1+H)}{6}$$

Male-Female:

$$\mathrm{Cov}\left( X_{m}^{'},X_{f}^{'} \right)=\frac{\sqrt{2}}{2(3+2H)}$$

and the covariance between imputed maternal or paternal genotypes and a given child’s phenotype *Y* are

for female offspring:

$$\mathrm{Cov}\left( X_{m}^{'},Y \right)=0.5b+d\times\mathrm{Var}\left( X_{m}^{'} \right)+f\times\mathrm{Cov}\left( X_{m}^{'},X_{f}^{'} \right)$$

$$\mathrm{Cov}\left( X_{f}^{'},Y \right)=b+d\times\mathrm{Cov}\left( X_{m}^{'},X_{f}^{'} \right)+f\times\mathrm{Var}(X_{f}^{'})$$

for male offspring:

$$\mathrm{Cov}\left( X_{m}^{'},Y \right)=b+d\times\mathrm{Var}\left( X_{m}^{'} \right)+f\times\mathrm{Cov}\left( X_{m}^{'},X_{f}^{'} \right)$$

$$\mathrm{Cov}\left( X_{f}^{'},Y \right)=d\times\mathrm{Cov}\left( X_{m}^{'},X_{f}^{'} \right)+f\times\mathrm{Var}(X_{f}^{'})$$

**Power calculation under linear mixed model analysis for X chromosome loci**

When actual maternal and paternal genotypes are available, the linear mixed model is

$$Y_{1i}=bX_{1i}+dX_{m,i}+fX_{f,i}+\tau_{i}+\varepsilon_{1i}$$

$$Y_{2i}=bX_{2i}+dX_{m,i}+fX_{f,i}+\tau_{i}+\varepsilon_{2i}$$

The fixed effects *b*, *d,* and *f* are estimated by generalised least squares (GLS), where the covariance matrix of random effects is:

$$\boldsymbol{\Omega}=\left( \begin{matrix} \sigma^{2}{+\varphi}^{2} & \varphi^{2} \\ \varphi^{2} & \sigma^{2}{+\varphi}^{2} \end{matrix} \right)$$

The inverse of the covariance matrix of random effects is

$$\boldsymbol{\Omega}^{\boldsymbol{-1}}=\frac{1}{\sigma^{2}(\sigma^{2}{+2\varphi}^{2})}\left( \begin{matrix} \sigma^{2}{+\varphi}^{2} & {-\varphi}^{2} \\ {-\varphi}^{2} & \sigma^{2}{+\varphi}^{2} \end{matrix} \right)$$

The asymptotic GLS estimates of a vector of parameters $\boldsymbol{\beta}$ are given by

$$\hat{\boldsymbol{\beta}}={E\left( \mathbf{X}^{\boldsymbol{T}}\boldsymbol{\Omega}^{\boldsymbol{-1}}\mathbf{X} \right)}^{\boldsymbol{-1}}E\left( \mathbf{X}^{\boldsymbol{T}}\boldsymbol{\Omega}^{\boldsymbol{-1}}\mathbf{Y} \right)$$

We consider the following models for each possible sibling pair combination:

Null model of no association

The residual covariance matrix is simply the covariance matrix of Y:

Female-Female

$$\boldsymbol{\Sigma}=\boldsymbol{\Sigma}_{\boldsymbol{Y}}=\left( \begin{matrix} 1 & 0.75b^{2}+d^{2}+{2f}^{2}+bd+2bf+\varphi^{2} \\ 0.75b^{2}+d^{2}+{2f}^{2}+bd+2bf+\varphi^{2} & 1 \end{matrix} \right)$$

Male-Male

$$\boldsymbol{\Sigma}=\boldsymbol{\Sigma}_{\boldsymbol{Y}}=\left( \begin{matrix} 1 & b^{2}+d^{2}+{2f}^{2}+2bd+\varphi^{2} \\ b^{2}+d^{2}+{2f}^{2}+2bd+\varphi^{2} & 1 \end{matrix} \right)$$

Female-Male

$$\boldsymbol{\Sigma}=\boldsymbol{\Sigma}_{\boldsymbol{Y}}=\left( \begin{matrix} 1 & 0.5b^{2}+d^{2}+{2f}^{2}+1.5bd+bf+\varphi^{2} \\ 0.5b^{2}+d^{2}+{2f}^{2}+1.5bd+bf+\varphi^{2} & 1 \end{matrix} \right)$$

The expected -2lnL of the model per sibling pair is therefore

Female-Female:

$$E(-2lnL)={ln(1-\left( 0.75b^{2}+d^{2}+{2f}^{2}+bd+2bf+\varphi^{2} \right)}^{2})+2$$

Male-Male:

$$E(-2lnL)=ln({1-\left( b^{2}+d^{2}+{2f}^{2}+2bd+\varphi^{2} \right)}^{2})+2$$

Female-Male:

$$E(-2lnL)={ln(1-\left( 0.5b^{2}+d^{2}+{2f}^{2}+1.5bd+bf+\varphi^{2} \right)}^{2})+2$$

Both parental genotypes only in model (terms for $X_{m}$ and $X_{f}$ only):

The **X** matrix contains two columns with elements $X_{m}$ and $X_{f}$. The asymptotic GLS estimates for the regression coefficients of $X_{m}$ and $X_{f}$ for a sibling pair are

$$\hat{\boldsymbol{g}}=\left( \hat{d},\hat{f} \right)^{T}=E\left( \mathbf{X}^{\boldsymbol{T}}\boldsymbol{\Omega}^{\boldsymbol{-1}}\mathbf{X} \right)^{-1}E\left( \mathbf{X}^{\boldsymbol{T}}\boldsymbol{\Omega}^{\boldsymbol{-1}}\mathbf{Y} \right)$$

$$=E\left( \begin{matrix} \frac{2X_{m}^{2}}{\sigma^{2}{+2\varphi}^{2}} & \frac{2X_{m}X_{f}}{\sigma^{2}{+2\varphi}^{2}} \\ \frac{2X_{m}X_{f}}{\sigma^{2}{+2\varphi}^{2}} & \frac{2X_{f}^{2}}{\sigma^{2}{+2\varphi}^{2}} \end{matrix} \right)^{-1}E\left( \begin{matrix} \frac{X_{m}\left( Y_{1}+Y_{2} \right)}{\sigma^{2}{+2\varphi}^{2}} \\ \frac{X_{f}\left( Y_{1}+Y_{2} \right)}{\sigma^{2}{+2\varphi}^{2}} \end{matrix} \right)$$

$$=\left( \begin{matrix} \frac{\mathrm{Cov}\left( X_{m},Y_{1} \right)+\mathrm{Cov}\left( X_{m},Y_{2} \right)}{2} & \frac{\mathrm{Cov}\left( X_{f},Y_{1} \right)+\mathrm{Cov}\left( X_{f},Y_{2} \right)}{4} \end{matrix} \right)^{T}$$

Thus, the $\hat{\boldsymbol{g}}$ matrix for each of the following sib pairs are

Female-Female

$$\hat{\boldsymbol{g}}=\left( \begin{matrix} 0.5b+d & 0.5b+f \end{matrix} \right)^{T}$$

Male-Male

$$\hat{\boldsymbol{g}}=\left( \begin{matrix} b+d & f \end{matrix} \right)^{T}$$

Female-Male

$$\hat{\boldsymbol{g}}=\left( \begin{matrix} 0.5b+d & 0.25b+f \end{matrix} \right)^{T}$$

The residual covariance matrix is

$$\boldsymbol{\Sigma}=E\left( \mathbf{Y}-\mathbf{X}\hat{\boldsymbol{g}} \right)\left( \mathbf{Y}\boldsymbol{-}\mathbf{X}\hat{\boldsymbol{g}} \right)^{\boldsymbol{T}}$$

$$=\boldsymbol{\Sigma}_{\boldsymbol{Y}}-E\boldsymbol{(}\mathbf{X}\hat{\boldsymbol{g}}\mathbf{Y}^{\boldsymbol{T}}+\mathbf{Y}{\hat{\boldsymbol{g}}}^{\boldsymbol{T}}\mathbf{X}^{\boldsymbol{T}}-\mathbf{X}\hat{\boldsymbol{g}}{\hat{\boldsymbol{g}}}^{\boldsymbol{T}}\mathbf{X}^{\boldsymbol{T}})$$

where

$$E(\mathbf{X}\hat{\boldsymbol{g}}\mathbf{Y}^{\boldsymbol{T}})=E(\mathbf{Y}{\hat{\boldsymbol{g}}}^{\boldsymbol{T}}\mathbf{X}^{\boldsymbol{T}})=\left( \begin{matrix} \hat{d}\times Cov\left( X_{m},Y_{1} \right)+\hat{f}\times Cov(X_{f},Y_{1}) & \hat{d}\times Cov\left( X_{m},Y_{2} \right)+\hat{f}\times Cov(X_{f},Y_{2}) \\ \hat{d}\times Cov\left( X_{m},Y_{1} \right)+\hat{f}\times Cov(X_{f},Y_{1}) & \hat{d}\times Cov\left( X_{m},Y_{2} \right)+\hat{f}\times Cov(X_{f},Y_{2}) \end{matrix} \right)$$

$$E\left( \mathbf{X}\hat{\boldsymbol{g}}{\hat{\boldsymbol{g}}}^{\boldsymbol{T}}\mathbf{X}^{\boldsymbol{T}} \right)=\left( \begin{matrix} \hat{d}^{2}\mathrm{Var}\left( X_{m} \right)+2\hat{d}\hat{f}\mathrm{Cov}\left( X_{m},X_{f} \right)+\hat{f}^{2}\mathrm{Var}(X_{f}) & \hat{d}^{2}\mathrm{Var}\left( X_{m} \right)+2\hat{d}\hat{f}\mathrm{Cov}\left( X_{m},X_{f} \right)+\hat{f}^{2}\mathrm{Var}(X_{f}) \\ \hat{d}^{2}\mathrm{Var}\left( X_{m} \right)+2\hat{d}\hat{f}\mathrm{Cov}\left( X_{m},X_{f} \right)+\hat{f}^{2}\mathrm{Var}(X_{f}) & \hat{d}^{2}\mathrm{Var}\left( X_{m} \right)+2\hat{d}\hat{f}\mathrm{Cov}\left( X_{m},X_{f} \right)+\hat{f}^{2}\mathrm{Var}(X_{f}) \end{matrix} \right)$$

The expected -2lnL of the model per sibling pair is

$$E(-2lnL)=ln|\boldsymbol{\Sigma}|+2$$

Offspring genotypes in model only (terms for $X_{1}$ and $X_{2}$ only):

The **X** matrix contains one column with elements $X_{1}$ and $X_{2}$. The asymptotic GLS estimate of the regression coefficient of $X_{1}$ and $X_{2}$ is

$$\hat{b}=E\left( \mathbf{X}^{\boldsymbol{T}}\boldsymbol{\Omega}^{\boldsymbol{-1}}\mathbf{X} \right)^{\boldsymbol{-1}}E\left( \mathbf{X}^{\boldsymbol{T}}\boldsymbol{\Omega}^{\boldsymbol{-1}}\mathbf{Y} \right)$$

$$=E\left( \left( \sigma^{2}+\varphi^{2} \right)\left( X_{1}^{2}+X_{2}^{2} \right)-2X_{1}X_{2}\varphi^{2} \right)^{-1}E\left( \left( \sigma^{2}+\varphi^{2} \right)\left( X_{1}Y_{1}+X_{2}Y_{2} \right)-\varphi^{2}\left( X_{1}Y_{2}+X_{2}Y_{1} \right) \right)$$

$$=\frac{(\sigma^{2}+\varphi^{2})\left( \mathrm{Cov}\left( X_{1},Y_{1} \right)+\mathrm{Cov}\left( X_{2},Y_{2} \right) \right)-\varphi^{2}\left( \mathrm{Cov}\left( X_{1},Y_{2} \right)+\mathrm{Cov}\left( X_{2},Y_{1} \right) \right)}{\left( \sigma^{2}+\varphi^{2} \right)\left( \mathrm{Var}\left( X_{1} \right)+\mathrm{Var}\left( X_{2} \right) \right)-2\varphi^{2}\left( \mathrm{Cov}\left( X_{1},X_{2} \right) \right)}$$

Thus, for each of the following sib-pair combinations,

Female-Female:

$$\hat{b}=b+\frac{\sigma^{2}\left( d+f \right)}{2\sigma^{2}+0.5\varphi^{2}}$$

Male-Male:

$$\hat{b}=b+\frac{\sigma^{2}d}{2\sigma^{2}+\varphi^{2}}$$

Female-Male:

$$\hat{b}=b+\frac{d\left( 2\sigma_{1}^{2}+\sigma_{2}^{2} \right)+2f\sigma_{2}^{2}}{2(2\sigma_{1}^{2}+\sigma_{2}^{2}+2\varphi^{2})}$$

The residual covariance matrix is

$$\boldsymbol{\Sigma}=E\left( \mathbf{Y}-\mathbf{X}\hat{b} \right)\left( \mathbf{Y}-\mathbf{X}\hat{b} \right)^{T}$$

$$=\boldsymbol{\Sigma}_{\mathbf{Y}}-\hat{b}\left( \boldsymbol{\Sigma}_{\boldsymbol{YX}}+\boldsymbol{\Sigma}_{\boldsymbol{XY}} \right)+\hat{b}^{2}\boldsymbol{\Sigma}_{\boldsymbol{X}}$$

Where for the following sib-pair combinations,

Female-Female:

$$\boldsymbol{\Sigma}_{\boldsymbol{YX}}=\boldsymbol{\Sigma}_{\boldsymbol{XY}}=\left( \begin{matrix} b+0.5d+f & 0.75b+0.5d+f \\ 0.75b+0.5d+f & b+0.5d+f \end{matrix} \right)$$

$$\boldsymbol{\Sigma}_{\boldsymbol{X}}=\left( \begin{matrix} 1 & 0.75 \\ 0.75 & 1 \end{matrix} \right)$$

Male-Male:

$$\boldsymbol{\Sigma}_{\boldsymbol{YX}}=\boldsymbol{\Sigma}_{\boldsymbol{XY}}=\left( \begin{matrix} 2b+d & b+d \\ b+d & 2b+d \end{matrix} \right)$$

$$\boldsymbol{\Sigma}_{\boldsymbol{X}}=\left( \begin{matrix} 2 & 1 \\ 1 & 2 \end{matrix} \right)$$

Female-Male:

$$\boldsymbol{\Sigma}_{\boldsymbol{YX}}=\left( \begin{matrix} b+0.5d+f & 0.5b+d \\ 0.5b+0.5d+f & 2b+d \end{matrix} \right)$$

$$\boldsymbol{\Sigma}_{\boldsymbol{XY}}=\left( \begin{matrix} b+0.5d+f & 0.5\left( b+d \right)+f \\ \left( 0.5b+d \right) & 2b+d \end{matrix} \right)$$

$$\boldsymbol{\Sigma}_{\boldsymbol{X}}=\left( \begin{matrix} 1 & 0.5 \\ 0.5 & 2 \end{matrix} \right)$$

and $\boldsymbol{\Sigma}_{\boldsymbol{Y}}$ is defined as above for each sibling pair combination

Full Omnibus Model (terms for $X_{m}$, $X_{f}$, $X_{1}$ and $X_{2}$):

The **X** matrix contains three columns; column 1 with elements $X_{1}$ and $X_{2}$, column 2 with elements $X_{m}$ and $X_{m}$, and column 3 with elements $X_{f}$ and $X_{f}$. The asymptotic GLS estimate of the regression coefficients of columns 1, 2, and 3 are

$$\hat{\boldsymbol{g}}={(\hat{b},\hat{d}, \hat{f})}^{T}=E\left( \mathbf{X}^{\boldsymbol{T}}\boldsymbol{\Omega}^{\boldsymbol{-1}}\mathbf{X} \right)^{\boldsymbol{-1}}E\left( \mathbf{X}^{\boldsymbol{T}}\boldsymbol{\Omega}^{\boldsymbol{-1}}\mathbf{Y} \right)$$

$$={(b,d,f)}^{T}$$

The residual covariance matrix is

$$\boldsymbol{\Sigma}=E\left( \mathbf{Y}-\mathbf{X}\hat{\boldsymbol{g}} \right)\left( \mathbf{Y}\boldsymbol{-}\mathbf{X}\hat{\boldsymbol{g}} \right)^{\boldsymbol{T}}$$

$$=\boldsymbol{\Omega}$$

$$=\left( \begin{matrix} \sigma^{2}{+\varphi}^{2} & \varphi^{2} \\ \varphi^{2} & \sigma^{2}{+\varphi}^{2} \end{matrix} \right)$$

The expected -2lnL of the model per sibling pair is

$$E(-2lnL)=ln|\boldsymbol{\Sigma}|+2$$

Imputed parental genotypes only in model (terms for $X_{m}^{'}$ and $X_{f}^{'}$only)

When only imputed maternal genotypes are available, the linear mixed model becomes

$$Y_{1i}=bX_{1i}+dX_{m,i}^{'}+fX_{f,i}^{'}+\tau_{i}+\varepsilon_{1i}$$

$$Y_{2i}=bX_{2i}+dX_{m,i}^{'}+fX_{f,i}^{'}+\tau_{i}+\varepsilon_{2i}$$

The **X** matrix contains two columns, one with elements $X_{m}^{'}$ and another with elements $X_{f}^{'}$. The asymptotic GLS estimate of the regression coefficient of $X_{m}^{'}$ and $X_{f}^{'}$ are

$$\hat{\boldsymbol{g}}=\left( \hat{d},\hat{f} \right)^{T}=E\left( \mathbf{X}^{\boldsymbol{T}}\boldsymbol{\Omega}^{\boldsymbol{-1}}\mathbf{X} \right)^{\boldsymbol{-1}}E\left( \mathbf{X}^{\boldsymbol{T}}\boldsymbol{\Omega}^{\boldsymbol{-1}}\mathbf{Y} \right)$$

$$=E\left( \begin{matrix} \frac{2{X_{m}^{'}}^{2}}{\sigma^{2}{+2\varphi}^{2}} & \frac{2X_{m}^{'}X_{f}^{'}}{\sigma^{2}{+2\varphi}^{2}} \\ \frac{2X_{m}'X_{f}'}{\sigma^{2}{+2\varphi}^{2}} & \frac{2{X_{f}^{'}}^{2}}{\sigma^{2}{+2\varphi}^{2}} \end{matrix} \right)^{-1}E\left( \begin{matrix} \frac{X_{m}^{'}\left( Y_{1}+Y_{2} \right)}{\sigma^{2}{+2\varphi}^{2}} \\ \frac{X_{f}^{'}\left( Y_{1}+Y_{2} \right)}{\sigma^{2}{+2\varphi}^{2}} \end{matrix} \right)$$

$$=\left( \begin{matrix} \frac{\mathrm{Var}(X_{f}^{'})\left( \mathrm{Cov}\left( X_{m}^{'},Y_{1} \right)+\mathrm{Cov}\left( X_{m}^{'},Y_{2} \right) \right)-\mathrm{Cov}(X_{m}^{'},X_{f}^{'})\left( \mathrm{Cov}\left( X_{f}^{'},Y_{1} \right)+\mathrm{Cov}\left( X_{f}^{'},Y_{2} \right) \right)}{2\mathrm{Var}\left( X_{m}^{'} \right)2\mathrm{Var}\left( X_{f}^{'} \right)-{2\mathrm{Cov}(X_{m}^{'},X_{f}^{'})}^{2}} \\ \frac{\mathrm{Var}(X_{m}^{'})\left( \mathrm{Cov}\left( X_{f}^{'},Y_{1} \right)+\mathrm{Cov}\left( X_{f}^{'},Y_{2} \right) \right)-\mathrm{Cov}(X_{m}^{'},X_{f}^{'})\left( \mathrm{Cov}\left( X_{m}^{'},Y_{1} \right)+\mathrm{Cov}\left( X_{m}^{'},Y_{2} \right) \right)}{2\mathrm{Var}\left( X_{m}^{'} \right)2\mathrm{Var}\left( X_{f}^{'} \right)-{2\mathrm{Cov}(X_{m}^{'},X_{f}^{'})}^{2}} \end{matrix} \right)$$

The residual covariance matrix is

$$\boldsymbol{\Sigma}=E\left( \mathbf{Y}-\mathbf{X}\hat{\boldsymbol{g}} \right)\left( \mathbf{Y}\boldsymbol{-}\mathbf{X}\hat{\boldsymbol{g}} \right)^{\boldsymbol{T}}$$

$$=\boldsymbol{\Sigma}_{\boldsymbol{Y}}-E(\mathbf{X}\hat{\boldsymbol{g}}\mathbf{Y}^{\boldsymbol{T}}+\mathbf{Y}{\hat{\boldsymbol{g}}}^{\boldsymbol{T}}\mathbf{X}^{\boldsymbol{T}}-\mathbf{X}\hat{\boldsymbol{g}}{\hat{\boldsymbol{g}}}^{\boldsymbol{T}}\mathbf{X}^{\boldsymbol{T}})$$

where

$$E(\mathbf{X}\hat{\boldsymbol{g}}\mathbf{Y}^{\boldsymbol{T}})=E(\mathbf{Y}{\hat{\boldsymbol{g}}}^{\boldsymbol{T}}\mathbf{X}^{\boldsymbol{T}})=\left( \begin{matrix} \hat{d}\times Cov\left( X_{m}^{'},Y_{1} \right)+\hat{f}\times Cov(X_{f}^{'},Y_{1}) & \hat{d}\times Cov\left( X_{m}^{'},Y_{2} \right)+\hat{f}\times Cov(X_{f}^{'},Y_{2}) \\ \hat{d}\times Cov\left( X_{m}^{'},Y_{1} \right)+\hat{f}\times Cov(X_{f}^{'},Y_{1}) & \hat{d}\times Cov\left( X_{m}^{'},Y_{2} \right)+\hat{f}\times Cov(X_{f}^{'},Y_{2}) \end{matrix} \right)$$

$$E\left( \mathbf{X}\hat{\boldsymbol{g}}{\hat{\boldsymbol{g}}}^{\boldsymbol{T}}\mathbf{X}^{\boldsymbol{T}} \right)=\left( \begin{matrix} \hat{d}^{2}\mathrm{Var}\left( X_{m}^{'} \right)+2\hat{d}\hat{f}\mathrm{Cov}\left( X_{m}^{'},X_{f}^{'} \right)+\hat{f}^{2}\mathrm{Var}(X_{f}^{'}) & \hat{d}^{2}\mathrm{Var}\left( X_{m}^{'} \right)+2\hat{d}\hat{f}\mathrm{Cov}\left( X_{m}^{'},X_{f}^{'} \right)+\hat{f}^{2}\mathrm{Var}(X_{f}^{'}) \\ \hat{d}^{2}\mathrm{Var}\left( X_{m}^{'} \right)+2\hat{d}\hat{f}\mathrm{Cov}\left( X_{m}^{'},X_{f}^{'} \right)+\hat{f}^{2}\mathrm{Var}(X_{f}^{'}) & \hat{d}^{2}\mathrm{Var}\left( X_{m}^{'} \right)+2\hat{d}\hat{f}\mathrm{Cov}\left( X_{m}^{'},X_{f}^{'} \right)+\hat{f}^{2}\mathrm{Var}(X_{f}^{'}) \end{matrix} \right)$$

The expected -2lnL of the model per sibling pair is

$$E(-2lnL)=ln|\boldsymbol{\Sigma}|+2$$

Full omnibus model with imputed parental genotypes (terms for $X_{m}^{'}$, $X_{f}^{'}$, $X_{1}$ and $X_{2}$):

The **X** matrix contains three columns; column 1 with elements $X_{1}$ and $X_{2}$, column 2 with elements $X_{m}^{'}$ and $X_{m}^{'}$, and column 3 with elements $X_{f}^{'}$ and $X_{f}^{'}$. The asymptotic GLS estimate of the regression coefficients of columns 1, 2, and 3 are

$${\hat{\boldsymbol{g}}=(\hat{b},\hat{d}, \hat{f})}^{T}=E\left( \mathbf{X}^{\boldsymbol{T}}\boldsymbol{\Omega}^{\boldsymbol{-1}}\mathbf{X} \right)^{\boldsymbol{-1}}E\left( \mathbf{X}^{\boldsymbol{T}}\boldsymbol{\Omega}^{\boldsymbol{-1}}\mathbf{Y} \right)$$

where

$$E\left( \mathbf{X}^{\boldsymbol{T}}\boldsymbol{\Omega}^{\boldsymbol{-1}}\mathbf{X} \right)^{\boldsymbol{-1}}=\left( \begin{matrix} \frac{\left( \sigma_{1}^{2}{+\varphi}^{2} \right)\mathrm{Var}\left( X_{1} \right)\left( \sigma_{2}^{2}{+\varphi}^{2} \right)\mathrm{Var}\left( X_{2} \right)-2\varphi^{2}\left( \mathrm{Cov}(X_{1},X_{2}) \right)}{\sigma_{1}^{2}\sigma_{2}^{2}{+\varphi}^{2}(\sigma_{1}^{2}+\sigma_{2}^{2})} & \frac{\sigma_{1}^{2}\mathrm{Cov}\left( X_{1},X_{m}^{'} \right)+\sigma_{2}^{2}\mathrm{Cov}\left( X_{2},X_{m}^{'} \right)}{\sigma_{1}^{2}\sigma_{2}^{2}{+\varphi}^{2}(\sigma_{1}^{2}+\sigma_{2}^{2})} & \frac{\sigma_{1}^{2}\mathrm{Cov}\left( X_{1},X_{f}^{'} \right)+\sigma_{2}^{2}\mathrm{Cov}\left( X_{2},X_{f}^{'} \right)}{\sigma_{1}^{2}\sigma_{2}^{2}{+\varphi}^{2}(\sigma_{1}^{2}+\sigma_{2}^{2})} \\ \frac{\sigma_{1}^{2}\mathrm{Cov}\left( X_{1},X_{m}^{'} \right)+\sigma_{2}^{2}\mathrm{Cov}\left( X_{2},X_{m}^{'} \right)}{\sigma_{1}^{2}\sigma_{2}^{2}{+\varphi}^{2}(\sigma_{1}^{2}+\sigma_{2}^{2})} & \frac{(\sigma_{1}^{2}+\sigma_{2}^{2})\mathrm{Var}(X_{m}^{'})}{\sigma_{1}^{2}\sigma_{2}^{2}{+\varphi}^{2}(\sigma_{1}^{2}+\sigma_{2}^{2})} & \frac{(\sigma_{1}^{2}+\sigma_{2}^{2})\mathrm{Cov}(X_{m}^{'},X_{f}^{'})}{\sigma_{1}^{2}\sigma_{2}^{2}{+\varphi}^{2}(\sigma_{1}^{2}+\sigma_{2}^{2})} \\ \frac{\sigma_{1}^{2}\mathrm{Cov}\left( X_{1},X_{f}^{'} \right)+\sigma_{2}^{2}\mathrm{Cov}\left( X_{2},X_{f}^{'} \right)}{\sigma_{1}^{2}\sigma_{2}^{2}{+\varphi}^{2}(\sigma_{1}^{2}+\sigma_{2}^{2})} & \frac{(\sigma_{1}^{2}+\sigma_{2}^{2})\mathrm{Cov}(X_{m}^{'},X_{f}^{'})}{\sigma_{1}^{2}\sigma_{2}^{2}{+\varphi}^{2}(\sigma_{1}^{2}+\sigma_{2}^{2})} & \frac{(\sigma_{1}^{2}+\sigma_{2}^{2})\mathrm{Var}(X_{f}^{'})}{\sigma_{1}^{2}\sigma_{2}^{2}{+\varphi}^{2}(\sigma_{1}^{2}+\sigma_{2}^{2})} \end{matrix} \right)^{-1}$$

$$E\left( \mathbf{X}^{\boldsymbol{T}}\boldsymbol{\Omega}^{\boldsymbol{-1}}\mathbf{Y} \right)=\left( \begin{matrix} \frac{\left( \sigma_{1}^{2}{+\varphi}^{2} \right)\mathrm{Cov}\left( X_{1},Y_{1} \right)+\left( \sigma_{1}^{2}{+\varphi}^{2} \right)\mathrm{Cov}\left( X_{2},Y_{2} \right)-\varphi^{2}\left( \mathrm{Cov}\left( X_{1},Y_{2} \right)+\mathrm{Cov}\left( X_{2},Y_{1} \right) \right)}{\sigma_{1}^{2}\sigma_{2}^{2}{+\varphi}^{2}(\sigma_{1}^{2}+\sigma_{2}^{2})} \\ \frac{\sigma_{1}^{2}\mathrm{Cov}\left( Y_{1},X_{m}^{'} \right)+\sigma_{2}^{2}\mathrm{Cov}\left( Y_{2},X_{m}^{'} \right)}{\sigma_{1}^{2}\sigma_{2}^{2}{+\varphi}^{2}(\sigma_{1}^{2}+\sigma_{2}^{2})} \\ \frac{\sigma_{1}^{2}\mathrm{Cov}\left( Y_{1},X_{f}^{'} \right)+\sigma_{2}^{2}\mathrm{Cov}\left( Y_{2},X_{f}^{'} \right)}{\sigma_{1}^{2}\sigma_{2}^{2}{+\varphi}^{2}(\sigma_{1}^{2}+\sigma_{2}^{2})} \end{matrix} \right)$$

The residual covariance matrix is

$$\boldsymbol{\Sigma}=E\left( \mathbf{Y}-\mathbf{X}\hat{\boldsymbol{g}} \right)\left( \mathbf{Y}\boldsymbol{-}\mathbf{X}\hat{\boldsymbol{g}} \right)^{\boldsymbol{T}}$$

$$=\boldsymbol{\Sigma}_{\boldsymbol{Y}}-E(\mathbf{X}\hat{\boldsymbol{g}}\mathbf{Y}^{\boldsymbol{T}}+\mathbf{Y}{\hat{\boldsymbol{g}}}^{\boldsymbol{T}}\mathbf{X}^{\boldsymbol{T}}-\mathbf{X}\hat{\boldsymbol{g}}{\hat{\boldsymbol{g}}}^{\boldsymbol{T}}\mathbf{X}^{\boldsymbol{T}})$$

where

$$E(\mathbf{X}\hat{\boldsymbol{g}}\mathbf{Y}^{\boldsymbol{T}})=E(\mathbf{Y}{\hat{\boldsymbol{g}}}^{\boldsymbol{T}}\mathbf{X}^{\boldsymbol{T}})=\left( \begin{matrix} \hat{b}\mathrm{Cov}\left( X_{1},Y_{1} \right)+\hat{d}\mathrm{Cov}\left( X_{m}^{'},Y_{1} \right)+\hat{f}\mathrm{Cov}(X_{f}^{'},Y_{1}) & \hat{b}\mathrm{Cov}\left( X_{1},Y_{2} \right)+\hat{d}\mathrm{Cov}\left( X_{m}^{'},Y_{2} \right)+\hat{f}\mathrm{Cov}(X_{f}^{'},Y_{2}) \\ \hat{b}\mathrm{Cov}\left( X_{2},Y_{1} \right)+\hat{d}\mathrm{Cov}\left( X_{m}^{'},Y_{1} \right)+\hat{f}\mathrm{Cov}(X_{f}^{'},Y_{1}) & \hat{b}\mathrm{Cov}\left( X_{2},Y_{2} \right)+\hat{d}\mathrm{Cov}\left( X_{m}^{'},Y_{2} \right)+\hat{f}\mathrm{Cov}(X_{f}^{'},Y_{2}) \end{matrix} \right)$$

$$E\left( \mathbf{X}\hat{\boldsymbol{g}}{\hat{\boldsymbol{g}}}^{\boldsymbol{T}}\mathbf{X}^{\boldsymbol{T}} \right)=\left( \begin{matrix} \hat{b}^{2}\mathrm{Var}\left( X_{1} \right){+\hat{d}}^{2}\mathrm{Var}\left( X_{m}^{'} \right)+\hat{f}^{2}\mathrm{Var}\left( X_{f}^{'} \right)+2\hat{b}\hat{d}\mathrm{Cov}\left( X_{m}^{'},X_{1} \right)+2\hat{b}\hat{f}\mathrm{Cov}\left( X_{f}^{'},X_{1} \right)+2\hat{d}\hat{f}\mathrm{Cov}\left( X_{f}^{'},X_{m}^{'} \right) & \hat{b}^{2}\mathrm{Cov}\left( X_{1},X_{2} \right){+\hat{d}}^{2}\mathrm{Var}\left( X_{m}^{'} \right)+\hat{f}^{2}\mathrm{Var}\left( X_{f}^{'} \right)+2\hat{b}\hat{d}\mathrm{Cov}\left( X_{m}^{'},X_{1} \right)+2\hat{b}\hat{f}\mathrm{Cov}\left( X_{f}^{'},X_{1} \right)+2\hat{d}\hat{f}\mathrm{Cov}\left( X_{f}^{'},X_{m}^{'} \right) \\ \hat{b}^{2}\mathrm{Cov}\left( X_{1},X_{2} \right){+\hat{d}}^{2}\mathrm{Var}\left( X_{m}^{'} \right)+\hat{f}^{2}\mathrm{Var}\left( X_{f}^{'} \right)+2\hat{b}\hat{d}\mathrm{Cov}\left( X_{m}^{'},X_{2} \right)+2\hat{b}\hat{f}\mathrm{Cov}\left( X_{f}^{'},X_{2} \right)+2\hat{d}\hat{f}\mathrm{Cov}\left( X_{f}^{'},X_{m}^{'} \right) & \hat{b}^{2}\mathrm{Var}\left( X_{2} \right){+\hat{d}}^{2}\mathrm{Var}\left( X_{m}^{'} \right)+\hat{f}^{2}\mathrm{Var}\left( X_{f}^{'} \right)+2\hat{b}\hat{d}\mathrm{Cov}\left( X_{m}^{'},X_{2} \right)+2\hat{b}\hat{f}\mathrm{Cov}\left( X_{f}^{'},X_{2} \right)+2\hat{d}\hat{f}\mathrm{Cov}\left( X_{f}^{'},X_{m}^{'} \right) \end{matrix} \right)$$

The expected -2lnL of the model per sibling pair is

$$E(-2lnL)=ln|\boldsymbol{\Sigma}|+2$$

**R code for simulations for exploring bias, power and type 1 error of genetic association**

**Full sibling pairs at autosomal loci**

library(lmerTest)

library(MASS)

rm(list=ls())

Nrep = 1000 #Number of simulation replicates

N = 2000 #Sample size

Vm = 0.001 #Variance explained by maternal effect

Vf = 0.001 #Variance explained by fetal effect

p = 0.1 #Increaser allele frequency

Opp = FALSE #Boolean variable to denote whether maternal and fetal effects in opposite directions (TRUE = opposite directions)

rho = 0.2 #Covariance between sibling residual variances

q <- 1-p #Decreaser allele frequency

#Create vectors to save results from regression analyses

#For analysis using simulated parental and fetal genotypes

beta_genotyped_mat <- vector(length = Nrep) #Fitting regression with maternal genotypes known (maternal coefficient)

beta_genotyped_fet <- vector(length = Nrep) #Fitting regression with maternal genotypes known (fetal coefficient)

se_genotyped_mat <- vector(length = Nrep)

se_genotyped_fet <- vector(length = Nrep)

pval_genotyped_mat <- vector(length = Nrep)

pval_genotyped_fet <- vector(length = Nrep)

#Analysis using imputed parental genotypes and simulated fetal genotypes

beta_imputed_mat <- vector(length = Nrep) #Fitting regression with genotypes imputed (maternal coefficient)

beta_imputed_fet <- vector(length = Nrep) #Fitting regression with genotypes imputed (fetal coefficient)

se_imputed_mat <- vector(length = Nrep)

se_imputed_fet <- vector(length = Nrep)

pval_imputed_mat <- vector(length = Nrep)

pval_imputed_fet <- vector(length = Nrep)

#Analysis using only sib genotypes

beta_sib <- vector(length = Nrep) #Fitting incorrect model to just sibs

se_sib <- vector(length = Nrep)

pval_sib <- vector(length = Nrep)

#ANOVA for full model against null model

pval_imputed_omni <- vector(length = Nrep)

pval_genotyped_omni <- vector(length = Nrep)

a <- sqrt(1/(2*p*q)) #Create genetic variable of variance one. Assume no dominance.

BetaM <- sqrt(Vm) #Path coefficient for maternal effect

BetaF <- sqrt(Vf) #Path coefficient for fetal effect

if(Opp == TRUE) { #If Opp is true then maternal and fetal effects have opposite directions of effect

BetaF = -BetaF

}

Ve <- (1 - BetaM^2 - BetaF^2 - 2*0.5*BetaM*BetaF) #Residual variance in trait

for(j in 1:Nrep) {

#Sample mothers' genotypes

Zm <- sample(x = c("AA","Aa","aa"), size = N, replace = TRUE, prob = c(p^2, 2*p*q, q^2))

#Sample fathers' genotypes

Zp <- sample(x = c("AA","Aa","aa"), size = N, replace = TRUE, prob = c(p^2, 2*p*q, q^2))

Z_sib1 <- vector(length = N)

Z_sib2 <- vector(length = N)

Z_imp <- vector(length = N) #Imputed vector of genotypes at mother's locus

#Simulate sib 1 genotype

r <- runif(N)

for (i in 1:N) {

Z_sib1[i] <- switch(paste(Zm[i],Zp[i]),

'AA AA'='AA',

'AA Aa'=ifelse(r[i] <= 0.5, 'AA', 'Aa'),

'AA aa'='Aa',

'Aa AA'=ifelse(r[i] <= 0.5, 'AA', 'Aa'),

'Aa Aa'=ifelse(r[i] <= 0.25, 'aa', ifelse(r[i] > 0.75, 'AA', 'Aa')),

'Aa aa'=ifelse(r[i] <= 0.5, 'aa', 'Aa'),

'aa AA'='Aa',

'aa Aa'=ifelse(r[i] <= 0.5, 'aa', 'Aa'),

'aa aa'='aa')

}

#Simulate sib 2 genotype

r <- runif(N)

for (i in 1:N) {

Z_sib2[i] <- switch(paste(Zm[i], Zp[i]),

'AA AA'='AA',

'AA Aa'=ifelse(r[i] <= 0.5, 'AA', 'Aa'),

'AA aa'='Aa',

'Aa AA'=ifelse(r[i] <= 0.5, 'AA', 'Aa'),

'Aa Aa'=ifelse(r[i] <= 0.25, 'aa', ifelse(r[i] > 0.75, 'AA', 'Aa')),

'Aa aa'=ifelse(r[i] <= 0.5, 'aa', 'Aa'),

'aa AA'='Aa',

'aa Aa'=ifelse(r[i] <= 0.5, 'aa', 'Aa'),

'aa aa'='aa')

}

#Change coding to 0, 1 and 2 in order to calculate allele frequencies

Z_sib1_012 <- ifelse(Z_sib1=='AA', 0, ifelse(Z_sib1=='Aa', 1, 2))

Z_sib2_012 <- ifelse(Z_sib2=='AA', 0, ifelse(Z_sib2=='Aa', 1, 2))

#Calculate observed allele frequency based on sibling genotypes

q_obs <- sum(Z_sib1_012+Z_sib2_012)/(2*N*2)

p_obs <- 1 - q_obs

#Impute mother's genotypes

#Calculate conditional probability of mother's genotypes given sibs genotypes

p_AA_AA_AA = p_obs*(2*p_obs+2)/(p_obs+1)^2 #P(Mum = AA | Sib = AA and Sib = AA)

p_Aa_AA_AA = 2/(p_obs+1) - 1 #P(Mum = Aa | Sib = AA and Sib = AA)

p_aa_AA_AA = 0 #P(Mum = aa | Sib = AA and Sib = AA)

p_AA_AA_Aa = p_obs/(p_obs+1) #P(Mum = AA | Sib = AA and Sib = Aa)

p_Aa_AA_Aa = 1/(p_obs+1) #P(Mum = Aa | Sib = AA and Sib = Aa)

p_aa_AA_Aa = 0 #P(Mum = aa | Sib = AA and Sib = Aa)

p_AA_AA_aa = 0 #P(Mum = AA | Sib = AA and Sib = aa)

p_Aa_AA_aa = 1 #P(Mum = Aa | Sib = AA and Sib = aa)

p_aa_AA_aa = 0 #P(Mum = aa | Sib = AA and Sib = aa)

p_AA_Aa_Aa = (0.5*(p_obs-2)*p_obs)/(p_obs^2-p_obs-1) #P(Mum = AA | Sib = Aa and Sib = Aa)

p_Aa_Aa_Aa = -0.5 / ((p_obs-1)*p_obs-1) #P(Mum = Aa | Sib = Aa and Sib = Aa)

p_aa_Aa_Aa = (0.5*(p_obs-1)*(p_obs+1))/(p_obs^2-p_obs-1) #P(Mum = aa | Sib = Aa and Sib = Aa)

p_AA_aa_Aa = 0 #P(Mum = AA | Sib = aa and Sib = Aa)

p_Aa_aa_Aa = 1/(q_obs+1) #P(Mum = Aa | Sib = aa and Sib = Aa)

p_aa_aa_Aa = q_obs/(q_obs+1) #P(Mum = aa | Sib = aa and Sib = Aa)

p_AA_aa_aa = 0 #P(Mum = AA | Sib = aa and Sib = aa)

p_Aa_aa_aa = 2/(q_obs+1) - 1 #P(Mum = Aa | Sib = aa and Sib = aa)

p_aa_aa_aa = q_obs*(2*q_obs+2)/(q_obs+1)^2 #P(Mum = aa | Sib = aa and Sib = aa)

for (i in 1:N) {

Z_imp[i] <- switch(paste(Z_sib1[i], Z_sib2[i]),

'AA AA'= -a*p_AA_AA_AA + 0*p_Aa_AA_AA + a*p_aa_AA_AA,

'AA Aa'= -a*p_AA_AA_Aa + 0*p_Aa_AA_Aa + a*p_aa_AA_Aa,

'AA aa'= -a*p_AA_AA_aa + 0*p_Aa_AA_aa + a*p_aa_AA_aa,

'Aa AA'= -a*p_AA_AA_Aa + 0*p_Aa_AA_Aa + a*p_aa_AA_Aa,

'Aa Aa'= -a*p_AA_Aa_Aa + 0*p_Aa_Aa_Aa + a*p_aa_Aa_Aa,

'Aa aa'= -a*p_AA_aa_Aa + 0*p_Aa_aa_Aa + a*p_aa_aa_Aa,

'aa AA'= -a*p_AA_AA_aa + 0*p_Aa_AA_aa + a*p_aa_AA_aa,

'aa Aa'= -a*p_AA_aa_Aa + 0*p_Aa_aa_Aa + a*p_aa_aa_Aa,

'aa aa'= -a*p_AA_aa_aa + 0*p_Aa_aa_aa + a*p_aa_aa_aa)

}

#Change coding AA/Aa/aa to the genetic value -a/0/a

Z_sib1 <- ifelse(Z_sib1=='AA', -a, ifelse(Z_sib1=='Aa', 0, a))

Z_sib2 <- ifelse(Z_sib2=='AA', -a, ifelse(Z_sib2=='Aa', 0, a))

Zm <- ifelse(Zm=='AA', -a, ifelse(Zm=='Aa', 0, a))

Zp <- ifelse(Zp=='AA', -a, ifelse(Zp=='Aa', 0, a))

#Create correlated error variables for sib 1 and sib 2

Sigma <- matrix(c(Ve , rho, rho, Ve),2,2)

e <- mvrnorm(n = N, mu = c(0, 0), Sigma)

#Simulate offspring outcome

Y_sib1 <- BetaM*Zm + BetaF*Z_sib1 + e[,1]

Y_sib2 <- BetaM*Zm + BetaF*Z_sib2 + e[,2]

#Create a test dataset of simulated and imputed genotypes and phenotype (offspring outcome)

test <- data.frame(rbind(cbind(1:N, Y_sib1, Zm, Z_imp, Z_sib1),

cbind(1:N, Y_sib2, Zm, Z_imp, Z_sib2)))

colnames(test) <- c("fam", "Y","Zm", "Z_imp", "Z")

#Run analyses

lmer_genotyped <- lmer(Y ~ Zm + Z + (1|fam), data = test, REML = FALSE); results_genotyped <- summary(lmer_genotyped)

lmer_imputed <- lmer(Y ~ Z_imp + Z + (1|fam), data = test, REML = FALSE); results_imputed <- summary(lmer_imputed)

lmer_sib <- lmer(Y ~ Z + (1|fam), data = test, REML = FALSE); results_sib <- summary(lmer_sib)

lmer_null <- lmer(Y ~ (1|fam), data = test, REML = FALSE)

beta_genotyped_mat[j] <- results_genotyped$coefficient[2,1]

se_genotyped_mat[j] <- results_genotyped$coefficient[2,2]

pval_genotyped_mat[j] <- results_genotyped$coefficient[2,5]

beta_genotyped_fet[j] <- results_genotyped$coefficient[3,1]

se_genotyped_fet[j] <- results_genotyped$coefficient[3,2]

pval_genotyped_fet[j] <- results_genotyped$coefficient[3,5]

pval_genotyped_omni[j] <- anova(lmer_genotyped,lmer_null)[2,8]

beta_imputed_mat[j] <- results_imputed$coefficient[2,1]

se_imputed_mat[j] <- results_imputed$coefficient[2,2]

pval_imputed_mat[j] <- results_imputed$coefficient[2,5]

beta_imputed_fet[j] <- results_imputed$coefficient[3,1]

se_imputed_fet[j] <- results_imputed$coefficient[3,2]

pval_imputed_fet[j] <- results_imputed$coefficient[3,5]

pval_imputed_omni[j] <- anova(lmer_imputed,lmer_null)[2,8]

beta_sib[j] <- results_sib$coefficient[2,1]

se_sib[j] <- results_sib$coefficient[2,2]

pval_sib[j] <- results_sib$coefficient[2,5]

}

**Half sibling pairs at autosomal loci**

library(lmerTest)

library(MASS)

rm(list=(ls()))

Nrep = 1000 #Number of simulation replicates

N = 2000 #Sample size

Vm = 0.001 #Variance explained by maternal effect

Vp = 0.001 #Variance explained by paternal effect

Vf = 0.001 #Variance explained by fetal effect

p = 0.1 #Increaser allele frequency

Opp = FALSE #Boolean variable to denote whether maternal and fetal effects in opposite directions (TRUE = opposite directions)

rho = 0.2 #Covariance between half sibling residual variances

q <- 1-p #Decreaser allele frequency

#Create vectors to save results from regression analyses

#For analysis using simulated genotypes

beta_genotyped_mat <- vector(length = Nrep) #Fitting regression with maternal and paternal genotypes known (maternal coefficient)

beta_genotyped_pat <- vector(length = Nrep) #Fitting regression with maternal and paternal genotypes known (paternal coefficient)

beta_genotyped_fet <- vector(length = Nrep) #Fitting regression with maternal and paternal genotypes known (fetal coefficient)

se_genotyped_mat <- vector(length = Nrep)

se_genotyped_pat <- vector(length = Nrep)

se_genotyped_fet <- vector(length = Nrep)

pval_genotyped_mat <- vector(length = Nrep)

pval_genotyped_pat <- vector(length = Nrep)

pval_genotyped_fet <- vector(length = Nrep)

#Analysis using imputed maternal and paternal genotypes

beta_imputed_mat <- vector(length = Nrep) #Fitting regression with genotypes imputed (maternal coefficient)

beta_imputed_pat <- vector(length = Nrep) #Fitting regression with genotypes imputed (paternal coefficient)

beta_imputed_fet <- vector(length = Nrep) #Fitting regression with genotypes imputed (fetal coefficient)

se_imputed_mat <- vector(length = Nrep)

se_imputed_pat <- vector(length = Nrep)

se_imputed_fet <- vector(length = Nrep)

pval_imputed_mat <- vector(length = Nrep)

pval_imputed_pat <- vector(length = Nrep)

pval_imputed_fet <- vector(length = Nrep)

#Analysis using only half sibling genotypes

beta_fet <- vector(length = Nrep) #Fitting incorrect model including only half siblings

se_fet <- vector(length = Nrep)

pval_fet <- vector(length = Nrep)

#ANOVA for full model against null model

pval_genotyped_omni <- vector(length = Nrep)

pval_imputed_omni <- vector(length = Nrep)

a <- sqrt(1/2*p*q) #Create genetic variable of one. Assume no dominance.

BetaM <- sqrt(Vm) #Path coefficient for maternal effect

BetaP <- sqrt(Vp) #Path coefficient for paternal effect

BetaF <- sqrt(Vf) #Path coefficient for fetal effect

if(Opp == TRUE) { #If Opp is true then maternal and fetal effects have opposite directions of effect

BetaF = -BetaF

}

Ve <- (1 - BetaM^2 - BetaP^2 - BetaF^2 - 2*0.5*BetaM*BetaF - 2*0.5*BetaP*BetaF) #Residual variance in trait for half siblings

for(j in 1:Nrep) {

#Sample mothers' genotypes

Zm <- sample(x = c('AA','Aa','aa'), size = N, replace = TRUE, prob = c(p^2, 2*p*q, q^2))

#Sample fathers' genotypes

Zp1 <- sample(x = c('AA','Aa','aa'), size = N, replace = TRUE, prob = c(p^2, 2*p*q, q^2))

Zp2 <- sample(x = c('AA','Aa','aa'), size = N, replace = TRUE, prob = c(p^2, 2*p*q, q^2))

Z_hsib1 <- vector(length = N)

Z_hsib2 <- vector(length = N)

Zm_imp <- vector(length = N) #Imputed vector of genotypes at mum's locus

Zp1_imp <- vector(length = N) #Imputed vector of genotypes at dad1's locus

Zp2_imp <- vector(length = N) #Imputed vector of genotypes at dad2's locus

#Simulate half sibling 1 genotype

r <- runif(N)

for (i in 1:N) {

Z_hsib1[i] <- switch(paste(Zm[i],Zp1[i]),

'AA AA'='AA',

'AA Aa'=ifelse(r[i] <= 0.5, 'AA', 'Aa'),

'AA aa'='Aa',

'Aa AA'=ifelse(r[i] <= 0.5, 'AA', 'Aa'),

'Aa Aa'=ifelse(r[i] <= 0.25, 'aa', ifelse(r[i] > 0.75, 'AA', 'Aa')),

'Aa aa'=ifelse(r[i] <= 0.5, 'aa', 'Aa'),

'aa AA'='Aa',

'aa Aa'=ifelse(r[i] <= 0.5, 'aa', 'Aa'),

'aa aa'='aa')

}

#Simulate half sibling 2 genotype

r <- runif(N)

for (i in 1:N) {

Z_hsib2[i] <- switch(paste(Zm[i],Zp2[i]),

'AA AA'='AA',

'AA Aa'=ifelse(r[i] <= 0.5, 'AA', 'Aa'),

'AA aa'='Aa',

'Aa AA'=ifelse(r[i] <= 0.5, 'AA', 'Aa'),

'Aa Aa'=ifelse(r[i] <= 0.25, 'aa', ifelse(r[i] > 0.75, 'AA', 'Aa')),

'Aa aa'=ifelse(r[i] <= 0.5, 'aa', 'Aa'),

'aa AA'='Aa',

'aa Aa'=ifelse(r[i] <= 0.5, 'aa', 'Aa'),

'aa aa'='aa')

}

#Change coding to 0, 1 and 2 in order to calculate allele frequencies

Z_hsib1_012 <- ifelse(Z_hsib1=='AA', 0, ifelse(Z_hsib1=='Aa', 1, 2))

Z_hsib2_012 <- ifelse(Z_hsib2=='AA', 0, ifelse(Z_hsib2=='Aa', 1, 2))

#Calculate observed allele frequency based on half sibling genotypes

q_obs <- sum(Z_hsib1_012, Z_hsib2_012)/(2*N*2)

p_obs <- 1 - q_obs

#Impute mother's genotypes

#Calculate conditional probability of mother's genotypes given half sibs genotypes

p_AA_AA_AA = (2*p_obs)/(p_obs+1) #P(Mum = AA | HSib = AA and HSib = AA)

p_Aa_AA_AA = (1-p_obs)/(p_obs+1) #P(Mum = Aa | HSib = AA and HSib = AA)

p_aa_AA_AA = 0 #P(Mum = aa | HSib = AA and HSib = AA)

p_AA_AA_Aa = (2*p_obs)/(2*p_obs+1) #P(Mum = AA | HSib = AA and HSib = Aa)

p_Aa_AA_Aa = 1/(2*p_obs+1) #P(Mum = Aa | HSib = AA and HSib = Aa)

p_aa_AA_Aa = 0 #P(Mum = aa | HSib = AA and HSib = Aa)

p_AA_AA_aa = 0 #P(Mum = AA | HSib = AA and HSib = aa)

p_Aa_AA_aa = 1 #P(Mum = Aa | HSib = AA and HSib = aa)

p_aa_AA_aa = 0 #P(Mum = aa | HSib = AA and HSib = aa)

p_AA_Aa_Aa = ((2*p_obs)*(1-p_obs))/((4*p_obs)*(1-p_obs)+1) #P(Mum = AA | HSib = Aa and HSib = Aa)

p_Aa_Aa_Aa = (1-p_obs)/((p_obs-1)*(4*p_obs^2-4*p_obs-1)) #P(Mum = Aa | HSib = Aa and HSib = Aa)

p_aa_Aa_Aa = ((2*q_obs)*(1-q_obs))/((4*q_obs)*(1-q_obs)+1) #P(Mum = aa | HSib = Aa and HSib = Aa)

p_AA_aa_Aa = 0 #P(Mum = AA | HSib = aa and HSib = Aa)

p_Aa_aa_Aa = 1/(2*q_obs+1) #P(Mum = Aa | HSib = aa and HSib = Aa)

p_aa_aa_Aa = (2*q_obs)/(2*q_obs+1) #P(Mum = aa | HSib = aa and HSib = Aa)

p_AA_aa_aa = 0 #P(Mum = AA | HSib = aa and HSib = aa)

p_Aa_aa_aa = (1-q_obs)/(q_obs+1) #P(Mum = Aa | HSib = aa and HSib = aa)

p_aa_aa_aa = (2*q_obs)/(q_obs+1) #P(Mum = aa | HSib = aa and HSib = aa)

for (i in 1:N) {

Zm_imp[i] <- switch(paste(Z_hsib1[i],Z_hsib2[i]),

'AA AA'= -a*p_AA_AA_AA + 0*p_Aa_AA_AA + a*p_aa_AA_AA,

'AA Aa'= -a*p_AA_AA_Aa + 0*p_Aa_AA_Aa + a*p_aa_AA_Aa,

'AA aa'= -a*p_AA_AA_aa + 0*p_Aa_AA_aa + a*p_aa_AA_aa,

'Aa AA'= -a*p_AA_AA_Aa + 0*p_Aa_AA_Aa + a*p_aa_AA_Aa,

'Aa Aa'= -a*p_AA_Aa_Aa + 0*p_Aa_Aa_Aa + a*p_aa_Aa_Aa,

'Aa aa'= -a*p_AA_aa_Aa + 0*p_Aa_aa_Aa + a*p_aa_aa_Aa,

'aa AA'= -a*p_AA_AA_aa + 0*p_Aa_AA_aa + a*p_aa_AA_aa,

'aa Aa'= -a*p_AA_aa_Aa + 0*p_Aa_aa_Aa + a*p_aa_aa_Aa,

'aa aa'= -a*p_AA_aa_aa + 0*p_Aa_aa_aa + a*p_aa_aa_aa)

}

#Impute father's genotypes

#Calculate conditional probability of father's genotypes given half sibling genotype

p_dAA_AA_AA <- p_obs #P(Dad = AA | HSib = AA and HSib = AA)

p_dAa_AA_AA <- q_obs #P(Dad = Aa | HSib = AA and HSib = AA)

p_daa_AA_AA <- 0 #P(Dad = aa | HSib = AA and HSib = AA)

p_dAA_AA_Aa <- p_obs #P(Dad = AA | HSib = AA and HSib = Aa)

p_dAa_AA_Aa <- q_obs #P(Dad = Aa | HSib = AA and HSib = Aa)

p_daa_AA_Aa <- 0 #P(Dad = aa | HSib = AA and HSib = Aa)

p_dAA_AA_aa <- p_obs #P(Dad = AA | HSib = AA and HSib = aa)

p_dAa_AA_aa <- q_obs #P(Dad = Aa | HSib = AA and HSib = aa)

p_daa_AA_aa <- 0 #P(Dad = aa | HSib = AA and HSib = aa)

p_dAA_Aa_AA <- p_obs^2/(2*p_obs+1) #P(Dad = AA | HSib = Aa and HSib = AA)

p_dAa_Aa_AA <- 2*p_obs/(2*p_obs+1) #P(Dad = Aa | HSib = Aa and HSib = AA)

p_daa_Aa_AA <- (1-p_obs^2)/(2*p_obs+1) #P(Dad = aa | HSib = Aa and HSib = AA)

p_dAA_dAA_Aa_Aa <- (2*p_obs^3-p_obs^4)/(1+4*p_obs-4*p_obs^2) #P(Dad1 = AA and Dad2 = AA | HSib1 = Aa and HSib2 = Aa)

p_dAA_dAa_Aa_Aa <- (2*p_obs^2-2*p_obs^3)/(1+4*p_obs-4*p_obs^2) #P(Dad1 = AA and Dad2 = Aa | HSib1 = Aa and HSib2 = Aa)

p_dAA_daa_Aa_Aa <- (p_obs^2*q_obs^2)/(1+4*p_obs-4*p_obs^2) #P(Dad1 = AA and Dad2 = aa | HSib1 = Aa and HSib2 = Aa)

p_dAa_dAA_Aa_Aa <- p_dAA_dAa_Aa_Aa #P(Dad1 = Aa and Dad2 = AA | HSib1 = Aa and HSib2 = Aa)

p_dAa_dAa_Aa_Aa <- (2*p_obs*q_obs)/(1+4*p_obs-4*p_obs^2) #P(Dad1 = Aa and Dad2 = Aa | HSib1 = Aa and HSib2 = Aa)

p_dAa_daa_Aa_Aa <- (2*q_obs^2-2*q_obs^3)/(1+4*q_obs-4*q_obs^2) #P(Dad1 = Aa and Dad2 = aa | HSib1 = Aa and HSib2 = Aa)

p_daa_dAA_Aa_Aa <- p_dAA_daa_Aa_Aa #P(Dad1 = aa and Dad2 = AA | HSib1 = Aa and HSib2 = Aa)

p_daa_dAa_Aa_Aa <- p_dAa_daa_Aa_Aa #P(Dad1 = aa and Dad2 = Aa | HSib1 = Aa and HSib2 = Aa)

p_daa_daa_Aa_Aa <- (2*q_obs^3-q_obs^4)/(1+4*q_obs-4*q_obs^2) #P(Dad1 = aa and Dad2 = aa | HSib1 = Aa and HSib2 = Aa)

p_dAA_Aa_aa <- p_obs*(2-p_obs)/(3-2*p_obs) #P(Dad = AA | HSib = Aa and HSib = aa)

p_dAa_Aa_aa <- (2-2*p_obs)/(3-2*p_obs) #P(Dad = Aa | HSib = Aa and HSib = aa)

p_daa_Aa_aa <- (1-p_obs)^2/(3-2*p_obs) #P(Dad = aa | HSib = Aa and HSib = aa)

p_dAA_aa_AA <- 0 #P(Dad = AA | HSib = aa and HSib = AA)

p_dAa_aa_AA <- p_obs #P(Dad = Aa | HSib = aa and HSib = AA)

p_daa_aa_AA <- q_obs #P(Dad = aa | HSib = aa and HSib = AA)

p_dAA_aa_Aa <- 0 #P(Dad = AA | HSib = aa and HSib = Aa)

p_dAa_aa_Aa <- p_obs #P(Dad = Aa | HSib = aa and HSib = Aa)

p_daa_aa_Aa <- q_obs #P(Dad = aa | HSib = aa and HSib = Aa)

p_dAA_aa_aa <- 0 #P(Dad = AA | HSib = aa and HSib = aa)

p_dAa_aa_aa <- p_obs #P(Dad = Aa | HSib = aa and HSib = aa)

p_daa_aa_aa <- q_obs #P(Dad = aa | HSib = aa and HSib = aa)

for (i in 1:N) {

Zp1_imp[i] <- switch(paste(Z_hsib1[i],Z_hsib2[i]),

'AA AA'= -a*p_dAA_AA_AA + 0*p_dAa_AA_AA + a*p_daa_AA_AA,

'AA Aa'= -a*p_dAA_AA_Aa + 0*p_dAa_AA_Aa + a*p_daa_AA_Aa,

'AA aa'= -a*p_dAA_AA_aa + 0*p_dAa_AA_aa + a*p_daa_AA_aa,

'Aa AA'= -a*p_dAA_Aa_AA + 0*p_dAa_Aa_AA + a*p_daa_Aa_AA,

'Aa Aa'= -a*(p_dAA_dAA_Aa_Aa + p_dAA_dAa_Aa_Aa + p_dAA_daa_Aa_Aa) +

0*(p_dAa_dAA_Aa_Aa + p_dAa_dAa_Aa_Aa + p_dAa_daa_Aa_Aa) +

a*(p_daa_dAA_Aa_Aa + p_daa_dAa_Aa_Aa + p_daa_daa_Aa_Aa),

'Aa aa'= -a*p_dAA_Aa_aa + 0*p_dAa_Aa_aa + a*p_daa_Aa_aa,

'aa AA'= -a*p_dAA_aa_AA + 0*p_dAa_aa_AA + a*p_daa_aa_AA,

'aa Aa'= -a*p_dAA_aa_Aa + 0*p_dAa_aa_Aa + a*p_daa_aa_Aa,

'aa aa'= -a*p_dAA_aa_aa + 0*p_dAa_aa_aa + a*p_daa_aa_aa)

Zp2_imp[i] <- switch(paste(Z_hsib2[i],Z_hsib1[i]),

'AA AA'= -a*p_dAA_AA_AA + 0*p_dAa_AA_AA + a*p_daa_AA_AA,

'AA Aa'= -a*p_dAA_AA_Aa + 0*p_dAa_AA_Aa + a*p_daa_AA_Aa,

'AA aa'= -a*p_dAA_AA_aa + 0*p_dAa_AA_aa + a*p_daa_AA_aa,

'Aa AA'= -a*p_dAA_Aa_AA + 0*p_dAa_Aa_AA + a*p_daa_Aa_AA,

'Aa Aa'= -a*(p_dAA_dAA_Aa_Aa + p_dAA_dAa_Aa_Aa + p_dAA_daa_Aa_Aa) +

0*(p_dAa_dAA_Aa_Aa + p_dAa_dAa_Aa_Aa + p_dAa_daa_Aa_Aa) +

a*(p_daa_dAA_Aa_Aa + p_daa_dAa_Aa_Aa + p_daa_daa_Aa_Aa),

'Aa aa'= -a*p_dAA_Aa_aa + 0*p_dAa_Aa_aa + a*p_daa_Aa_aa,

'aa AA'= -a*p_dAA_aa_AA + 0*p_dAa_aa_AA + a*p_daa_aa_AA,

'aa Aa'= -a*p_dAA_aa_Aa + 0*p_dAa_aa_Aa + a*p_daa_aa_Aa,

'aa aa'= -a*p_dAA_aa_aa + 0*p_dAa_aa_aa + a*p_daa_aa_aa)

}

#Convert AA/Aa/aa to genetic value -a/0/a

Zm <- ifelse(Zm=='AA', -a, ifelse(Zm=='Aa', 0, a))

Zp1 <- ifelse(Zp1=='AA', -a, ifelse(Zp1=='Aa', 0, a))

Zp2 <- ifelse(Zp2=='AA', -a, ifelse(Zp2=='Aa', 0, a))

Z_hsib1 <- ifelse(Z_hsib1=='AA', -a, ifelse(Z_hsib1=='Aa', 0, a))

Z_hsib2 <- ifelse(Z_hsib2=='AA', -a, ifelse(Z_hsib2=='Aa', 0, a))

#Create correlated error variables for sib 1 and sib 2 in the model with both parents

Sigma <- matrix(c(Ve, rho, rho, Ve),2,2)

e <- mvrnorm(n = N, mu = c(0, 0), Sigma)

#Simulate offspring outcome

Y_hsib1 <- BetaM*Zm + BetaP*Zp1 + BetaF*Z_hsib1 + e[,1]

Y_hsib2 <- BetaM*Zm + BetaP*Zp2 + BetaF*Z_hsib2 + e[,2]

test <- data.frame(rbind(cbind(1:N, Y_hsib1, Zm, Zp1, Zm_imp, Zp1_imp, Z_hsib1),

cbind(1:N, Y_hsib2, Zm, Zp2, Zm_imp, Zp2_imp, Z_hsib2)))

colnames(test) <- c("fam", "Y","Zm", "Zp", "Zm_imp", "Zp_imp", "Z")

#Run analyses

lmer_genotyped <- lmer(Y ~ Zm + Zp + Z + (1|fam), data = test, REML = FALSE); results_genotyped <- summary(lmer_genotyped)

lmer_imputed <- lmer(Y ~ Zm_imp + Zp_imp + Z + (1|fam), data = test, REML = FALSE); results_imputed <- summary(lmer_imputed)

lmer_hsib <- lmer(Y ~ Z + (1|fam), data = test, REML = FALSE); results_hsib <- summary(lmer_hsib)

lmer_null <- lmer(Y ~ (1|fam), data = test, REML = FALSE)

beta_genotyped_mat[j] <- results_genotyped$coefficient[2,1]

se_genotyped_mat[j] <- results_genotyped$coefficient[2,2]

pval_genotyped_mat[j] <- results_genotyped$coefficient[2,5]

beta_genotyped_pat[j] <- results_genotyped$coefficient[3,1]

se_genotyped_pat[j] <- results_genotyped$coefficient[3,2]

pval_genotyped_pat[j] <- results_genotyped$coefficient[3,5]

beta_genotyped_fet[j] <- results_genotyped$coefficient[4,1]

se_genotyped_fet[j] <- results_genotyped$coefficient[4,2]

pval_genotyped_fet[j] <- results_genotyped$coefficient[4,5]

pval_genotyped_omni[j] <- anova(lmer_genotyped,lmer_null)[2,8]

beta_imputed_mat[j] <- results_imputed$coefficient[2,1]

se_imputed_mat[j] <- results_imputed$coefficient[2,2]

pval_imputed_mat[j] <- results_imputed$coefficient[2,5]

beta_imputed_pat[j] <- results_imputed$coefficient[3,1]

se_imputed_pat[j] <- results_imputed$coefficient[3,2]

pval_imputed_pat[j] <- results_imputed$coefficient[3,5]

beta_imputed_fet[j] <- results_imputed$coefficient[4,1]

se_imputed_fet[j] <- results_imputed$coefficient[4,2]

pval_imputed_fet[j] <- results_imputed$coefficient[4,5]

pval_imputed_omni[j] <- anova(lmer_imputed,lmer_null)[2,8]

beta_fet[j] <- results_hsib$coefficient[2,1]

se_fet[j] <- results_hsib$coefficient[2,2]

pval_fet[j] <- results_hsib$coefficient[2,5]

}

**Male sibling pairs at X chromosomal loci**

library(lmerTest)

library(MASS)

rm(list=ls())

Nrep = 1000 #Number of simulation replicates

N = 2000 #Sample size

Vm = 0.001 #Variance explained by maternal effect

Vp = 0.00 #Variance explained by paternal effect

Vf = 0.001 #Variance explained by fetal effect

p = 0.1 #Increaser allele frequency

Opp = FALSE #Boolean variable to denote whether maternal and fetal effects in opposite directions (TRUE = opposite directions)

rho = 0.2 #Covariance between sibling residual variances

q <- 1-p #Decreaser allele frequency

#Create vectors to save results from regression analyses

#For analysis using simulated genotypes:

beta_genotyped_mat <- vector(length = Nrep) #Fitting regression with maternal and paternal genotypes known (maternal coefficient)

beta_genotyped_fet <- vector(length = Nrep) #Fitting regression with maternal and paternal genotypes known (fetal coefficient)

se_genotyped_mat <- vector(length = Nrep)

se_genotyped_fet <- vector(length = Nrep)

pval_genotyped_mat <- vector(length = Nrep)

pval_genotyped_fet <- vector(length = Nrep)

#Analysis using imputed maternal and paternal genotypes:

beta_imputed_mat <- vector(length = Nrep) #Fitting regression with genotypes imputed (maternal coefficient)

beta_imputed_fet <- vector(length = Nrep) #Fitting regression with genotypes imputed (fetal coefficient)

se_imputed_mat <- vector(length = Nrep)

se_imputed_fet <- vector(length = Nrep)

pval_imputed_mat <- vector(length = Nrep)

pval_imputed_fet <- vector(length = Nrep)

#Analysis using only sibling genotypes:

beta_sib <- vector(length = Nrep) #Fitting incorrect model including only siblings

se_sib <- vector(length = Nrep)

pval_sib <- vector(length = Nrep)

#ANOVA for full model against null model

pval_genotyped_omni <- vector(length = Nrep)

pval_imputed_omni <- vector(length = Nrep)

a <- sqrt(1/(2*p*q)) #Create genetic variable of variance one for maternal SNPs on X chromosome. Assume no dominance.

BetaM <- sqrt(Vm) #Path coefficient for maternal effect

BetaP <- sqrt(Vp/2) #Path coefficient for paternal effect

BetaF <- sqrt(Vf/2) #Path coefficient for fetal effect

if(Opp == TRUE) { #If Opp is true then maternal and fetal effects have opposite directions of effect

BetaF = -BetaF

}

Ve_y <- (1 - BetaM^2 - BetaF^2*2 - BetaP^2*2 - 2*1*BetaM*BetaF) #Residual variance in trait for male sib

for(j in 1:Nrep) {

#Sample mothers' genotypes

Zm <- sample(x = c('AA','Aa','aa'), size = N, replace = TRUE, prob = c(p^2, 2*p*q, q^2))

#Sample fathers' genotypes

Zp <- sample(x = c('A','a'), size = N, replace = TRUE, prob = c(p, q))

Z_sib1y <- vector(length = N)

Z_sib2y <- vector(length = N)

Zmyy_imp <- vector(length = N) #Imputed vector of genotypes at mum's locus

#Simulate male sib1 genotype

r <- runif(N)

for (i in 1:N) {

Z_sib1y[i] <- switch(Zm[i],

'AA'='A',

'Aa'=ifelse(r[i] <= 0.5, 'A', 'a'),

'aa'='a')

}

#Simulate male sib2 genotype

r <- runif(N)

for (i in 1:N) {

Z_sib2y[i] <- switch(Zm[i],

'AA'='A',

'Aa'=ifelse(r[i] <= 0.5, 'A', 'a'),

'aa'='a')

}

#Change coding to 0, 1 and 2 in order to calculate allele frequencies

Z_sib1y_01 <- ifelse(Z_sib1y=='A', 0, 2)

Z_sib2y_01 <- ifelse(Z_sib2y=='A', 0, 2)

#Calculate observed allele frequency based on sibling one's genotypes

q_obs <- sum(c(Z_sib1y_01, Z_sib2y_01))/(2*N*2)

p_obs <- 1 - q_obs

#Impute parental genotypes from male-female sibs

#Calculate conditional probability of mother's genotypes given sibs genotypes

p_AA_A_A = (2*p_obs)/(p_obs+1) #P(Mum = AA | Sib1y = A and Sib2y = A)

p_Aa_A_A = (1-p_obs)/(p_obs+1) #P(Mum = Aa | Sib1y = A and Sib2y = A)

p_aa_A_A = 0 #P(Mum = aa | Sib1y = A and Sib2y = A)

p_AA_A_a = 0 #P(Mum = AA | Sib1y = A and Sib2y = a)

p_Aa_A_a = 1 #P(Mum = Aa | Sib1y = A and Sib2y = a)

p_aa_A_a = 0 #P(Mum = aa | Sib1y = A and Sib2y = a)

p_AA_a_A = p_AA_A_a

p_Aa_a_A = p_Aa_A_a

p_aa_a_A = p_aa_A_a

p_AA_a_a = 0 #P(Mum = AA | Sib1y = a and Sib2y = a)

p_Aa_a_a = (1-q_obs)/(q_obs+1) #P(Mum = Aa | Sib1y = a and Sib2y = a)

p_aa_a_a = (2*q_obs)/(q_obs+1) #P(Mum = aa | Sib1y = a and Sib2y = a)

for (i in 1:N) {

Zmyy_imp[i] <- switch(paste(Z_sib1y[i],Z_sib2y[i]),

'A A'= -a*p_AA_A_A + 0*p_Aa_A_A + a*p_aa_A_A,

'A a'= -a*p_AA_A_a + 0*p_Aa_A_a + a*p_aa_A_a,

'a A'= -a*p_AA_a_A + 0*p_Aa_a_A + a*p_aa_a_A,

'a a'= -a*p_AA_a_a + 0*p_Aa_a_a + a*p_aa_a_a)

}

#Convert AA/Aa/aa to genetic value

Zm <- ifelse(Zm=='AA', -a, ifelse(Zm=='Aa', 0, a))

Zp <- ifelse(Zp=='A', -a, a)

Z_sib1y <- ifelse(Z_sib1y=='A', -a, a)

Z_sib2y <- ifelse(Z_sib2y=='A', -a, a)

#Create correlated error variables for sib 1 and sib 2 in the model

Sigma_yy <- matrix(c(Ve_y, rho, rho, Ve_y),2,2) #Male-male sibs

e_yy <- mvrnorm(n = N, mu = c(0, 0), Sigma_yy)

#Simulate offspring outcome

Y_sib1y_yy <- BetaM*Zm + BetaP*Zp + BetaF*Z_sib1y + e_yy[,1]

Y_sib2y_yy <- BetaM*Zm + BetaP*Zp + BetaF*Z_sib2y + e_yy[,2]

test <- data.frame(rbind(cbind(1:N, Y_sib1y_yy, Zm, Zp, Zmyy_imp, Z_sib1y),

cbind(1:N, Y_sib2y_yy, Zm, Zp, Zmyy_imp, Z_sib2y)))

colnames(test) <- c("fam", "Y", "Zm", "Zp", "Zmyy_imp", "Zy")

#Run analyses

lmer_genotyped <- lmer(Y ~ Zm + Zy + (1|fam), data = test, REML = FALSE); results_genotyped <- summary(lmer_genotyped)

lmer_imputed <- lmer(Y ~ Zmyy_imp + Zy + (1|fam), data = test, REML = FALSE); results_imputed <- summary(lmer_imputed)

lmer_sib <- lmer(Y ~ Zy + (1|fam), data = test, REML = FALSE); results_sib <- summary(lmer_sib)

lmer_null <- lmer(Y ~ (1|fam), data = test, REML = FALSE)

beta_genotyped_mat[j] <- results_genotyped$coefficient[2,1]

se_genotyped_mat[j] <- results_genotyped$coefficient[2,2]

pval_genotyped_mat[j] <- results_genotyped$coefficient[2,5]

beta_genotyped_fet[j] <- results_genotyped$coefficient[3,1]

se_genotyped_fet[j] <- results_genotyped$coefficient[3,2]

pval_genotyped_fet[j] <- results_genotyped$coefficient[3,5]

pval_genotyped_omni[j] <- anova(lmer_genotyped,lmer_null)[2,8]

beta_imputed_mat[j] <- results_imputed$coefficient[2,1]

se_imputed_mat[j] <- results_imputed$coefficient[2,2]

pval_imputed_mat[j] <- results_imputed$coefficient[2,5]

beta_imputed_fet[j] <- results_imputed$coefficient[3,1]

se_imputed_fet[j] <- results_imputed$coefficient[3,2]

pval_imputed_fet[j] <- results_imputed$coefficient[3,5]

pval_imputed_omni[j] <- anova(lmer_imputed,lmer_null)[2,8]

beta_sib[j] <- results_sib$coefficient[2,1]

se_sib[j] <- results_sib$coefficient[2,2]

pval_sib[j] <- results_sib$coefficient[2,5]

}

**Female sibling pairs at X chromosomal loci**

library(lmerTest)

library(MASS)

rm(list=ls())

Nrep = 1000 #Number of simulation replicates

N = 2000 #Sample size

Vm = 0.001 #Variance explained by maternal effect

Vp = 0.001 #Variance explained by paternal effect

Vf = 0.001 #Variance explained by fetal effect

p = 0.1 #Increaser allele frequency

Opp = FALSE #Boolean variable to denote whether maternal and fetal effects in opposite directions (TRUE = opposite directions)

rho = 0.2 #Covariance between sibling residual variances

q <- 1-p #Decreaser allele frequency

#Create vectors to save results from regression analyses

#For analysis using simulated genotypes

beta_genotyped_mat <- vector(length = Nrep) #Fitting regression with maternal and paternal genotypes known (maternal coefficient)

beta_genotyped_pat <- vector(length = Nrep) #Fitting regression with maternal and paternal genotypes known (paternal coefficient)

beta_genotyped_fet <- vector(length = Nrep) #Fitting regression with maternal and paternal genotypes known (fetal coefficient)

se_genotyped_mat <- vector(length = Nrep)

se_genotyped_pat <- vector(length = Nrep)

se_genotyped_fet <- vector(length = Nrep)

pval_genotyped_mat <- vector(length = Nrep)

pval_genotyped_pat <- vector(length = Nrep)

pval_genotyped_fet <- vector(length = Nrep)

#Analysis using imputed maternal and paternal genotypes

beta_imputed_mat <- vector(length = Nrep) #Fitting regression with genotypes imputed (maternal coefficient)

beta_imputed_pat <- vector(length = Nrep) #Fitting regression with genotypes imputed (paternal coefficient)

beta_imputed_fet <- vector(length = Nrep) #Fitting regression with genotypes imputed (fetal coefficient)

se_imputed_mat <- vector(length = Nrep)

se_imputed_pat <- vector(length = Nrep)

se_imputed_fet <- vector(length = Nrep)

pval_imputed_mat <- vector(length = Nrep)

pval_imputed_pat <- vector(length = Nrep)

pval_imputed_fet <- vector(length = Nrep)

#Analysis using only sibling genotypes

beta_sib <- vector(length = Nrep) #Fitting incorrect model including only siblings

se_sib <- vector(length = Nrep)

pval_sib <- vector(length = Nrep)

#ANOVA for full model against null model

pval_genotyped_omni <- vector(length = Nrep)

pval_imputed_omni <- vector(length = Nrep)

a <- sqrt(1/(2*p*q)) #Create genetic variable of variance one for maternal and fetal SNPs on X chromosome. Assume no dominance.

BetaM <- sqrt(Vm) #Path coefficient for maternal effect

BetaP <- sqrt(Vp/2) #Path coefficient for paternal effect

BetaF <- sqrt(Vf) #Path coefficient for fetal effect

if(Opp == TRUE) { #If Opp is true then maternal and fetal effects have opposite directions of effect

BetaF = -BetaF

}

Ve_x <- (1 - BetaM^2 - BetaF^2 - BetaP^2*2 - 2*0.5*BetaM*BetaF - 2*0.5*BetaP*BetaF*2) #Residual variance in trait for female sib

for(j in 1:Nrep) {

#Sample mothers' genotypes

Zm <- sample(x = c('AA','Aa','aa'), size = N, replace = TRUE, prob = c(p^2, 2*p*q, q^2))

#Sample fathers' genotypes

Zp <- sample(x = c('A','a'), size = N, replace = TRUE, prob = c(p, q))

Z_sib1x <- vector(length = N)

Z_sib2x <- vector(length = N)

Zmxx_imp <- vector(length = N) #Imputed vector of genotypes at mum's locus

Zpxx_imp <- vector(length = N) #Imputed vector of genotypes at dad's locus

#Simulate female sib 1 genotype

r <- runif(N)

for (i in 1:N) {

Z_sib1x[i] <- switch(paste(Zm[i],Zp[i]),

'AA A'='AA',

'AA a'='Aa',

'Aa A'=ifelse(r[i] <= 0.5, 'AA', 'Aa'),

'Aa a'=ifelse(r[i] <= 0.5, 'Aa', 'aa'),

'aa A'='Aa',

'aa a'='aa')

}

#Simulate female sib 2 genotype

r <- runif(N)

for (i in 1:N) {

Z_sib2x[i] <- switch(paste(Zm[i],Zp[i]),

'AA A'='AA',

'AA a'='Aa',

'Aa A'=ifelse(r[i] <= 0.5, 'AA', 'Aa'),

'Aa a'=ifelse(r[i] <= 0.5, 'Aa', 'aa'),

'aa A'='Aa',

'aa a'='aa')

}

#Change coding to 0, 1 and 2 in order to calculate allele frequencies

Z_sib1x_012 <- ifelse(Z_sib1x=='AA', 0,

ifelse(Z_sib1x=='Aa', 1, 2))

Z_sib2x_012 <- ifelse(Z_sib2x=='AA', 0,

ifelse(Z_sib2x=='Aa', 1, 2))

#Calculate observed allele frequency based on sibling genotypes

q_obs <- sum(Z_sib1x_012, Z_sib2x_012)/(2*N*2)

p_obs <- 1 - q_obs

#Impute parental genotypes from female sibs

#Calculate conditional probability of mother's genotypes given sibs genotypes

p_AA_AA_AA = (2*p_obs)/(p_obs+1) #P(Mum = AA | Sibx = AA and Sibx = AA)

p_Aa_AA_AA = (1-p_obs)/(p_obs+1) #P(Mum = Aa | Sibx = AA and Sibx = AA)

p_aa_AA_AA = 0 #P(Mum = aa | Sibx = AA and Sibx = AA)

p_AA_AA_Aa = 0 #P(Mum = AA | Sibx = AA and Sibx = Aa)

p_Aa_AA_Aa = 1 #P(Mum = Aa | Sibx = AA and Sibx = Aa)

p_aa_AA_Aa = 0 #P(Mum = aa | Sibx = AA and Sibx = Aa)

p_AA_AA_aa = 0 #P(Mum = AA | Sibx = AA and Sibx = aa)

p_Aa_AA_aa = 1 #P(Mum = Aa | Sibx = AA and Sibx = aa)

p_aa_AA_aa = 0 #P(Mum = aa | Sibx = AA and Sibx = aa)

p_AA_Aa_Aa = 2/3*p_obs #P(Mum = AA | Sibx = Aa and Sibx = Aa)

p_Aa_Aa_Aa = 1/3 #P(Mum = Aa | Sibx = Aa and Sibx = Aa)

p_aa_Aa_Aa = 2/3*q_obs #P(Mum = aa | Sibx = Aa and Sibx = Aa)

p_AA_aa_Aa = 0 #P(Mum = AA | Sibx = aa and Sibx = Aa)

p_Aa_aa_Aa = 1 #P(Mum = Aa | Sibx = aa and Sibx = Aa)

p_aa_aa_Aa = 0 #P(Mum = aa | Sibx = aa and Sibx = Aa)

p_AA_aa_aa = 0 #P(Mum = AA | Sibx = aa and Sibx = aa)

p_Aa_aa_aa = (1-q_obs)/(q_obs+1) #P(Mum = Aa | Sibx = aa and Sibx = aa)

p_aa_aa_aa = (2*q_obs)/(q_obs+1) #P(Mum = aa | Sibx = aa and Sibx = aa)

for (i in 1:N) {

Zmxx_imp[i] <- switch(paste(Z_sib1x[i], Z_sib2x[i]),

'AA AA'= -a*p_AA_AA_AA + 0*p_Aa_AA_AA + a*p_aa_AA_AA,

'AA Aa'= -a*p_AA_AA_Aa + 0*p_Aa_AA_Aa + a*p_aa_AA_Aa,

'AA aa'= -a*p_AA_AA_aa + 0*p_Aa_AA_aa + a*p_aa_AA_aa,

'Aa AA'= -a*p_AA_AA_Aa + 0*p_Aa_AA_Aa + a*p_aa_AA_Aa,

'Aa Aa'= -a*p_AA_Aa_Aa + 0*p_Aa_Aa_Aa + a*p_aa_Aa_Aa,

'Aa aa'= -a*p_AA_aa_Aa + 0*p_Aa_aa_Aa + a*p_aa_aa_Aa,

'aa AA'= -a*p_AA_aa_AA + 0*p_Aa_aa_AA + a*p_aa_aa_AA,

'aa Aa'= -a*p_AA_aa_Aa + 0*p_Aa_aa_Aa + a*p_aa_aa_Aa,

'aa aa'= -a*p_AA_aa_aa + 0*p_Aa_aa_aa + a*p_aa_aa_aa)

}

#Calculate conditional probability of father's genotypes given sib's genotype

p_dA_AA_AA <- 1 #P(Dad = A | Sibx = AA and Sibx = AA)

p_da_AA_AA <- 0 #P(Dad = a | Sibx = AA and Sibx = AA)

p_dA_AA_Aa <- 1 #P(Dad = A | Sibx = AA and Sibx = Aa)

p_da_AA_Aa <- 0 #P(Dad = a | Sibx = AA and Sibx = Aa)

p_dA_Aa_AA <- 1 #P(Dad = A | Sibx = Aa and Sibx = AA)

p_da_Aa_AA <- 0 #P(Dad = a | Sibx = Aa and Sibx = AA)

p_dA_Aa_Aa <- 2/3-1/3*p_obs #P(Dad = A | Sibx = Aa and Sibx = Aa)

p_da_Aa_Aa <- 2/3-1/3*q_obs #P(Dad = a | Sibx = Aa and Sibx = Aa)

p_dA_Aa_aa <- 0 #P(Dad = A | Sibx = Aa and Sibx = aa)

p_da_Aa_aa <- 1 #P(Dad = a | Sibx = Aa and Sibx = aa)

p_dA_aa_Aa <- 0 #P(Dad = A | Sibx = aa and Sibx = Aa)

p_da_aa_Aa <- 1 #P(Dad = a | Sibx = aa and Sibx = Aa)

p_dA_aa_aa <- 0 #P(Dad = A | Sibx = aa and Sibx = aa)

p_da_aa_aa <- 1 #P(Dad = a | Sibx = aa and Sibx = aa)

for (i in 1:N) {

Zpxx_imp[i] <- switch(paste(Z_sib1x[i], Z_sib2x[i]),

'AA AA'= -a*p_dA_AA_AA + a*p_da_AA_AA,

'Aa AA'= -a*p_dA_Aa_AA + a*p_da_Aa_AA,

'AA Aa'= -a*p_dA_AA_Aa + a*p_da_AA_Aa,

'Aa Aa'= -a*p_dA_Aa_Aa + a*p_da_Aa_Aa,

'aa Aa'= -a*p_dA_aa_Aa + a*p_da_aa_Aa,

'Aa aa'= -a*p_dA_Aa_aa + a*p_da_Aa_aa,

'aa aa'= -a*p_dA_aa_aa + a*p_da_aa_aa)

}

#Convert AA/Aa/aa to genetic value

Zm <- ifelse(Zm=='AA', -a, ifelse(Zm=='Aa', 0, a))

Zp <- ifelse(Zp=='A', -a, a)

Z_sib1x <- ifelse(Z_sib1x=='AA', -a, ifelse(Z_sib1x=='Aa', 0, a))

Z_sib2x <- ifelse(Z_sib2x=='AA', -a, ifelse(Z_sib2x=='Aa', 0, a))

#Create correlated error variables for sib 1 and sib 2 in the model

Sigma_xx <- matrix(c(Ve_x, rho, rho, Ve_x),2,2)

e_xx <- mvrnorm(n = N, mu = c(0, 0), Sigma_xx)

#Simulate offspring outcome

Y_sib1x_xx <- BetaM*Zm + BetaP*Zp + BetaF*Z_sib1x + e_xx[,1]

Y_sib2x_xx <- BetaM*Zm + BetaP*Zp + BetaF*Z_sib2x + e_xx[,2]

test <- data.frame(rbind(cbind(1:N, Y_sib1x_xx, Zm, Zp, Zmxx_imp, Zpxx_imp, Z_sib1x),

cbind(1:N, Y_sib2x_xx, Zm, Zp, Zmxx_imp, Zpxx_imp, Z_sib2x)))

colnames(test) <- c("fam", "Y_xx", "Zm", "Zp", "Zmxx_imp", "Zpxx_imp", "Z_xx")

#Run analyses

lmer_genotyped <- lmer(Y_xx ~ Zm + Zp + Z_xx + (1|fam), data = test, REML = FALSE); results_genotyped <- summary(lmer_genotyped)

lmer_imputed <- lmer(Y_xx ~ Zmxx_imp + Zpxx_imp + Z_xx + (1|fam), data = test, REML = FALSE); results_imputed <- summary(lmer_imputed)

lmer_sib <- lmer(Y_xx ~ Z_xx + (1|fam), data = test, REML = FALSE); results_sib <- summary(lmer_sib)

lmer_null <- lmer(Y_xx ~ (1|fam), data = test, REML = FALSE)

beta_genotyped_mat[j] <- results_genotyped$coefficient[2,1]

se_genotyped_mat[j] <- results_genotyped$coefficient[2,2]

pval_genotyped_mat[j] <- results_genotyped$coefficient[2,5]

beta_genotyped_pat[j] <- results_genotyped$coefficient[3,1]

se_genotyped_pat[j] <- results_genotyped$coefficient[3,2]

pval_genotyped_pat[j] <- results_genotyped$coefficient[3,5]

beta_genotyped_fet[j] <- results_genotyped$coefficient[4,1]

se_genotyped_fet[j] <- results_genotyped$coefficient[4,2]

pval_genotyped_fet[j] <- results_genotyped$coefficient[4,5]

pval_genotyped_omni[j] <- anova(lmer_genotyped,lmer_null)[2,8]

beta_imputed_mat[j] <- results_imputed$coefficient[2,1]

se_imputed_mat[j] <- results_imputed$coefficient[2,2]

pval_imputed_mat[j] <- results_imputed$coefficient[2,5]

beta_imputed_pat[j] <- results_imputed$coefficient[3,1]

se_imputed_pat[j] <- results_imputed$coefficient[3,2]

pval_imputed_pat[j] <- results_imputed$coefficient[3,5]

beta_imputed_fet[j] <- results_imputed$coefficient[4,1]

se_imputed_fet[j] <- results_imputed$coefficient[4,2]

pval_imputed_fet[j] <- results_imputed$coefficient[4,5]

pval_imputed_omni[j] <- anova(lmer_imputed,lmer_null)[2,8]

beta_sib[j] <- results_sib$coefficient[2,1]

se_sib[j] <- results_sib$coefficient[2,2]

pval_sib[j] <- results_sib$coefficient[2,5]

}

**Opposite sex sibling pairs at X chromosomal loci**

library(MASS)

library(lmerTest)

rm(list=ls())

Nrep = 1000 #Number of simulation replicates

N = 2000 #Sample size

Vm = 0.001 #Variance explained by maternal effect

Vp = 0.001 #Variance explained by paternal effect

Vf = 0.001 #Variance explained by fetal effect

p = 0.5 #Increaser allele frequency

Opp = FALSE #Boolean variable to denote whether maternal and fetal effects in opposite directions (TRUE = opposite directions)

rho = 0.2 #Covariance between sibling residual variances

q <- 1-p #Decreaser allele frequency

#Create vectors to save results from regression analyses

#For analysis using simulated genotypes:

beta_genotyped_mat <- vector(length = Nrep) #Fitting regression with maternal and paternal genotypes known (maternal coefficient)

beta_genotyped_pat <- vector(length = Nrep) #Fitting regression with maternal and paternal genotypes known (paternal coefficient)

beta_genotyped_fet <- vector(length = Nrep) #Fitting regression with maternal and paternal genotypes known (fetal coefficient)

se_genotyped_mat <- vector(length = Nrep)

se_genotyped_pat <- vector(length = Nrep)

se_genotyped_fet <- vector(length = Nrep)

pval_genotyped_mat <- vector(length = Nrep)

pval_genotyped_pat <- vector(length = Nrep)

pval_genotyped_fet <- vector(length = Nrep)

#Analysis using imputed maternal and paternal genotypes:

beta_imputed_mat <- vector(length = Nrep) #Fitting regression with genotypes imputed (maternal coefficient)

beta_imputed_pat <- vector(length = Nrep) #Fitting regression with genotypes imputed (paternal coefficient)

beta_imputed_fet <- vector(length = Nrep) #Fitting regression with genotypes imputed (fetal coefficient)

se_imputed_mat <- vector(length = Nrep)

se_imputed_pat <- vector(length = Nrep)

se_imputed_fet <- vector(length = Nrep)

pval_imputed_mat <- vector(length = Nrep)

pval_imputed_pat <- vector(length = Nrep)

pval_imputed_fet <- vector(length = Nrep)

#Analysis using only half-sib genotypes:

beta_fet <- vector(length = Nrep) #Fitting incorrect model to just sibs

se_fet <- vector(length = Nrep)

pval_fet <- vector(length = Nrep)

#ANOVA for full model against null model

pval_imputed_omni <- vector(length = Nrep)

pval_genotyped_omni <- vector(length = Nrep)

a <- sqrt(1/(2*p*q)) #Create genetic variable of variance one for maternal SNPs on X chromosome. Assume no dominance.

BetaM <- sqrt(Vm) #Path coefficient for maternal effect

BetaP <- sqrt(Vp/2) #Path coefficient for paternal effect

BetaF <- sqrt(Vf/2) #Path coefficient for fetal effect. Assume **male** genotype

if(Opp == TRUE) { #If Opp is true then maternal and fetal effects have opposite directions of effect

BetaF = -BetaF

}

Ve_x <- (1 - BetaM^2 - BetaF^2 - BetaP^2*2 - 2*0.5*BetaM*BetaF - 2*0.5*BetaP*BetaF*2) #Residual variance in trait for female sib

Ve_y <- (1 - BetaM^2 - BetaF^2*2 - BetaP^2*2 - 2*1*BetaM*BetaF) #Residual variance in trait for male sib

for(j in 1:Nrep) {

#Sample mothers' genotypes

Zm <- sample(x = c('AA','Aa','aa'), size = N, replace = TRUE, prob = c(p^2, 2*p*q, q^2))

#Sample fathers' genotypes

Zp <- sample(x = c('A','a'), size = N, replace = TRUE, prob = c(p, q))

Z_sibx <- vector(length = N)

Z_siby <- vector(length = N)

Zmxy_imp <- vector(length = N) #Imputed vector of genotypes at mum's locus

Zpxy_imp <- vector(length = N) #Imputed vector of genotypes at dad's locus

#Simulate female sib genotype

r <- runif(N)

for (i in 1:N) {

Z_sibx[i] <- switch(paste(Zm[i],Zp[i]),

'AA A'='AA',

'AA a'='Aa',

'Aa A'=ifelse(r[i] <= 0.5, 'AA', 'Aa'),

'Aa a'=ifelse(r[i] <= 0.5, 'Aa', 'aa'),

'aa A'='Aa',

'aa a'='aa')

}

#Simulate male sib genotype

r <- runif(N)

for (i in 1:N) {

Z_siby[i] <- switch(Zm[i],

'AA'='A',

'Aa'=ifelse(r[i] <= 0.5, 'A', 'a'),

'aa'='a')

}

#Change coding to 0, 1 and 2 in order to calculate allele frequencies

Z_sibx_012 <- ifelse(Z_sibx=='AA', 0, ifelse(Z_sibx=='Aa', 1, 2))

Z_siby_01 <- ifelse(Z_siby=='A', 0, 1)

#Calculate observed allele frequency based on female sibling one's genotypes

q_obs <- sum(Z_sibx_012)/(2*N)

p_obs <- 1 - q_obs

#Impute parental genotypes from male-female siblings

#Calculate conditional probability of mother's genotypes given sibling genotypes

p_AA_A_AA = (2*p_obs)/(p_obs+1) #P(Mum = AA | Siby = A and Sibx = AA)

p_Aa_A_AA = (1-p_obs)/(p_obs+1) #P(Mum = Aa | Siby = A and Sibx = AA)

p_aa_A_AA = 0 #P(Mum = aa | Siby = A and Sibx = AA)

p_AA_A_Aa = (2*p_obs)/(2*p_obs+1) #P(Mum = AA | Siby = A and Sibx = Aa)

p_Aa_A_Aa = 1/(2*p_obs+1) #P(Mum = Aa | Siby = A and Sibx = Aa)

p_aa_A_Aa = 0 #P(Mum = aa | Siby = A and Sibx = Aa)

p_AA_A_aa = 0 #P(Mum = AA | Siby = A and Sibx = aa)

p_Aa_A_aa = 1 #P(Mum = Aa | Siby = A and Sibx = aa)

p_aa_A_aa = 0 #P(Mum = aa | Siby = A and Sibx = aa)

p_AA_a_AA = 0 #P(Mum = AA | Siby = a and Sibx = AA)

p_Aa_a_AA = 1 #P(Mum = Aa | Siby = a and Sibx = AA)

p_aa_a_AA = 0 #P(Mum = aa | Siby = a and Sibx = AA)

p_AA_a_Aa = 0 #P(Mum = AA | Siby = a and Sibx = Aa)

p_Aa_a_Aa = 1/(2*q_obs+1) #P(Mum = Aa | Siby = a and Sibx = Aa)

p_aa_a_Aa = (2*q_obs)/(2*q_obs+1) #P(Mum = aa | Siby = a and Sibx = Aa)

p_AA_a_aa = 0 #P(Mum = AA | Siby = a and Sibx = aa)

p_Aa_a_aa = (1-q_obs)/(q_obs+1) #P(Mum = Aa | Siby = a and Sibx = aa)

p_aa_a_aa = (2*q_obs)/(q_obs+1) #P(Mum = aa | Siby = a and Sibx = aa)

p_AA_AA_A <- p_AA_A_AA

p_Aa_AA_A <- p_Aa_A_AA

p_aa_AA_A <- p_aa_A_AA

p_AA_Aa_A <- p_AA_A_Aa

p_Aa_Aa_A <- p_Aa_A_Aa

p_aa_Aa_A <- p_aa_A_Aa

p_AA_aa_A <- p_AA_A_aa

p_Aa_aa_A <- p_Aa_A_aa

p_aa_aa_A <- p_aa_A_aa

p_AA_AA_a <- p_AA_a_AA

p_Aa_AA_a <- p_Aa_a_AA

p_aa_AA_a <- p_aa_a_AA

p_AA_Aa_a <- p_AA_a_Aa

p_Aa_Aa_a <- p_Aa_a_Aa

p_aa_Aa_a <- p_aa_a_Aa

p_AA_aa_a <- p_AA_a_aa

p_Aa_aa_a <- p_Aa_a_aa

p_aa_aa_a <- p_aa_a_aa

for (i in 1:N) {

Zmxy_imp[i] <- switch(paste(Z_siby[i], Z_sibx[i]),

'A AA'= -a*p_AA_A_AA + 0*p_Aa_A_AA + a*p_aa_A_AA,

'A Aa'= -a*p_AA_A_Aa + 0*p_Aa_A_Aa + a*p_aa_A_Aa,

'A aa'= -a*p_AA_A_aa + 0*p_Aa_A_aa + a*p_aa_A_aa,

'a AA'= -a*p_AA_a_AA + 0*p_Aa_a_AA + a*p_aa_a_AA,

'a Aa'= -a*p_AA_a_Aa + 0*p_Aa_a_Aa + a*p_aa_a_Aa,

'a aa'= -a*p_AA_a_aa + 0*p_Aa_a_aa + a*p_aa_a_aa)

}

#Calculate conditional probability of father's genotypes given sib's genotype

p_A_A_AA <- 1 #P(Dad = A | Siby = A and Sibx = AA)

p_a_A_AA <- 0 #P(Dad = a | Siby = A and Sibx = AA)

p_A_A_Aa <- p_obs/(2*p_obs+1) #P(Dad = A | Siby = A and Sibx = Aa)

p_a_A_Aa <- (p_obs+1)/(2*p_obs+1) #P(Dad = a | Siby = A and Sibx = Aa)

p_A_A_aa <- 0 #P(Dad = A | Siby = A and Sibx = aa)

p_a_A_aa <- 1 #P(Dad = a | Siby = A and Sibx = aa)

p_A_a_AA <- 1 #P(Dad = A | Siby = a and Sibx = AA)

p_a_a_AA <- 0 #P(Dad = a | Siby = a and Sibx = AA)

p_A_a_Aa <- (q_obs+1)/(2*q_obs+1) #P(Dad = A | Siby = a and Sibx = Aa)

p_a_a_Aa <- q_obs/(2*q_obs+1) #P(Dad = a | Siby = a and Sibx = Aa)

p_A_a_aa <- 0 #P(Dad = A | Siby = a and Sibx = aa)

p_a_a_aa <- 1 #P(Dad = a | Siby = a and Sibx = aa)

for (i in 1:N) {

Zpxy_imp[i] <- switch(paste(Z_siby[i], Z_sibx[i]),

'A AA'= -a*p_A_A_AA + a*p_a_A_AA,

'A Aa'= -a*p_A_A_Aa + a*p_a_A_Aa,

'A aa'= -a*p_A_A_aa + a*p_a_A_aa,

'a AA'= -a*p_A_a_AA + a*p_a_a_AA,

'a Aa'= -a*p_A_a_Aa + a*p_a_a_Aa,

'a aa'= -a*p_A_a_aa + a*p_a_a_aa)

}

#Convert AA/Aa/aa to genetic value -a/0/a

Zm <- ifelse(Zm=='AA', -a, ifelse(Zm=='Aa', 0, a))

Zp <- ifelse(Zp=='A', -a, a)

Z_sibx <- ifelse(Z_sibx=='AA', -a, ifelse(Z_sibx=='Aa', 0, a))

Z_siby <- ifelse(Z_siby=='A', -a, a)

#Create correlated error variables for sib 1 and sib 2 in the model

Sigma_xy <- matrix(c(Ve_x, rho, rho, Ve_y),2,2) #Female-male sibs

e_xy <- mvrnorm(n = N, mu = c(0, 0), Sigma_xy)

#Simulate offspring outcome

Y_sibx_xy <- BetaM*Zm + BetaP*Zp + BetaF*Z_sibx + e_xy[,1]

Y_siby_xy <- BetaM*Zm + BetaP*Zp + BetaF*Z_siby + e_xy[,2]

testxy <- data.frame(rbind(cbind(1:N, Y_sibx_xy, Zm, Zp, Zmxy_imp, Zpxy_imp, Z_sibx),

cbind(1:N, Y_siby_xy, Zm, Zp, Zmxy_imp, Zpxy_imp, Z_siby)))

colnames(testxy) <- c("fam", "Y_xy", "Zm", "Zp", "Zmxy_imp", "Zpxy_imp", "Z_xy")

#Run analyses

lmer_genotyped <- lmer(Y_xy ~ Zm + Zp + Z_xy + (1|fam), data = testxy, REML = FALSE); results_genotyped <- summary(lmer_genotyped)

lmer_imputed <- lmer(Y_xy ~ Zmxy_imp + Zpxy_imp + Z_xy + (1|fam), data = testxy, REML = FALSE); results_imputed <- summary(lmer_imputed)

lmer_sib <- lmer(Y_xy ~ Z_xy + (1|fam), data = testxy, REML = FALSE); results_sib <- summary(lmer_sib)

lmer_null <- lmer(Y_xy ~ (1|fam), data = testxy, REML = FALSE)

beta_genotyped_mat[j] <- results_genotyped$coefficient[2,1]

se_genotyped_mat[j] <- results_genotyped$coefficient[2,2]

pval_genotyped_mat[j] <- results_genotyped$coefficient[2,5]

beta_genotyped_pat[j] <- results_genotyped$coefficient[3,1]

se_genotyped_pat[j] <- results_genotyped$coefficient[3,2]

pval_genotyped_pat[j] <- results_genotyped$coefficient[3,5]

beta_genotyped_fet[j] <- results_genotyped$coefficient[4,1]

se_genotyped_fet[j] <- results_genotyped$coefficient[4,2]

pval_genotyped_fet[j] <- results_genotyped$coefficient[4,5]

pval_genotyped_omni[j] <- anova(lmer_genotyped,lmer_null)[2,8]

beta_imputed_mat[j] <- results_imputed$coefficient[2,1]

se_imputed_mat[j] <- results_imputed$coefficient[2,2]

pval_imputed_mat[j] <- results_imputed$coefficient[2,5]

beta_imputed_pat[j] <- results_imputed$coefficient[3,1]

se_imputed_pat[j] <- results_imputed$coefficient[3,2]

pval_imputed_pat[j] <- results_imputed$coefficient[3,5]

beta_imputed_fet[j] <- results_imputed$coefficient[4,1]

se_imputed_fet[j] <- results_imputed$coefficient[4,2]

pval_imputed_fet[j] <- results_imputed$coefficient[4,5]

pval_imputed_omni[j] <- anova(lmer_imputed,lmer_null)[2,8]

beta_fet[j] <- results_sib$coefficient[2,1]

se_fet[j] <- results_sib$coefficient[2,2]

pval_fet[j] <- results_sib$coefficient[2,5]

}
