# Supplementary figures and images for: Estimating indirect parental genetic effects on offspring phenotypes using virtual parental genotypes derived from sibling and half sibling pairs
Source: PLoS Genet. 2020 Oct 26;16(10):e1009154. doi: 10.1371/journal.pgen.1009154 (PMC7646364; doi:10.1371/journal.pgen.1009154)

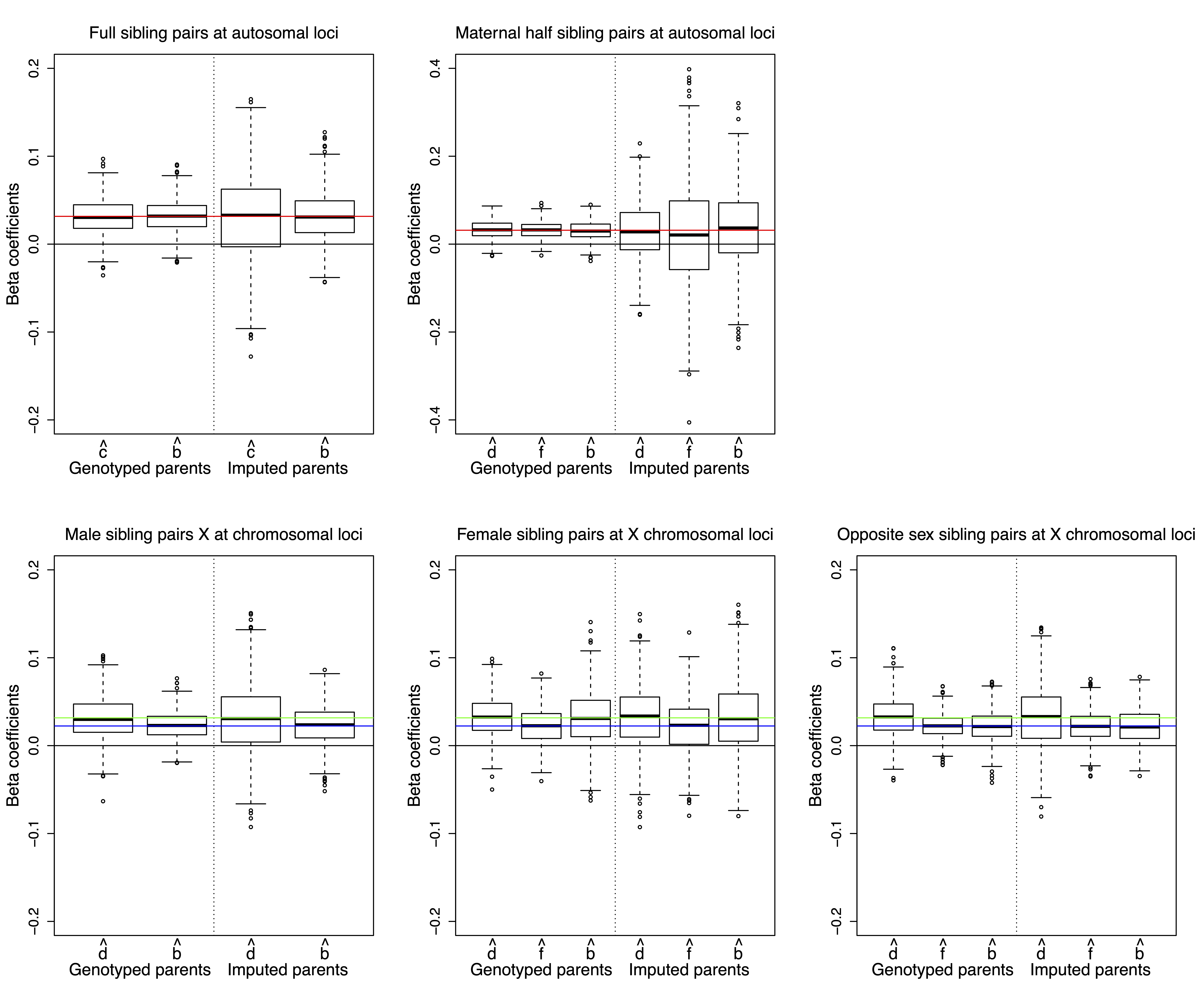

Supplement: S1 Fig — Comparison of maternal (d), paternal (f) and offspring (b) genetic effects estimated using genotyped parental genotypes versus imputed parental genotypes in simulations. We simulated genetic effects accounting for 0.1% of the variance in the offspring trait (b2 = d2 = f2 = 0.1%), a trait decreasing allele frequency of p = 0.1, and shared residual variance of φ2 = 0.2. For all simulations we used N = 2000 sibling pairs/half sibling pairs, and 1000 replications. In the case of autosomal loci, red lines indicate the expected beta coefficients for parental (c) and offspring genetic effects (b) in full sibling pairs, and the expected beta coefficients for maternal (d), paternal (f), and offspring genetic effects (b) in half sibling pairs. Blue lines indicate the expected beta coefficients for paternal (f) and male fetal (b) effects at X chromosomal loci. Green lines indicate the expected beta coefficients for maternal (d) and female fetal (b) effect at X chromosomal loci. For opposite sex sibling pairs at X chromosomal loci, we simulated the fetal effect (b) to be the same for both siblings assuming using male genotypes. The full omnibus model was simulated and fitted in all simulations. R codes implementing the simulations are provided in the S1 Text. (PNG) [file pgen.1009154.s002.png]
