# Supplementary material for: Estimating indirect parental genetic effects on offspring phenotypes using virtual parental genotypes derived from sibling and half sibling pairs
Source: PLoS Genet. 2020 Oct 26;16(10):e1009154. doi: 10.1371/journal.pgen.1009154 (PMC7646364; doi:10.1371/journal.pgen.1009154)
Supplement: S1 Table — (DOCX) [file pgen.1009154.s003.docx]

**S1 Table.** Probabilities (P) and expected dosages for imputed parental genotypes conditional on observed sibling pair genotypes at autosomal loci. The symbol *q* = 1 – *p* denotes the frequency of the trait increasing allele *a*. The expected parental dosage refers to the expected number of trait increasing alleles *a*.

|  | **Parental Genotype Probabilities Conditional on Observed Sibling Genotypes** | | |  |
| --- | --- | --- | --- | --- |
|  | **P(*AA*)** | **P(*Aa*)** | **P(*aa*)** | **Expected Parental**  **Dosage** |
| **Sibling Genotypes** |  |  |  |  |
| ***AA*, *AA*** | $\frac{2-2q}{2-q}$ | $\frac{q}{2-q}$ | 0 | $\frac{q}{2-q}$ |
| ***AA*, *Aa*** | $\frac{1-q}{2-q}$ | $\frac{1}{2-q}$ | 0 | $\frac{1}{2-q}$ |
| ***AA*, *aa*** | 0 | 1 | 0 | 1 |
| ***Aa*, *Aa*** | $\frac{0.5\left( q-1 \right)(q+1)}{q^{2}-q-1}$ | $\frac{-0.5}{q^{2}-q-1}$ | $\frac{0.5\left( q-2 \right)q}{q^{2}-q-1}$ | $\frac{q^{2}-2q-0.5}{q^{2}-q-1}$ |
| ***Aa*, *aa*** | 0 | $\frac{1}{q+1}$ | $\frac{q}{q+1}$ | $\frac{2q+1}{q+1}$ |
| ***aa*, *aa*** | 0 | $\frac{1-q}{q+1}$ | $\frac{2q}{q+1}$ | $\frac{3q+1}{q+1}$ |
