# Supplementary material for: Estimating indirect parental genetic effects on offspring phenotypes using virtual parental genotypes derived from sibling and half sibling pairs
Source: PLoS Genet. 2020 Oct 26;16(10):e1009154. doi: 10.1371/journal.pgen.1009154 (PMC7646364; doi:10.1371/journal.pgen.1009154)
Supplement: S2 Table — (DOCX) [file pgen.1009154.s004.docx]

**S2 Table.** Probabilities (P) and expected dosages for imputed parental genotypes conditional on observed sibling pair genotypes at non-pseudoautosomal X chromosome loci. The symbol *q = 1- p* denotes the frequency of the trait increasing allele *a*. The expected parental dosage refers to the expected number of trait increasing alleles *a*. For opposite sex pairs, the genotype of the male sibling is given first. NA= not applicable due to the genotype combination being impossible.

|  | **Maternal Genotype** | | | | **Paternal Genotype** | | |
| --- | --- | --- | --- | --- | --- | --- | --- |
| **Sibling Genotypes** |  |  |  |  |  |  |  |
| **Male Pairs** | **P(*AA*)** | **P(*Aa*)** | **P(*aa*)** | **Expected Dosage** | **P(*A*)** | **P(*a*)** | **Expected Dosage** |
| ***A*, *A*** | $\frac{2-2q}{2-q}$ | $\frac{q}{2-q}$ | 0 | $\frac{q}{2-q}$ | $1-q$ | $q$ | $2q$ |
| ***A*, *a*** | 0 | 1 | 0 | 1 | $1-q$ | $q$ | $2q$ |
| ***a*, *a*** | 0 | $\frac{1-q}{1+q}$ | $\frac{2q}{q+1}$ | $\frac{3q+1}{q+1}$ | $1-q$ | $q$ | $2q$ |
|  | | | | | | | |
| **Female Pairs** | **P(*AA*)** | **P(*Aa*)** | **P(*aa*)** | **Expected Dosage** | **P(*A*)** | **P(*a*)** | **Expected Dosage** |
| ***AA*, *AA*** | $\frac{2-2q}{2-q}$ | $\frac{q}{2-q}$ | 0 | $\frac{q}{2-q}$ | 1 | 0 | 0 |
| ***AA*, *Aa*** | 0 | 1 | 0 | 1 | 1 | 0 | 0 |
| ***AA*, *aa*** | NA | NA | NA | NA | NA | NA | NA |
| ***Aa*, *Aa*** | $\frac{2-2q}{3}$ | $\frac{1}{3}$ | $\frac{2q}{3}$ | $\frac{1+4q}{3}$ | $\frac{1+q}{3}$ | $\frac{2-q}{3}$ | $\frac{4-2q}{3}$ |
| ***Aa*, *aa*** | 0 | 1 | 0 | 1 | 0 | 1 | 2 |
| ***aa*, *aa*** | 0 | $\frac{1-q}{q+1}$ | $\frac{2q}{q+1}$ | $\frac{3q+1}{q+1}$ | 0 | 1 | 2 |
|  | | | | | | | |
| **Opposite Sex Pairs** | **P(*AA*)** | **P(*Aa*)** | **P(*aa*)** | **Expected Dosage** | **P(*A*)** | **P(*a*)** | **Expected Dosage** |
| ***A*, *AA*** | $\frac{2-2q}{2-q}$ | $\frac{q}{2-q}$ | 0 | $\frac{q}{2-q}$ | 1 | 0 | 0 |
| ***A*, *Aa*** | $\frac{2-2q}{3-2q}$ | $\frac{1}{3-2q}$ | 0 | $\frac{1}{3-2q}$ | $\frac{1-q}{3-2q}$ | $\frac{2-q}{3-2q}$ | $\frac{4-2q}{3-2q}$ |
| ***A*, *aa*** | 0 | 1 | 0 | 1 | 0 | 1 | 2 |
| ***a*, *AA*** | 0 | 1 | 0 | 1 | 1 | 0 | 0 |
| ***a*, *Aa*** | 0 | $\frac{1}{2q+1}$ | $\frac{2q}{2q+1}$ | $\frac{4q+1}{2q+1}$ | $\frac{1+q}{2q+1}$ | $\frac{q}{2q+1}$ | $\frac{2q}{2q+1}$ |
| ***a*, *aa*** | 0 | $\frac{1-q}{q+1}$ | $\frac{2q}{q+1}$ | $\frac{3q+1}{q+1}$ | 0 | 1 | 2 |
