# Supplementary material for: Estimating indirect parental genetic effects on offspring phenotypes using virtual parental genotypes derived from sibling and half sibling pairs
Source: PLoS Genet. 2020 Oct 26;16(10):e1009154. doi: 10.1371/journal.pgen.1009154 (PMC7646364; doi:10.1371/journal.pgen.1009154)
Supplement: S3 Table — (DOCX) [file pgen.1009154.s005.docx]

**S3 Table.** Genotype probabilities (P) and expected dosages for imputed genotypes of shared parent conditional on observed half sibling pair genotypes at autosomal loci. The symbol *q* = 1- *p* denotes the frequency of the trait increasing allele *a*. The expected parental dosage refers to the expected number of trait increasing alleles *a*.

|  | **Shared Parental Genotype** | | |  |
| --- | --- | --- | --- | --- |
| **Half Sibling**  **Genotype** | **P(*AA*)** | **P(*Aa*)** | **P(*aa*)** | **Expected Dosage** |
| ***AA*, *AA*** | $\frac{2-2q}{2-q}$ | $\frac{q}{2-q}$ | $0$ | $\frac{q}{2-q}$ |
| ***AA*, *Aa*** | $\frac{2-2q}{3-2q}$ | $\frac{1}{3-2q}$ | $0$ | $\frac{1}{3-2q}$ |
| ***AA*, *aa*** | $0$ | $1$ | $0$ | 1 |
| ***Aa*, *Aa*** | $\frac{2q(1-q)}{-4q^{2}+4q+1}$ | $\frac{1}{-4q^{2}+4q+1}$ | $\frac{2q(1-q)}{-4q^{2}+4q+1}$ | 1 |
| ***Aa*, *aa*** | $0$ | $\frac{1}{2q+1}$ | $\frac{2q}{2q+1}$ | $\frac{4q+1}{2q+1}$ |
| ***aa*, *aa*** | $0$ | $\frac{1-q}{q+1}$ | $\frac{2q}{q+1}$ | $\frac{3q+1}{q+1}$ |
| ***Aa*, *AA*** | $\frac{2-2q}{3-2q}$ | $\frac{1}{3-2q}$ | $0$ | $\frac{1}{3-2q}$ |
| ***aa*, *AA*** | $0$ | $1$ | $0$ | 1 |
| ***aa*, *Aa*** | $0$ | $\frac{1}{2q+1}$ | $\frac{2q}{2q+1}$ | $\frac{4q+1}{2q+1}$ |
