# Supplementary material for: Estimating indirect parental genetic effects on offspring phenotypes using virtual parental genotypes derived from sibling and half sibling pairs
Source: PLoS Genet. 2020 Oct 26;16(10):e1009154. doi: 10.1371/journal.pgen.1009154 (PMC7646364; doi:10.1371/journal.pgen.1009154)
Supplement: S4 Table — (DOCX) [file pgen.1009154.s006.docx]

**S4 Table.** Genotype probabilities (P) and expected dosages for imputed genotypes of unshared parents conditional on observed maternal half sibling pair genotypes at autosomal loci. The symbol *q* = 1- *p* denotes the frequency of the trait increasing allele *a*. The expected parental dosage refers to the expected number of trait increasing alleles *a*. In the case of paternal half siblings, the probabilities and expected dosages are the same for the mother of the first half sibling and the mother of the second half sibling.

|  | **Father of First Half Sibling** | | | | **Father of Second Half Sibling** | | | |
| --- | --- | --- | --- | --- | --- | --- | --- | --- |
|  | **P(*AA*)** | **P(*Aa*)** | **P(*aa*)** | **Expected Dosage** | **P(*AA*)** | **P(*Aa*)** | **P(*aa*)** | **Expected Dosage** |
| ***AA*, *AA*** | $1-q$ | $q$ | $0$ | $q$ | $1-q$ | $q$ | 0 | $q$ |
| ***AA*, *Aa*** | $1-q$ | $q$ | $0$ | $q$ | $\frac{{(1-q)}^{2}}{3-2q}$ | $\frac{2-2q}{3-2q}$ | $\frac{q(2-q)}{3-2q}$ | $\frac{{-2q}^{2}+2q+2}{3-2q}$ |
| ***AA*, *aa*** | $1-q$ | $q$ | $0$ | $q$ | 0 | $1-q$ | $q$ | $q+1$ |
| ***Aa*, *Aa*** | $\frac{2q^{3}-3q^{2}+1}{-4q^{2}+4q+1}$ | $\frac{-4q^{2}+4q}{-4q^{2}+4q+1}$ | $\frac{3q^{2}-2q^{3}}{-4q^{2}+4q+1}$ | $\frac{-4q^{3}+2q^{2}+4q}{-4q^{2}+4q+1}$ | $\frac{2q^{3}-3q^{2}+1}{-4q^{2}+4q+1}$ | $\frac{-4q^{2}+4q}{-4q^{2}+4q+1}$ | $\frac{3q^{2}-2q^{3}}{-4q^{2}+4q+1}$ | $\frac{-4q^{3}+2q^{2}+4q}{-4q^{2}+4q+1}$ |
| ***Aa*, *aa*** | $\frac{1-q^{2}}{2q+1}$ | $\frac{2q}{2q+1}$ | $\frac{q^{2}}{2q+1}$ | $\frac{{2q}^{2}+2q}{2q+1}$ | 0 | $1-q$ | $q$ | $q+1$ |
| ***aa*, *aa*** | $0$ | $1-q$ | $q$ | $q+1$ | 0 | $1-q$ | $q$ | $q+1$ |
| ***Aa*, *AA*** | $\frac{{(1-q)}^{2}}{3-2q}$ | $\frac{2(1-q)}{3-2q}$ | $\frac{q(2-q)}{3-2q}$ | $\frac{{-2q}^{2}+2q+2}{3-2q}$ | $1-q$ | $q$ | 0 | $q$ |
| ***aa*, *AA*** | $0$ | $1-q$ | $q$ | $q+1$ | $1-q$ | $q$ | 0 | $q$ |
| ***aa*, *Aa*** | $0$ | $1-q$ | $q$ | $q+1$ | $\frac{1-q^{2}}{2q+1}$ | $\frac{2q}{2q+1}$ | $\frac{q^{2}}{2q+1}$ | $\frac{{2q}^{2}+2q}{2q+1}$ |
