# Supplementary material for: Estimating indirect parental genetic effects on offspring phenotypes using virtual parental genotypes derived from sibling and half sibling pairs
Source: PLoS Genet. 2020 Oct 26;16(10):e1009154. doi: 10.1371/journal.pgen.1009154 (PMC7646364; doi:10.1371/journal.pgen.1009154)
Supplement: S17 Table — (DOCX) [file pgen.1009154.s019.docx]

**S17 Table.** Computational performance of IMPISH. Reported runtimes of IMPISH for imputation and performing association analyses. All tests were performed in the same computing environment with 256 GB memory and 1 CPU core with solid-state disk in one computer node.

| Number of SNPs | Sample size  (Sibling pairs) | Imputation (hours) | Association analysis (hours) |
| --- | --- | --- | --- |
| 500,000 | 1,000 | 0.64 | 0.75 |
| 500,000 | 5,000 | 4.01 | 13.70 |
| 500,000 | 10,000 | 9.21 | 58.07 |
| 500,000 | 20,000 | 18.05 | 175.45 |
