# Supplementary material for: Estimating indirect parental genetic effects on offspring phenotypes using virtual parental genotypes derived from sibling and half sibling pairs
Source: PLoS Genet. 2020 Oct 26;16(10):e1009154. doi: 10.1371/journal.pgen.1009154 (PMC7646364; doi:10.1371/journal.pgen.1009154)
Supplement: S18 Table — (DOCX) [file pgen.1009154.s020.docx]

**S18 Table.** Marginal probability of Sibling Pair Genotypes.

| Sib1  Genotype  (G_1_) | Sib2  Genotype  (G_2_) | P(G_1_,G_2_\|IBD=0) | P(G_1_,G_2_\|IBD=1) | P(G_1_,G_2_\|IBD=2) | P(G_1_,G_2_) |
| --- | --- | --- | --- | --- | --- |
| *AA* | *AA* | *p^4^* | *p^3^* | *p^2^* | ¼*p^4^*+ ½*p^3^* + ¼*p^2^* |
| *AA* | *Aa* | *2p^3^q* | *p^2^q* | 0 | ½*p^3^q* + ½*p^2^q* |
| *AA* | *aa* | *p^2^q^2^* | 0 | 0 | ¼*p^2^q^2^* |
| *Aa* | *AA* | *2p^3^q* | *p^2^q* | 0 | ½*p^3^q* + ½*p^2^q* |
| *Aa* | *Aa* | *4p^2^q^2^* | *pq* | *2pq* | ¼*p^2^q^2^* + ½*pq* + ½*pq* |
| *Aa* | *aa* | *2pq^3^* | *pq^2^* | 0 | ½*pq^3^* + ½*pq^2^* |
| *aa* | *AA* | *p^2^q^2^* | 0 | 0 | ¼*p^2^q^2^* |
| *aa* | *Aa* | *2pq^3^* | *pq^2^* | 0 | ½*pq^3^* + ½*pq^2^* |
| *aa* | *aa* | *q^4^* | *q^3^* | *q^2^* | ¼*q^4^* + ½*q^3^* + ¼*q^2^* |
